# Photocatalytic proximity labeling for the identification of G-quadruplex DNA-interacting proteins

**DOI:** 10.1038/s42004-026-02170-9

**Published:** 2026-08-27

**Authors:** Ahmed Mostafa Abdelhady, Shinichi Sato, Tatsuki Masuzawa, Keishi Deguchi, Mizuki Oba, Takemaru Sato, Nodoka Mase, Toshifumi Yamanaka, Jamila Abbas Osman, Maho Kato, Keita Nakane, Zhengyi Liu, Kazuki Kuwahara, Satoru Nagatoishi, Kouhei Tsumoto, Fumi Nagatsugi, Takanori Oyoshi, Kazumitsu Onizuka

**Affiliations:** 1 Institute of Multidisciplinary Research for Advanced Materials, Tohoku University, Sendai, Japan; 2 Department of Chemistry, Graduate School of Science, Tohoku University, Sendai, Japan; 3 Department of Chemistry, Faculty of Science, Al-Azhar University, Cairo, Egypt; 4 Frontier Research Institute for Interdisciplinary Sciences and Graduate School of Life Sciences, Tohoku University, Sendai, Japan; 5 Graduate School of Integrated Science and Technology, Shizuoka University, Shizuoka, Japan; 6 Department of Bioengineering, School of Engineering, The University of Tokyo, Tokyo, Japan; 7 Graduate School of Science and Technology, Shizuoka University, Shizuoka, Japan; 8 Research Institute of Green Science and Technology, Shizuoka University, Shizuoka, Japan; 9 Division for the Establishment of Frontier Sciences of Organization for Advanced Studies, Tohoku University, Sendai, Japan

**Keywords:** DNA, Proteomics, Nucleic acids

## Abstract

DNA–protein interactions play an essential role in fundamental cellular biological processes. Herein, we report a photocatalytic proximity labeling approach for the broad screening of G-quadruplex (G4)-interacting proteins (G4IPs) using human telomere G4 DNA modified with a photocatalyst (Ruthenium complex or BODIPY). We confirmed efficient labeling of unwinding protein 1 (UP1), a model of G4-interacting protein, with 1-methyl-4-arylurazole (MAUra) as a labeling reagent under blue light irradiation. We applied this labeling approach to nuclear extract proteins and identified labeled proteins using quantitative proteomics analysis. Numerous unknown G4 binding protein candidates were identified. Notably, the hexokinase-1 (HK1) protein was identified as a G4IP, and its selective binding toward G4 DNA was confirmed. This finding could be used to highlight the essential subcellular G4-mediated functions of HK1. The proposed labeling approach is a promising tool for investigating the protein interactions of the higher-order structural motifs and functional nucleic acids.

**Figure:**
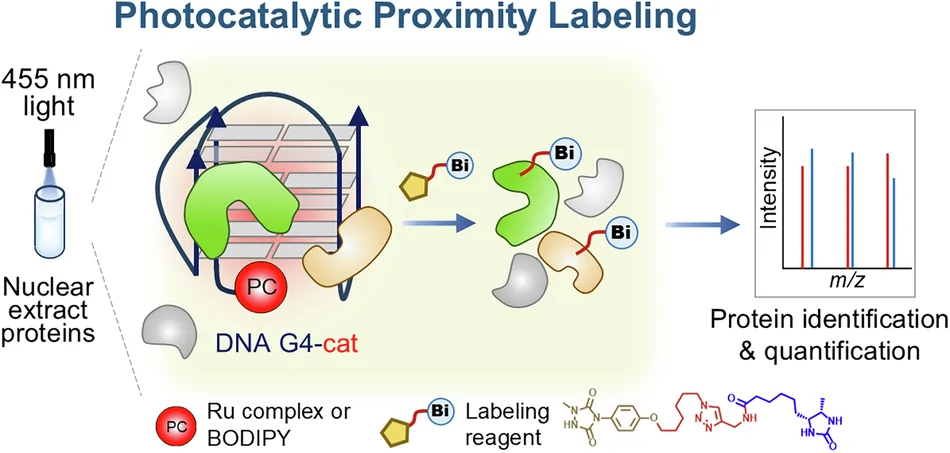

## Abstract (web summary)

Protein–nucleic acid interactions are pivotal in regulating cellular processes, yet identifying weakly bound proteins remains challenging. Here, the authors introduce a photocatalyst-modified DNA G-quadruplex approach for precise profiling of G4-interacting proteins, revealing insights into their roles in biological processes and advancing our understanding of nucleic acid regulation.

## Introduction

Protein–nucleic acid interactions play a key role in the regulation of fundamental nucleic acid–related cellular processes, including gene expression, DNA replication, transcription, and repair^1^. The identification of protein complexes involved in these interactions is crucial for determining the biological relevance of these proteins^2,3^. Many biochemical assays have been developed to investigate specific nucleic acid–interacting proteins. This includes the method of stable-isotope labeling with amino acids in cell culture and affinity enrichment assays coupled with quantitative proteomics analysis^4–10^. However, these methods treat target nucleic acid as a bait, identify only proteins with high affinity toward the nucleic acid, and overlook weakly and transiently bound proteins. Proximity labeling (PL) approaches have been developed as an alternative to conventional methods. For instance, APEX-based mapping of RNA–protein interactions followed by phase separation (APEX-PS)^11^ has achieved great success. Additionally, APEX2 has been employed to identify mitochondrial G4 DNA-binding proteins^12^. Chemical labeling methods using photosensitive functional groups (e.g., diazirine, aryl azide, and benzophenone) or electrophiles (chloroacetamide, glyoxal, and tosyl groups) introduced onto nucleic acids circumvent the use of engineered fusion proteins and enable target protein tagging^13–18^. Recently, light-induced lysine (K)-enabled crosslinking (LIKE-XL) strategy allowed spatiotemporal and global profiling of DNA–protein interactions, including low-affinity protein interactors^19^.

G-quadruplexes (G4s) are four-stranded nucleic acid structures folded under physiological conditions within guanine (G)-rich regions; they comprise two or more stacked G-tetrads^20,21^. DNA G4s have been found to be enriched in several genomic regions, including telomeres and promoters of oncogenes^22,23^. Therefore, G4s play a critical role in essential biological processes, such as telomere elongation and DNA replication, repair, and transcription^24^. It was suggested that the functions of G4s are associated with their interacting proteins. Therefore, highly specific and accurate profiling methods for G4–protein interactions are required to unravel the underlying mechanisms of G4-interacting proteins (G4IPs)-related biological processes and molecular functions. Among the recent methods developed to identify G4IPs^25^, G4 ligand-mediated cross-linking and pull-down (G4-LIMCAP)^26^, and co-binding-mediated protein profiling (CMPP)^27^ coupled with mass spectrometry-based proteomics can detect G4IPs in living cells. These methods utilize a small molecule consisting of a G4 probe (pyridostatin derivative) bound with a photocrosslinker (diazirine group) to label the proteins in close proximity to the G4 structure upon irradiation. Although many unknown G4IPs have been discovered under real cellular conditions, it is highly concerning that the G4 probe may share the binding site on G4 with some proteins, preventing them from binding and further labeling. In addition, the diazirine reactive unit reacts equivalently with proteins and may lack the ability to provide a comprehensive analysis. Thus, novel techniques are required for discovering unrevealed G4IPs.

Photocatalytic PL is a powerful platform for selectively investigating protein–protein interactors. The MicroMap approach, where carbene reactive species are generated from diazirine via Dexter energy transfer, is one of the most successful PPL examples^28^. In another PPL method, the free labeling reagent 1-methyl-4-arylurazole (MAUra) is used for the selective PL of histidine^29,30^. The photocatalysts used in this method, such as Ru complex or BODIPY (BDP), can catalyze both single electron transfer (SET) and/or singlet oxygen (^1^O_2_) generation, so that the labeling reagent reacts with proteins around the photocatalyst upon irradiation with blue light. Indeed, Ru-containing functional oligonucleotides, such as aptamers, have recently been developed as targeted photosensitizing agents for photodynamic therapy^31^. In a previous study, the *N*-methyl luminol derivative and hemin in a peroxidase-PL approach were exploited for the labeling of RNA G4-binding proteins^32^. Other recent proximity labeling approaches have also targeted RNA G4s^33^ or utilized G4-binding peptides^34,35^.

**Fig. 1:**
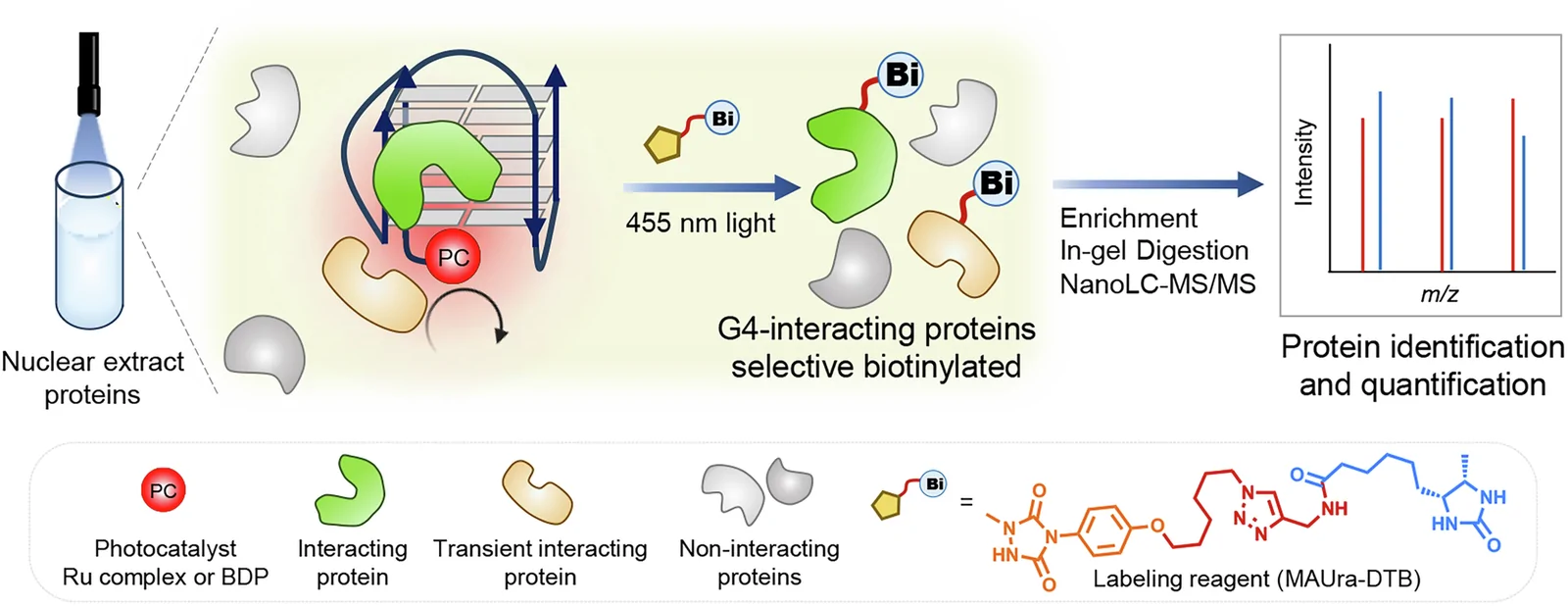
Schematic of our PPL approach. PPL concept for the profiling of DNA G4IPs using photocatalyst (Ru complex or BDP)-modified DNA–G4 with a free labeling reagent MAUra–DTB conjugate.

Based on the accumulated knowledge in the field and considering the previously reported photosensitizer-modified nucleic acids^36,37^, we developed a PPL approach for the investigation of DNA G4IPs. We modified a human telomere (HT) DNA G4 sequence with either a Ru complex or BDP as a photocatalyst. In this study, we systematically compared these two catalysts to evaluate how physicochemically distinct molecules—a bulky, cationic transition-metal complex versus a relatively compact, neutral organic fluorophore—behave when conjugated to specific local microenvironments of G4 DNA structures. A MAUra–desthiobiotin (DTB) conjugate was employed to efficiently and selectively capture proteins bound to the photocatalyst-modified DNA G4 (Fig. 1). Additionally, we successfully applied our optimized PPL approach to the global profiling of G4IPs in nuclear extracts.

## Results

### Molecular design and synthesis of photocatalyst-conjugated DNA

**Fig. 2:**
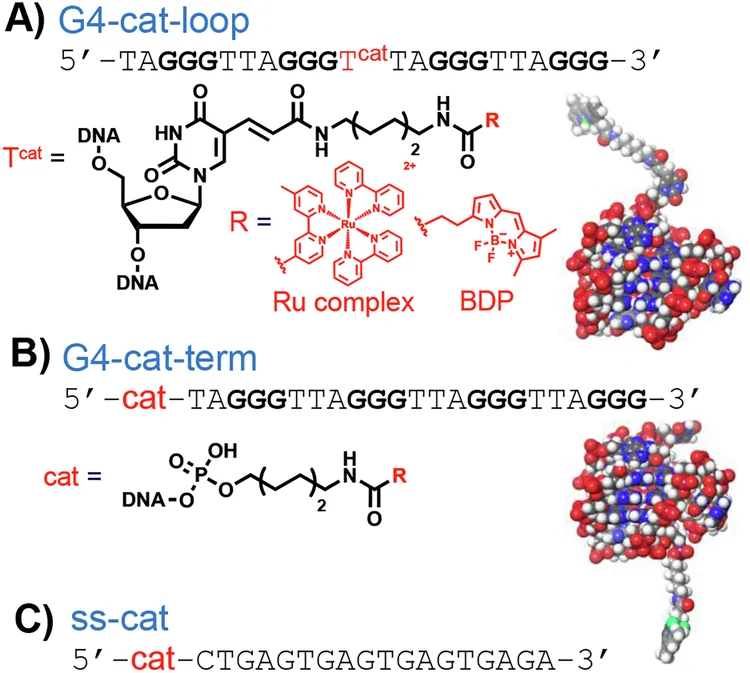
Molecular design of photocatalyst-conjugated DNA. The Ru complex or BDP was tethered to the* wt* Tel23 G4 sequence at **A** the internal loop and **B** the 5′-terminal. The HT G4 DNA–BDP structure was calculated using MacroModel with the OPLS3e force field based on the reported hybrid G4 structure [PDB: 2gku]^38^. **C** The non-G4-forming ssDNA conjugated with the photocatalyst. Photocatalyst conjugation was performed using DNA-NH_2_ (0.5 mM) and photocatalyst NHS ester (Ru complex = 12.5 mM and BDP = 10 mM) in 100 mM Na borate buffer (pH 8.7) at room temperature for 15 min in the dark.

For the molecular design of our photocatalyst-conjugated DNA probe, the HT sequence was deliberately chosen as the initial model system. The main reason for this choice is that the HT sequence is one of the most extensively characterized G4 structures to date. Because a substantial body of historical data is available on both its structural folding behaviors^38,39^ and its interacting proteome^7,8,40,41^, it serves as an ideal, data-rich benchmark. Using this well-established system allows us not only to assess the structural integrity of our modified probes but also to validate the biological fidelity of our newly developed PPL methodology by accurately identifying known G4-binding partners as internal positive controls. As cellular proteins can bind to the G4 structure through top-stacking, loop-binding, or groove-binding^40^, we first designed the modified HT G4 DNA sequence, *wt* Tel23, with a terminal amine-containing linker at the internal loop (G4-NH_2_-loop) and 5′-terminus (G4-NH_2_-term; Fig. 2A, B and Supplementary Scheme S1)^38^. The prepared sequences were then conjugated with a Ru complex or BDP via amide linkage using *N*-hydroxysuccinimide ester as a photocatalyst to obtain the corresponding photocatalyst-modified G4 DNA (G4-cat). To discriminate non-G4-specific binding proteins, NH_2_-modified ssDNA (ss-NH_2_) was prepared and conjugated with photocatalysts in the same manner to obtain photocatalyst-modified ssDNA (ss-cat; Fig. 2C). The molecular weights of the obtained cat-modified DNAs were confirmed with MALDI-TOF measurement (see experimental data).

Circular dichroism (CD) measurements for the native and photocatalyst-modified HT DNA G4 sequences were performed to verify the conformational structure after modification (Supplementary Fig. S1). The CD spectra of the G4-Ru term show two positive peaks at around 270 and 290 nm and a negative peak at around 237 nm; these peaks are the same as native G4, indicating the hybrid G4 structure in G4-Ru (Supplementary Fig. S1A)^42^. However, Ru-loop spectra show additional positive peaks at 320 and 247 nm. While the CD spectra of the G4-BDP-term confirm the presence of a hybrid structure, in the G4-BDP-loop spectra, the intensity of the peak at around 268 nm is higher, indicating a partial change to a parallel structure (Supplementary Fig. S1B)^42^.

To further examine possible conformational effects caused by chemical modification, native PAGE analysis was performed (Supplementary Fig. S2). The constructs G4-BDP-term, G4-Ru-term, and G4-Ru-loop migrated as sharp single bands, suggesting that their overall hybrid-type topology is preserved with minimal disturbance beyond the expected steric bulk of the catalysts. In contrast, the G4-BDP-loop showed a slightly broader band migration pattern. Considered together with its mixed CD profile, this broadening suggests that modification in the loop region slightly perturbs the original hybrid topology, resulting in a dynamic ensemble of G4 conformations under our assay conditions.

The thermal stability of the G4-photocatalyst was investigated via melting CD measurements (Supplementary Table S1 and Supplementary Fig. S3). The recorded *T*_m_ values of the Ru-loop (74 °C, ∆*T*_m_ = +3 °C) and Ru-terminal (80 °C, ∆*T*_m_ = +9 °C) are higher than those of the native G4 (71 °C), BDP-loop (69 °C, ∆*T*_m_ = −2 °C) and BDP-terminal (71 °C, ∆*T*_m_ = 0 °C), indicating that the Ru-modification increased the stability of G4.

### Photolabeling reaction of UP1

Next, we investigated the photolabeling reaction. Both Ru complex and BDP can catalyze singlet oxygen (^1^O_2_) generation upon irradiation with blue light, causing oxidization of nearby amino acids, e.g., histidine, producing highly reactive endoperoxides, which are trapped by the strong nucleophile MAUra (Supplementary Fig. S4)^29,30^. The histidine residues near the photocatalyst (Ru complex or BDP) were labeled because of the short lifetime^43^ and restricted diffusion radius^44^ of the generated singlet oxygen.

**Fig. 3:**
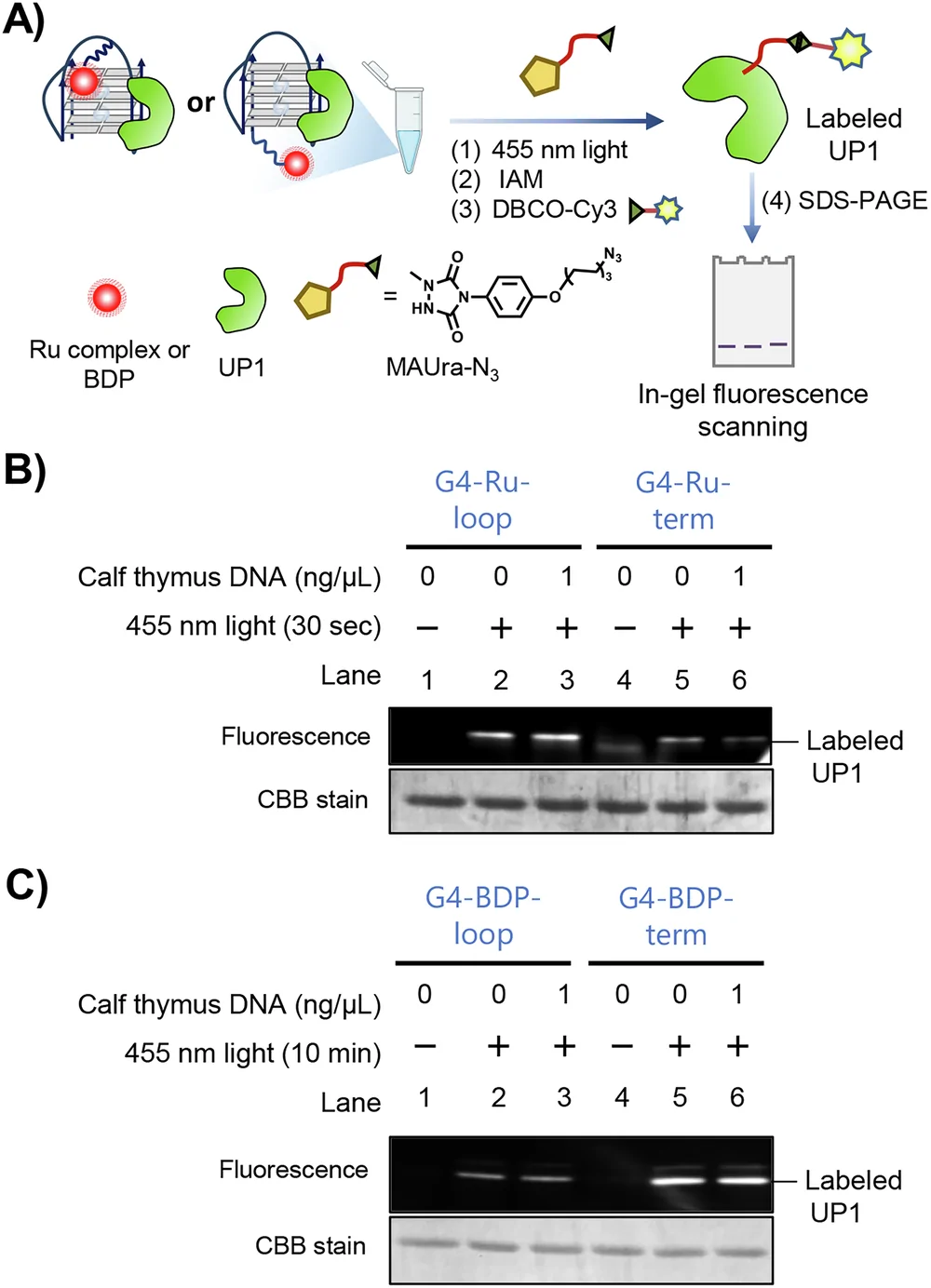
Photolabeling of UP1 using G4-cat-loop and G4-cat-term with MAUra-azide labeling reagent. **A** Schematic workflow of the labeling reaction. **B** Ru complex–mediated labeling reaction in the presence and absence of calf thymus DNA. **C** BDP-mediated labeling reaction in the presence and absence of calf thymus DNA. Reaction conditions were as follows: G4-cat [0.5 µM], MAUra-N_3_ [1.0 mM], UP1 [1.0 µM], and calf thymus DNA [1 ng/µL] in 50 mM Tris-HCl buffer (pH 7.5) containing KCl [100 mM], 455 nm LED light, 0 °C, 30 s (Ru complex), or 10 min (BDP).

Unwinding protein 1 (UP1) was employed as a model G4IP. UP1 is the proteolytic fragment of heterogeneous nuclear ribonucleoprotein (hnRNP) A1, which has been shown to bind to the HT sequence d(TTAGGG)_n_ in vitro and in vivo^39,45^. The Ru complex–mediated labeling reaction using G4-Ru was performed by photoirradiating the reaction mixture with 455 nm light for 30 s on ice in the presence of MAUra-N_3_ (Fig. 3A, B). The labeled UP1 with MAUra-N_3_ was conjugated with DBCO-Cy3 *via* a strain-promoted alkyne-azide cycloaddition (SPAAC) reaction. The results of SDS-PAGE analysis show that UP1 labeling is more efficient for the G4-Ru-loop (Fig. 3B, lane 2) than for the G4-Ru-terminal (Fig. 3B, lane 5). Moreover, the addition of calf thymus DNA did not affect the labeling efficiency (Fig. 3B, lanes 3 and 6), indicating the G4-specific binding of UP1. For the BDP labeling reaction, the mixture was photoirradiated for 10 min on ice under similar conditions (Fig. 3A, C). SDS-PAGE results indicate that UP1 labeling using the G4-BDP term (Fig. 3C, lanes 5 and 6) is more efficient than that using the G4-BDP loop (Fig. 3C, lanes 2 and 3). In the fluorescence spectra, BDP in the loop shows a 10-fold lower fluorescence intensity than that at the terminal (Supplementary Fig. S5A), suggesting that the labeling efficiency for the G4-BDP-loop was low because of BDP quenching caused by the guanine nucleotides of G4. Such a high quenching was not observed for the Ru complex in the loop (Supplementary Fig. S5B).

Labeling of UP1 with ss-cat was also investigated (Supplementary Fig. S6). The labeling efficiency for both ss-Ru and ss-BDP (lanes 3 and 6) is significantly lower than that for G4-Ru after the addition of calf thymus DNA, suggesting that UP1-specific binding to G4 is important for efficient labeling.

To confirm that the binding of UP1 to G4 is critical for efficient labeling, we used a mutant UP1, which cannot bind to G4 (Supplementary Fig. S7). As previously reported, R55 (in RRM1) and R146 (in RRM2) of UP1 interact with the guanine backbone of the TAGG sequence of HT DNA *via* electrostatic interactions with phosphate and hydrogen bonding with O5’, respectively^24^. The mutant UP1, in which these amino acids are replaced with A55 and A146, could not bind to the G4 structure (Supplementary Fig. S7A)^32^. The more efficient G4-Ru-loop and G4-BDP-term were used for the photolabeling reaction in subsequent experiments. As expected, for both G4-Ru-loop (Supplementary Fig. S7B, lane 4) and G4-BDP-term (Supplementary Fig. S7C, lane 4), mutant UP1 is less labeled than UP1 (lane 2), suggesting that the binding of UP1 to G4 enhanced the labeling efficiency.

**Fig. 4:**
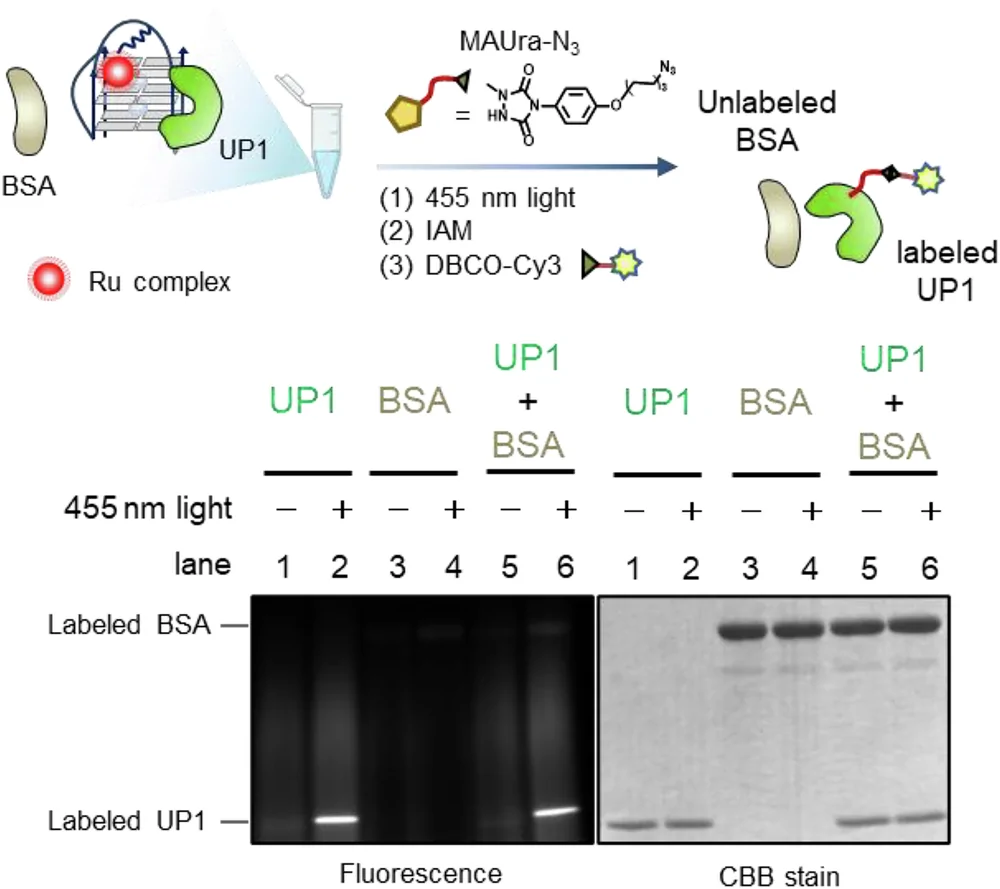
UP1 labeling in the presence of BSA using the G4-Ru loop. G4-Ru [0.5 µM], MAUra-N_3_ [1.0 mM], UP1 [1.0 µM], BSA [1.0 µM], and calf thymus DNA [1 ng/µL] in 50 mM Tris-HCl buffer (pH 7.5) containing KCl [100 mM]. The reaction mixture was photoirradiated with 455 nm LED Light for 30 s at 0 °C. The labeled protein was visualized using DBCO-Cy3.

We also verified the selectivity of G4-binding protein labeling on a mixture of bovine serum albumin (BSA) and UP1 (Fig. 4 and Supplementary Fig. S8). BSA did not bind to the G4-Ru-loop and was not labeled (Fig. 4, lane 4), whereas UP1 was labeled (Fig. 4, lane 6). UP1 was also selectively labeled when using the G4-BDP term (Supplementary Fig. S8, lane 6). These data indicate the high G4-binding protein selectivity of the proposed labeling approach.

Next, we determined the labeling site on UP1 using non-azide MAUra and nano LC-MS/MS (Supplementary Fig. S9 and S10). When using the G4-Ru loop (Supplementary Fig. S9A), mass spectrometry identified His 101, His 119, and His 173 as MAUra-labeled amino acid residues on UP1 (Supplementary Fig. S9B and S9C, and Supplementary Table S2). When using the G4-BDP term (Supplementary Fig. S10A), His 119 or 120 was mainly labeled (Supplementary Fig. S10B, S10C, and Supplementary Table S3). The solvent accessibility estimated^46^ for histidine residues revealed that His 101, 119, 120, and 173 are sufficiently accessible to the solvent (Supplementary Fig. S11). Hence, in addition to catalyst proximity by binding, high solvent accessibility is essential for the considered labeling reaction. As previously mentioned, the Ru complex can catalyze singlet oxygen generation, enabling the labeling of histidine. To confirm that singlet oxygen (^1^O_2_) is the predominant reactive species in MAUra-based PL, we performed a quenching experiment using sodium azide (NaN_3_), a well-established ^1^O_2_ quencher. UP1 was incubated with each photocatalyst-modified G4 construct and MAUra-N_3_ in the presence or absence of 10 mM NaN_3_, followed by standard photoirradiation at 455 nm. Subsequent SDS-PAGE analysis revealed that fluorescence labeling of UP1 was substantially reduced ( > 90% reduction) in the presence of NaN_3_ across all constructs tested (Supplementary Fig. S12). This strong suppression provides compelling experimental evidence that the photolabeling reaction in our system is predominantly mediated by the singlet oxygen pathway.

The G4 self-labeling and self-damage in the absence of UP1 under labeling reaction conditions were investigated (Supplementary Fig. S13A)^47,48^. The photoreaction was followed by conjugation with DBCO-PEG_4_-NH_2_ *via* the SPAAC reaction to separate the labeled G4-cat in gel, followed by PAGE analysis. For G4-Ru, G4 was intact in the absence of MAUra-N_3_ (Supplementary Fig. S13B, lanes 2). At the same time, in the presence of MAUra-N_3_ with or without calf thymus DNA, labeled G4 was not observed (Supplementary Fig. S13B, lanes 3 and 4). For the G4-BDP term, although a slightly faster-migrating band suggesting minor photodegradation was observed upon photoirradiation, the vast majority of the G4 probe remained intact (Supplementary Fig. S13C right, lanes 2–4). Additionally, negligible G4 labeling was observed in the presence of MAUra-N_3_ with or without calf thymus DNA (Supplementary Fig. S13C right, lanes 3 and 4). Notably, in a previous report, where the photolabeling of the DNA target was conducted with non-methyl 4-arylurazole, DNA was sufficiently labeled^47^. Hence, the photolabeling of G4 with MAUra was suppressed by the methyl group of MAUra. For the G4-BDP-loop, significant BDP photobleaching upon irradiation was observed (Supplementary Fig. S13C left, lanes 2–4). The rigid linker in the G4-BDP-loop facilitated BDP quenching and photobleaching by the near-G4 guanines, resulting in the low photolabeling efficiency of UP1 in Fig. 3C.

We also evaluated the structural and functional integrity of the probes before and after photoirradiation. CD spectroscopy showed that although a slight decrease in overall ellipticity was observed after standard irradiation, the characteristic G4 spectral profiles and peak positions remained essentially unchanged for all constructs (Supplementary Fig. S14). This indicates that the overall G4 topologies are preserved. Furthermore, EMSA confirmed that the binding affinity of UP1 for the irradiated G4 probes was maintained, with band-shift patterns comparable to those of the non-irradiated controls (Supplementary Fig. S15). Taken together, these validations confirm that both the G4 topologies and their protein-binding capabilities are adequately preserved during the labeling reaction, ensuring the reliability of our interactome analysis.

### Global profiling of G4 DNA-interacting proteins using PPL

Next, we evaluated our photocatalytic labeling approach on nuclear extract proteins of HEK293FT cells using free catalyst (cat), ss-cat, ds-cat, or G4-cat (Supplementary Fig. S16A, B). The labeling reaction was conducted in the presence of MAUra-desthiobiotin (DTB) to capture the labeled proteins. The photoirradiation of the reaction mixture was followed by western blotting (Supplementary Fig. S16A). Sufficient labeled proteins were observed for all experiments involving the Ru complex without significant differences in the profile (Supplementary Fig. S16C). In the case of BDP (Supplementary Fig. S16D), whereas no labeled proteins were observed in the absence of the photocatalyst (Supplementary Fig. S16D, lane 1), the labeled proteins were clearly observed for the free BDP, ss-BDP, and G4-BDP-term (Supplementary Fig. S16D, lanes 2, 3, and 6, respectively). In contrast, the labeled proteins were not observed for ds-BDP with 3’ terminal guanine (*w/* G) and G4-BDP-loop (Supplementary Fig. S16D, lanes 4 and 7, respectively) due to the quenching of BDP by the nearby guanines, as previously described. In the case of ds-BDP, where the 3’ terminal guanine base was removed from the complementary strand, labeled proteins were clearly observed (Supplementary Fig. S16D lane 5). These data suggest that the labeling reaction was successfully conducted for both Ru and BDP, resulting in a sufficient amount of labeled proteins for the proteomics analysis. However, western blotting did not show significant profile differences among G4-cat, dsDNA-cat, and free cat, probably because of the inability of western blotting to visualize large numbers of labeled proteins. Owing to its severe quenching and conformational heterogeneity, G4-BDP-loop was excluded from the mass spectrometry-based global proteomics analysis to ensure the integrity of our overall interactome datasets.

**Fig. 5:**
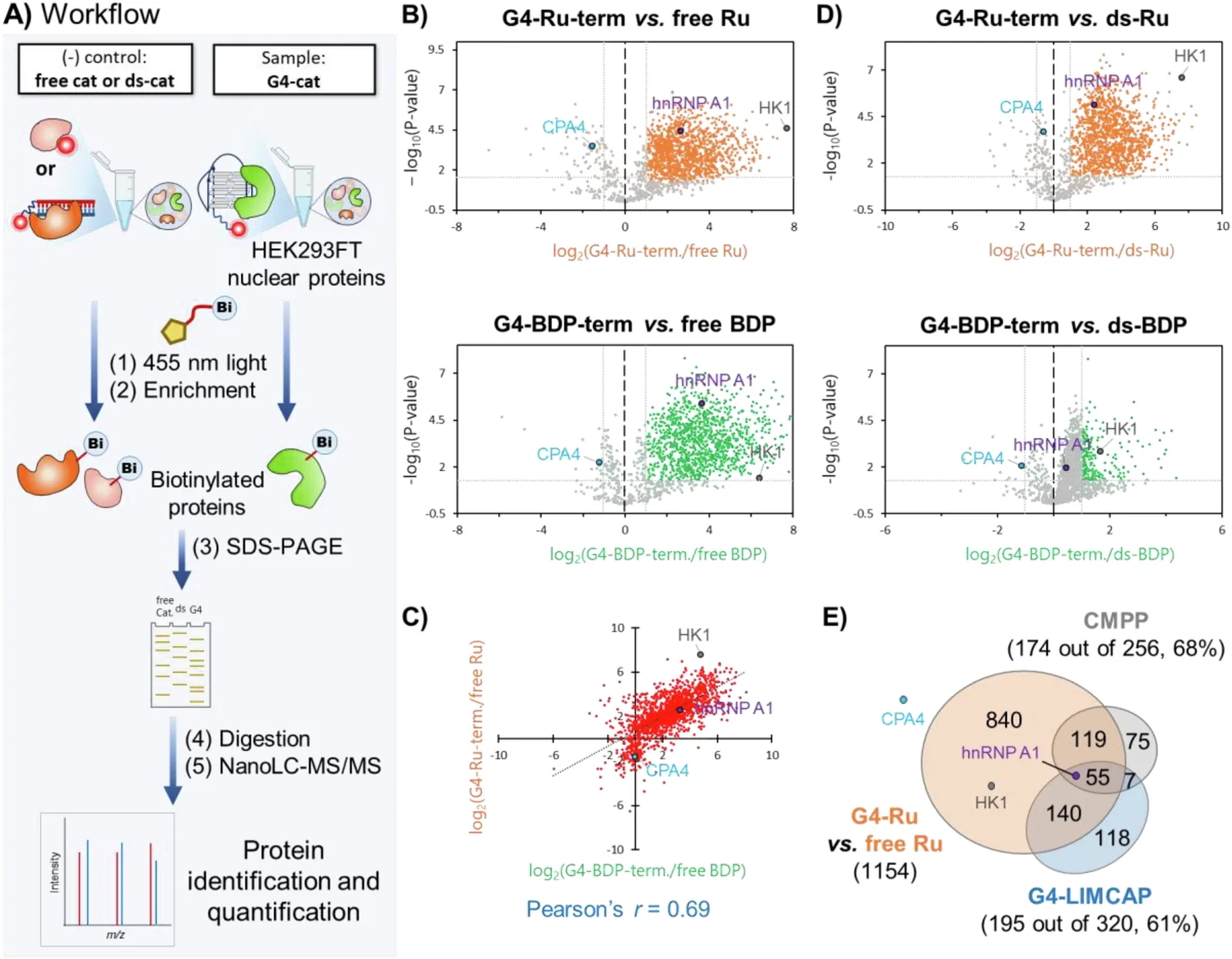
Profiling of biotinylated DNA G4IPs via LC-MS/MS-based proteomics analysis. **A** Schematic workflow of profiling G4IPs in HEK293FT nuclear proteins using G4-cat-term, with free cat or ds-cat used as controls. **B** Volcano plot showing enriched proteins (highlighted in orange) for G4-Ru-term versus free Ru (*n* = 1154 out of 1584; above) and enriched proteins (highlighted in green) for G4-BDP-term versus free BDP (*n* = 1197 out of 1573; below). **C** The correlation between G4-BDP-term and G4-Ru-term proteins versus free BDP and free Ru, respectively. **D** Volcano plot showing enriched proteins (highlighted in orange) for G4-Ru-term versus ds-Ru (*n* = 1187 out of 1584; above) and enriched proteins (highlighted in green) for G4-BDP-term versus ds-BDP (*n* = 210 out of 1584; below). Proteins were considered enriched with a more than 2-fold stronger signal than the control and a *P*-value < 0.05. **E** Venn diagram showing the overlap between enriched proteins for G4-Ru-term vs. free Ru compared to G4-LIMCAP^26^ and CMPP^27^ methods. In **B**–**E**, hnRNP A1, a known G4IP, CPA4 (negative control protein candidate), and HK1 (unknown G4IP candidate) are marked in purple, blue, and gray, respectively.

Subsequently, we identified the biotinylated proteins using MAUra-DTB and a quantitative nano LC-MS/MS proteomics approach (Fig. 5). In this experiment, we identified G4IPs using the G4-Ru or G4-BDP terms. In the control experiment, we used free cat and ds-cat to factor out possible off-target proteins. After photoirradiation under our standard conditions in the presence of MAUra-DTB, the biotinylated proteins were affinity-purified on streptavidin-conjugated beads, followed by cutting out gels of full molecular weight, in-gel digestion, and quantitative nano LC-MS/MS analysis (Fig. 5A). The data are summarized in Supplementary Data 1 (for G4-Ru) and Supplementary Data 2 (for G4-BDP). The significantly enriched proteins in the G4-cat experiment over free cat (Fig. 5B) or ds-cat (Fig. 5D; fold change > 2, *P*-value < 0.05) were deemed G4IP candidates. For free cat as a control (Fig. 5B), the volcano plot visualizing the proteomics data shows numerous G4IP candidates that are significantly enriched for both G4-Ru-term (*n* = 1154 out of 1584) and G4-BDP-term (*n* = 1197 out of 1573) over the free Ru and free BDP, respectively (Fig. 5B). The G4-Ru-term and G4-BDP-term proteins are highly correlated (Pearson’s *r* = 0.69; Fig. 5C). Moreover, enriched proteins for G4-Ru-term share around 90% of species (1034 out of 1154) with enriched protein candidates for G4-BDP-term versus free cat (Supplementary Fig. S17A), suggesting that the effects of the photocatalysts utilized in this labeling approach are not significantly different. When using ds-cat as a control (Fig. 5D), G4IP candidates are significantly enriched for G4-Ru-term (*n* = 1187 out of 1584) over ds-Ru and share around 91% of species (1084 out of 1187; Supplementary Fig. S17B) with enriched protein candidates for G4-Ru-term versus free Ru (Fig. 5B, above). However, the G4IP candidates that are significantly enriched for G4-BDP-term (*n* = 210 out of 1584) over ds-BDP (Fig. 5D, below) are considerably fewer than protein candidates that are enriched over free BDP, suggesting that BDP quenching with different efficiencies between G4-BDP-term and ds-BDP due to differences in the catalyst environment affected the proteomics accuracy. Although in previous reports, the results of G4-LIMCAP and CMPP barely overlapped, some of the G4IP candidates, particularly those enriched for G4-Ru-term over free Ru, overlapped with most of the G4IPs identified using G4-LIMCAP^26^ (195 out of 320, 61%) and CMPP^27^ (174 out of 256, 68%; Fig. 5E), providing another support for our method. Some of the G4IP candidates enriched for G4-Ru term over free Ru partially overlapped with those in the G4IPDB (29/94, 30%), a database of G4IPs compiled from the literature^41^. This 30% overlap is comparable to the 24% overlap previously observed with CMPP, independently supporting the validity of our method. Notably, several well-known G4IP proteins were successfully identified among these candidates, such as hnRNP A1 (marked in purple in Fig. 5B–E). Conversely, carboxypeptidase A4 (CPA4) (marked in blue in Fig. 5B–E) was not enriched in G4-Ru-term vs. the controls and was therefore selected as a negative control for the subsequent biochemical assays.

Among the newly identified G4IP candidates, we focused particularly on hexokinase-1 (HK1) (highlighted in gray in Fig. 5B–E), which ranked first for G4-Ru-term vs. free Ru and sixth for G4-Ru-term vs. ds-Ru. To the best of our knowledge, no direct interactions between the HK1 protein and G4 structures have been reported. HK1 is localized at the outer mitochondrial membrane and plays a pivotal role in cellular glucose uptake and energy production by catalyzing the first rate-limiting step of glucose metabolism^49,50^. In addition, HK1 exhibits anti-apoptotic activity by antagonizing Bcl-2 proteins^51^ and plays a critical role as a cell survival and oxidative damage protector^50,52^. These functions sparked our interest because they differ from common G4IPs-related functions mentioned above. To gain deeper biological insight and assess the functional connectivity of these identified candidates, we performed STRING network^53^ and Gene Ontology (GO) enrichment^54^ analyses on the proteins identified by the terminal-modified probes (Supplementary Fig. S18 and Supplementary Data 3). The GO analysis revealed significant enrichment in biological processes closely associated with established G4 functions, forming highly interconnected clusters in RNA splicing, DNA replication, and chromatin organization. Notably, the topological distribution of specific proteins within this network is informative regarding both the validity and the novelty of our approach. On one hand, the well-known G4-binding protein hnRNP A1 is integrated into the center of the major RNA splicing cluster, serving as an internal benchmark that supports the biological relevance of our dataset. On the other hand, consistent with its unique functional background described above, HK1 stands clearly apart from these major, classical macromolecular complexes. Because STRING reflects existing annotations, this separation highlights a lack of previously reported functional links. This lack of prior annotation made HK1 a highly attractive candidate, providing strong motivation to directly test its G4 binding. Overall, although PL inherently captures broader molecular environments, the ability of our PPL method to surface such candidates outside the classical clusters highlights its potential as a discovery tool for expanding the known landscape of G4 biology.

We aimed to identify the G4IP candidates captured with the Ru complex modified at the loop position (G4-Ru-loop) via proteomics analysis (Supplementary Fig. S19). Few G4IP candidates were enriched for G4-Ru-loop versus free Ru (*n* = 41 out of 1583 proteins, Supplementary Fig. S19A) and ds-Ru (*n* = 37 out of 1573 proteins, Supplementary Fig. S19B). The proteins captured for the G4-Ru-loop versus both free Ru and ds-Ru do not correlate with proteins captured for the G4-Ru-term, suggesting that the modification with the Ru complex at different sites on the G4 structure led to the identification of different proteins, expanding the protein identification capabilities of the method (Supplementary Fig. S19C and S19D). However, several known G4IPs, including hnRNP A1 and HK1, were not enriched among the captured proteins with the loop-modified Ru (Supplementary Fig. S19) probably because modifications in the loop reduced G4 binding kinetics toward these proteins within this complex system. This distinct capture profile, which depends on the modification site, illustrates how local steric environments determine the accessible interactome, highlighting the critical importance of site flexibility. Existing ligand-based PL methods have considerably advanced the mapping of G4 interactomes. However, because these small-molecule ligands inherently bind to specific G4 pockets, they can sterically displace certain native interactors, a phenomenon conceptually similar to the specific masking effect observed with our loop modification toward HK1 and hnRNP A1. Our PPL approach overcomes this established limitation through its unique site flexibility. Instead of relying on a single binding pocket, our PPL enables the strategic placement of the photocatalyst (e.g., terminal vs loop). While the terminal probe successfully captured HK1 and hnRNP A1, the loop probe enriched a complementary set of candidates. By combining these site-specific profiles, our PPL reduces the risk of overlooking interactors owing to local microenvironmental constraints. Importantly, the substantial overlap in proteins identified by our PPL method and previous ligand-based approaches supports the validity of both strategies. Thus, our site-flexible PPL method provides a versatile and complementary toolkit, broadening the technical repertoire for broadly mapping complex nucleic acid interactomes without predefined spatial constraints.

### Characterization of the G4IP candidate proteins

**Fig. 6:**
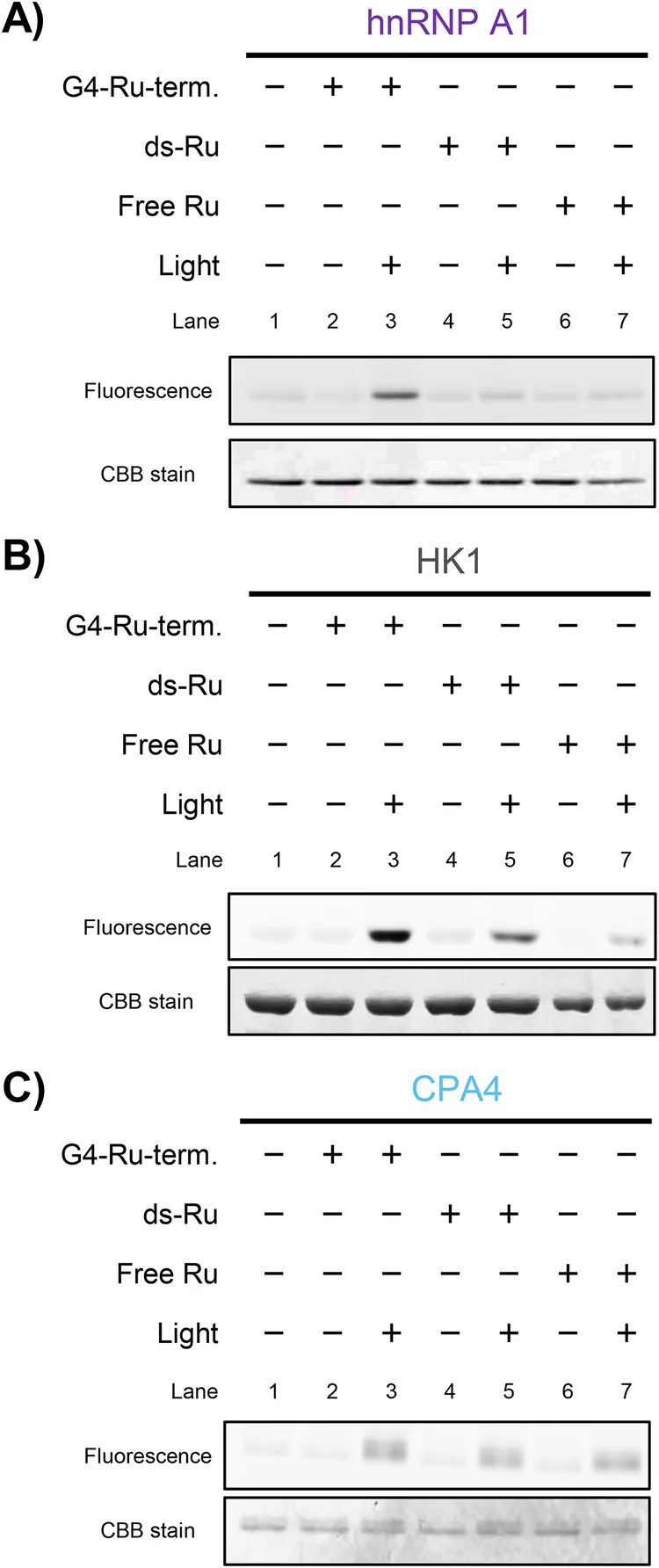
Validation of the labeling method using SDS-PAGE. **A** hnRNP A1, **B** HK1, and **C** CPA4 (1.0 µM) were photolabeled using G4-Ru term, ds-Ru, and free Ru. The DNA-cat and free cat were added at 2.0 µM, and the reaction mixture was photoirradiated with 455 nm LED light for 30 s in the presence of MAUra-N_3_ [1.0 mM] and calf thymus DNA [1 ng/µL] in 50 mM Tris-HCl buffer (pH 7.5) containing KCl [100 mM]. The labeled proteins were visualized via in-gel fluorescence scanning and stained with CBB stain.

**Fig. 7:**
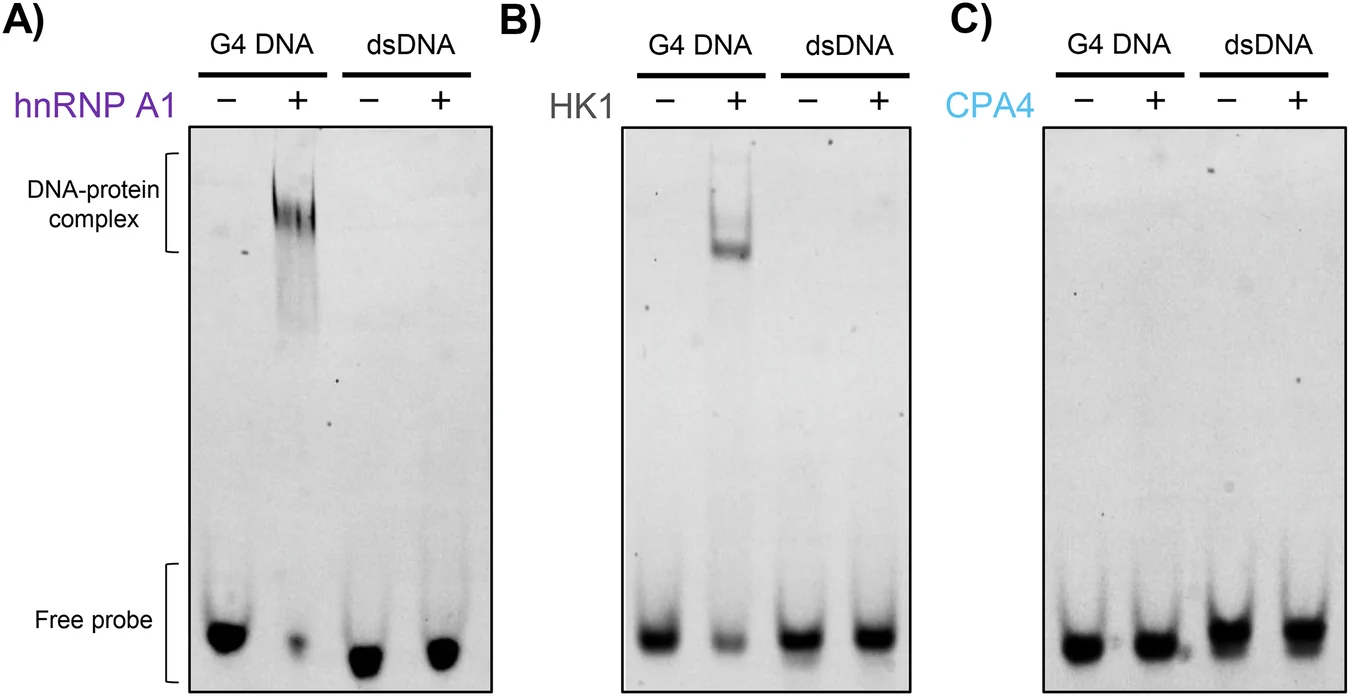
Validation using EMSA. The binding of **A** hnRNP A1 (1.0 µM), **B** HK1 (1.0 µM), and **C** CPA4 (1.0 µM) with FAM-labeled G4 DNA (5.0 nM) and dsDNA (5.0 nM) sequences was determined in the presence of calf thymus DNA (1 ng/µL).

Finally, the photolabeling and binding properties of the newly identified G4IP candidate, HK1, were characterized alongside those of the positive control (hnRNP A1) and negative control (CPA4). The photolabeling of these three proteins was conducted under our standard conditions using G4-Ru-term, ds-Ru, and free Ru (Fig. 6). Both hnRNP A1 and HK1 proteins were efficiently labeled with G4-Ru-term (Figs. 6A, B, lane 3) and could not be efficiently labeled using ds-Ru and free Ru (Figs. 6A, B, lanes 5, 7 respectively), indicating the efficiency of our labeling approach. On the other hand, CPA4 was non-selectively labeled using the G4-Ru term, ds-Ru, or free Ru, as expected (Fig. 6C). The binding affinities of the three proteins toward FAM-labeled G4 DNA and dsDNA sequences (same sequences as those used in the labeling approach) were investigated by electrophoretic mobility shift assay (EMSA; Fig. 7). Both hnRNP A1 and HK1 selectively formed complexes with G4 DNA (Figs. 7A, B). On the other hand, CPA4 did not form a complex with G4 DNA or dsDNA, as expected (Fig. 7C). The apparent dissociation constant (*K*_dapp_) value for HK1 determined using EMSA with the FAM-labeled G4 DNA sequence was 320 ± 49 nM (Supplementary Fig. S20). Although EMSA clearly demonstrated the selective binding of HK1 to G4 structures, we performed microscale thermophoresis (MST) measurements to obtain a quantitative dissociation constant under solution-phase equilibrium conditions. The MST-derived dissociation constant (*K*_d_) for the interaction of HK1 with Texas Red-labeled HT G4 DNA (20 nM, in 10 mM Tris-HCl (pH 7.5) containing 20 mM KCl and 0.05% Tween-20) was determined to be 11.8 ± 2.0 nM (*n* = 3; Supplementary Fig. S21). It should be noted that this MST-derived affinity is substantially stronger than the apparent *K*_d_ estimated from EMSA (320 ± 49 nM). This discrepancy reflects methodological differences: EMSA is a non-equilibrium assay in which complex dissociation during electrophoresis often leads to an underestimation of affinity, whereas MST measures the interaction in free solution under true equilibrium conditions. Additionally, the higher KCl concentration used in the EMSA buffer (100 mM) relative to the MST assay buffer (20 mM) is expected to attenuate electrostatic protein–DNA interactions, contributing to the weaker apparent affinity observed by EMSA. Data points at or above 400 nM ( ≥ 400 nM) HK1 were excluded from the fitting owing to fluorescence quenching of Texas Red by HK1 at high protein concentrations, as evidenced by a consistent decrease in both the capillary fluorescence intensity and Fnorm values; the raw MST traces and capillary fluorescence data are provided in Supplementary Fig. S21 to allow independent assessment of data quality and fitting robustness. These combined results confirm a high-affinity, specific interaction between HK1 and the G4 structure in solution, consistent with our PPL proteomics results. Furthermore, to assess the binding properties of the HK1 protein across different G4 topologies, we selected additional G4 oligonucleotides that can form different G4 structures (Supplementary Fig. S22). We conducted EMSA using Myc and NRAS G4 DNA sequences and TERRA, KRAS, and NRAS G4 RNA sequences. HK1 bound to both DNA and RNA G4s, showing the most prominent shift for NRAS G4 RNA. We note that this EMSA-based topology preference is a qualitative trend, and defining its quantitative basis is a natural next step opened up by these results.

## Conclusions

Herein, we developed a PPL approach to detect DNA G4IPs. In this method, the photocatalyst was covalently bound to G4, mediating the labeling of the interacting proteins with MAUra. Using this method, the G4-binding protein UP1 was efficiently and selectively labeled in a mixture of UP1 and BSA (Fig. 4). Moreover, mutant UP1 was less labeled, indicating the high selectivity of the proposed labeling approach (Supplementary Fig. S7). Labeling mainly occurred on histidine residues (Supplementary Fig. S9–S11).

The application of this approach to nuclear extract proteins resulted in plentiful G4-binding protein candidates being significantly enriched for G4-cat over the controls, free cat, or ds-cat (Fig. 5). By systematically comparing distinct photocatalysts (a Ru complex and BDP) in this profiling, we showed that their interactome profiles are strongly correlated, confirming that our PPL approach is free of catalyst-dependent bias. However, because BDP is susceptible to intramolecular quenching by nearby guanine bases, the Ru complex is the optimal choice for this profiling.

The utility of our PPL proteomics approach as a screening platform is supported by both internal and external benchmarks. Internally, the well-organized functional clustering of classical G4 machinery and the successful capture of the positive control (hnRNP A1) provide system-level support. Externally, our identified interactome showed meaningful overlap with candidate profiles mapped by established milestone approaches (e.g., G4-LIMCAP and CMPP), and several of our identified candidates were independently validated in a recent study^10^, strongly supporting the validity of our methodology. Furthermore, although singlet oxygen can diffuse up to ~220 nm in pure water^55^, abundant endogenous quenchers in the nuclear extract limit its effective labeling radius to a local macromolecular scale (tens of nanometers). Within this microenvironment, our PPL approach captures both direct G4 binders and indirect complex members. Rather than being a limitation, this capability offers a distinct advantage by enabling broader mapping of the functional, higher-order interaction networks that dynamically assemble around G4 scaffolds and are often lost in conventional direct-binding assays. Nevertheless, we acknowledge that, while our PPL platform successfully generates high-confidence candidate lists, the specificity and global reliability of the resulting interactome remain to be established, and individual candidates will require further orthogonal validation in future studies.

Among the identified candidates, we strategically prioritized the rigorous in vitro validation of HK1. The successful confirmation of HK1—a novel interactor distinctly isolated from classical nuclear complexes—clearly underscores the unique screening capability and discovery potential of our platform. Indeed, HK1 binds selectively to a range of G4 DNA/RNA structures over dsDNA (EMSA, Fig. 7 and Supplementary Fig. S22), and binds HT G4 with high affinity (*K*_d_ = 11.8 nM by MST, Supplementary Fig. S21)—demonstrating that HK1 is a high-affinity G4-binding protein as quantified for HT G4 and providing a foundation for future studies on its G4 topology preferences.

Our findings regarding the G4 DNA/RNA–HK1 protein interactions would be helpful to reveal essential subcellular G4-mediated functions of HK1. Recently, KRAS4A was discovered to be a direct regulator of HK1 activity^56^. KRAS4A is one of the two proteins generated by the *KRAS* gene, the most frequently mutated oncogene^56^. G4 structures may also be involved in HK1 function regulation, or HK1 may regulate G4-related gene expression. Further studies are needed to clarify the biological relationship between HK1 and G4 structures. Another hexokinase isozyme, HK2, was recently reported to be localized in the nuclei of acute myeloid leukemia normal hematopoietic stem and progenitor cells and was found to maintain stemness^57^. Thus, the determination of the binding affinity of other isoforms of hexokinase and binding domains toward G4 structures is of substantial scientific interest. Furthermore, unknown and attractive proteins such as HK1 will be included in the list of candidate G4-binding proteins. This list will contribute to the discovery of novel G4IPs and the elucidation of their interactions.

We believe that our PPL approach is a promising tool for investigating higher order structural motifs of nucleic acid–protein interactions and functional nucleic acid–protein interactions.

## Methods

### General methods

The general chemicals were purchased from commercial suppliers (FUJIFILM Wako Pure Chemical, the Tokyo Chemical Industry, Kanto Chemical, Nacalai tesque, or Aldrich) and used without further purification unless mentioned. The target DNAs and RNAs were purchased from JBioS (Japan). The ^1^H NMR spectra (400 MHz) were recorded using a Bruker AVANCE III 400 spectrometer. The ^1^H NMR spectra (600 MHz) and ^13^C NMR spectra (150 MHz) were recorded using a Bruker AVANCE III 600 spectrometer. The high-resolution electrospray mass analysis was performed using a Bruker MicrOTOFQ II. HPLC purification was performed using a JASCO HPLC System (PU-4180, UV-4075, and CO-2067Plus). The MALDI-TOF MS measurements were performed using a Bruker Autoflex Speed instrument utilizing a 3-hydroxypicolinic acid/diammonium hydrogen citrate matrix.

### UP1 labeling for SDS-PAGE

G4-cat in 50 mM Tris-HCl buffer (pH 7.5) containing 100 mM KCl was annealed by incubation at 90 °C, followed by cooling to 4 °C at a rate of 0.5 °C/min. The labeling reaction was performed in a final volume of 30 μL in the Tris-HCl buffer. Purified proteins (final concentration 1.0 μM), calf thymus DNA (1 ng/μL), and MAUra-N_3_ (from 100 mM stock solution in dimethyl sulfoxide; final concentration, 1 mM) were mixed with photocatalyst-modified HT DNA (final concentration 0.5 μM) and then incubated for 30 min on ice. The reaction mixture was photo-irradiated on ice with a 455-nm LED light, DC8-12V 15 M Max, and 200-mA MAX. For Cys alkylation, 2-iodoacetamide (IAM, final concentration 10 mM) was added to the reaction mixture and incubated for 1 h at room temperature in a dark place. To visualize the labeled proteins, the reaction mixture was treated with DBCO-Cy3 (Sigma-Aldrich, from 1.0 mM stock solution in DMF; final concentration, 100 μM) and incubated for 1 h at 37 °C. After the reactions, the labeled proteins were separated by sodium dodecyl sulfate polyacrylamide gel electrophoresis (SDS-PAGE) with 12% gels. A fluorescence image was obtained using a Molecular Imager PharosFX Plus (Bio-Rad Laboratories, Inc.). The gel was visualized with Coomassie brilliant blue (CBB) stain.

### UP1 labeling for identification of the labeling site

G4-cat in 50 mM Tris-HCl buffer (pH 7.5) containing 100 mM KCl was annealed by incubation at 90 °C, followed by cooling to 4 °C at a rate of 0.5 °C/min. The labeling reaction was performed in Tris-HCl buffer in a final volume of 60 μL (10 identical reactions were prepared). Purified proteins (final concentration 1.0 μM), calf thymus DNA (1 ng/μL), and non-azide MAUra (from 100 mM stock solution in dimethyl sulfoxide; final concentration, 1 mM) were mixed with G4-cat (final concentration 0.5 μM) and then incubated for 30 min on ice. In the case of G4-Ru-loop, the reaction mixture was photo-irradiated on ice for 30 sec with a 455 nm LED light, DC8-12V 15 M Max, and 200 mA MAX. For the G4-BDP term, the photoirradiation time was 10 min. The labeled proteins were separated by SDS-PAGE with 4–20% gels, and the gel pieces were transferred into microtubes, and water (1 mL) was added and incubated at 37 °C for 10 min. The solution was removed, and the washing procedure was repeated three times. For destaining, 50% CH_3_CN in 100 mM Tris buffer (pH = 8.0) was added and incubated at 37 °C for 10 min, and the solution was removed. CH_3_CN was added to the tubes for dehydration and then incubated at 37 °C for 10 min. Dithiothreitol (100 mM) in 100 mM Tris buffer (pH = 8.0) was added to the tubes for Cys reduction, incubated at 37 °C for 30 min, and then the solution was removed. 2-Iodoacetamide (250 mM) in 100 mM Tris buffer (pH = 8.0) was added to the tubes for Cys alkylation. The tubes were incubated at room temperature for 30 min in the dark, and the supernatant was discarded. The gels were washed with 100 mM Tris buffer (pH = 8.0) and 50% CH_3_CN in 100 mM Tris buffer (pH = 8.0). CH_3_CN was added to the tubes for dehydration. The tubes were incubated at 37°C for 10 min, and the supernatant was discarded. After the supernatant was discarded, trypsin solution was added to each tube and incubated overnight at 37 °C. The reaction was quenched by adding aqueous trifluoroacetic acid solution (final concentration: 0.4% v/v) and desalted using C18 pipette tips. The desalted solution was concentrated under reduced pressure, dissolved in 5% CH_3_CN/0.1% TFA, and applied for LC-MS/MS analysis.

### NanoLC-MS/MS for the identification of the labeled site on UP1

NanoLC-MS/MS analysis was performed using an LC-nano-ESI-MS comprising a quadrupole time-of-flight mass spectrometer (Triple TOF® 5600 system; SCIEX, Framingham, MA, USA) equipped with a nanospray ion source and nanoLC system (Eksigent Nano LC Ultra 1D Plus; SCIEX). The trap column used for nanoLC was a NanoLC Trap ChromXP C18, 3 μm 120 Å (SCIEX), and the separation column was a 12.5 cm × 75 μm capillary column packed with 3 μm C18-silica particles (Nikkyo Technos Co., Ltd., Tokyo, Japan). The micropump (flow rate: 300 nL/min) gradient method was used as follows: mobile phase A: 2% acetonitrile, 0.1% formic acid; mobile phase B: 80% acetonitrile, 0.1% formic acid aq. 0–20 min: 5–45% B, 20−21 min: 45–100% B, 21–26 min: 100% B. NanoLC-MS/MS data were acquired in an information-dependent acquisition mode controlled by Analyst^®^ TF 1.5.1 software (SCIEX). The settings for the data-dependent acquisition were as follows: accumulation time, 0.25 s; full MS (MS1, TOF-MS) scan range, 400–1250 *m/z*, excluding the former target ion for 12 s; and mass tolerance, 50 mDa. The top 10 signals per full MS scan were selected from MS2 scanning. The MS2 (product ion) scan accumulation time and range were 0.05 s and 100–1500 *m/z*, respectively. All experiments were conducted in triplicate. MS/MS spectra were searched against the respective amino acid sequences using MaxQuant (Freeware) with default settings. A FASTA file corresponding to UP1 sequence was used. For labeling, oxidation (+O) of His, Met, and Tyr residues, acetylation (+C_2_H_2_O) at the N-terminus, an adduct of MAUra (+C_9_H_7_N_3_O_2_; +189.054 Da) for Tyr residues, and an adduct of MAUra (+C_9_H_7_N_3_O_3_; +205.049 Da) for His residues were set as possible modifications.

### DNA self-damage and self-labeling investigations (PAGE analysis)

The reaction mixture consisted of G4-cat (5.0 µM), MAUra-N_3_ (0.5 mM), and calf thymus DNA (1 ng/μL) in 50 mM Tris-HCl buffer (pH 7.5) containing KCl (100 mM) in a total volume of 30 µL was irradiated with 455 nm LED light for 30 sec (Ru complex) or 10 min (BDP) at 0 °C. Some samples were irradiated in the absence of MAUra-N_3_ and/or calf thymus DNA. The samples were then incubated with DBCO-PEG_4_-NH_2_ (5 mM, final conc. 1.0 mM) at 37 °C for 3 h. All aliquots were then quenched by adding an equal volume of loading buffer (2×, 95% formamide, 50 mM EDTA pH 8.0). PAGE was performed by denaturing 16% polyacrylamide and 10% formamide gel electrophoresis with 1× TBE and 7.5 M urea at 250 V for 60 min. The gel was visualized using FLA-5100 (Fujifilm Co., Tokyo, Japan). The gel was then stained with SYBR gold, and the ODNs were visualized with FLA-5100 again.

### Nuclear extract proteins labeling

HEK293FT cells were maintained in Roswell Park Memorial Institute medium supplemented with 10% fetal bovine serum and 1% Penicillin-Streptomycin. For assays, cells were cultured in 6-well plates. The cells were suspended in K-Ca buffer (50 mM Tris-HCl (pH 7.5), 100 mM KCl, 2.5 mM CaCl_2_). The nuclear fraction was prepared using EzSubcell Extract (ATTO Corporation) according to the manufacturer's instructions. The nuclear fraction was lysed by sonication (model UR-20P, Tomy Seiko) and centrifuged at 16,200 × *g* for 10 min at 4 °C. The lysate concentrations were determined using a BCA Protein Assay Kit (Thermo Scientific). The labeling reaction was performed in a final volume of 100 μL in Tris-HCl buffer. Nuclear extract (final concentration, 1.0 mg/mL), MAUra-DTB (from 30 mM stock solution in dimethyl sulfoxide; final concentration, 0.5 mM), and calf thymus DNA (1 ng/μL) were mixed with photocatalyst-modified HT DNA (final concentration 5 μM) on ice. In the case of G4-Ru-loop, the reaction mixture was photo-irradiated on ice for 1 min with a 455 nm LED light, DC8-12V 15 M Max, and 200 mA MAX. For the G4-BDP term, the photoirradiation time was 10 min. The unreacted labeling reagent was polished up by MeOH/CHCl_3_ precipitation.

### Gel-based analysis of desthiobiotin-labeled nuclear extract proteins by western blotting

The labeled proteins were separated by SDS-PAGE with 12% gels, transferred to a 0.2 μm polyvinylidene difluoride (PVDF) membrane (Bio-Rad Laboratories, Inc.) using Trans-Blot Turbo (Bio-Rad Laboratories), blocked with Bullet Blocking One for Western Blotting (nacalai tesque), a blot was treated with Streptavidin-Peroxidase Polymer, Ultrasensitive (Sigma-Aldrich^®^), treated with Chem-Lumi One L (nacalai tesque), and chemical luminescence was detected with a Molecular Imager Fusion Solo S (VILBER LOURMAT).

### NanoLC-MS/MS sample preparation

The labeled nuclear extract was dissolved in 50 µL of PTS 1× (100 mM Tris-HCl, pH 8.0, 12 mM SDC, and 12 mM SLS), incubated at 95°C for 10 min, and mixed with 200 µL of 100 mM Tris-HCl, pH 8.0. Dynabeads™ MyOne™ Streptavidin C1 (Invitrogen) (0.5 mg) were added to the solution and shaken at 4 °C for 3 h. The beads were washed with PTS 1× (250 µL) three times, 20 mM HEPES (pH = 7.5) containing 100 mM NaCl and 1 M urea (250 µL) three times, and 20 mM HEPES (pH = 7.5) containing 1 M NaCl (250 µL) three times to remove the non-labeled proteins. 1× NuPAGE sample buffer (2 mM biotin and 200 mM DTT) was added to the magnetic beads-containing solution and incubated at 95 °C for 5 min to release the labeled proteins from the beads. For in-gel trypsin digestion, first, the labeled proteins were analyzed by SDS-PAGE with 14% gels, and the bands of labeled proteins were excised from the gel and cut into small pieces (ca. 1 mm pieces). The gel pieces were washed by incubation with ddH_2_O (1 mL) at 37 °C for 10 min. This step was repeated three times, and then the solution was discarded. The gel was de-stained by incubation with 50% CH_3_CN in 100 mM Tris-HCl pH 8.0 at 37 °C for 10 min, and then the solution was discarded. CH_3_CN was added to the tubes for dehydration, followed by incubation at 37 °C for 10 min; then, the solution was discarded. Dithiothreitol (100 mM) in 100 mM Tris-HCl pH 8.0 was added to the tubes for Cys reduction, incubated at 37 °C for 30 min, and then the solution was removed. 2-Iodoacetamide (250 mM) in 100 mM Tris-HCl pH 8.0 was added to the tubes for Cys alkylation. The tubes were incubated at room temperature for 30 min in the dark, and then the solution was removed. The gels were washed with 100 mM Tris-HCl pH 8.0, 50% CH_3_CN in 100 mM Tris-HCl pH 8.0, and CH_3_CN, same as above. The gels were then treated with 2.0 μg Trypsin Gold (Promega) in 100 mM Tris buffer pH 8.0 (700 μL) and incubated at 37 °C overnight. The obtained solution was quenched using TFA aq. (final concn. 0.4%). The sample was desalted using C18 pipette tips (Nikkyo Technos Co., Ltd.). After desalting, the solvent was removed using a centrifugal evaporator. The resulting peptide was dissolved in 5% acetonitrile solution in 0.1%TFA aq. (20 μL).

### NanoLC-MS/MS for identification of labeled proteins in HEK293FT cell nuclear extract

NanoLC-MS/MS analysis was performed using an LC-nano-ESI-MS comprising a quadrupole, orbitrap, and ion trap Tribrid mass spectrometer (Orbitrap Fusion; Thermo Fisher Scientific) equipped with a nanospray ion source and a nano HPLC system (Easy-nLC 1000; Thermo Fisher Scientific). The trap column used for the nano HPLC was a 2 cm × 75 μm capillary column packed with 3 μm C18-silica particles (Thermo Fisher Scientific), and the separation column was a 12.5 cm × 75 μm capillary column packed with 3 μm C18-silica particles (Nikkyo Technos Co., Ltd.). The micropump (flow rate: 300 nL/min) gradient method was used as follows: mobile phase A: 0.1% formic acid; mobile phase B: 80% acetonitrile, 0.1% formic acid aq. 0–1 min: 0–5% B, 1−61 min: 5–40% B, 61−63 min: 40–95% B, 63–80 min: 95% B. The LC-MS/MS data were acquired in a data-dependent acquisition mode controlled by Xcalibur 4.3 (Thermo Fisher Scientific). The settings for the data-dependent acquisition were as follows: maximum injection time, 0.05 s; full MS (MS1, orbitrap) scan range, 375–1500 *m/z*, excluding the former target ion for 20 s; and mass tolerance, 10 ppm. The top speed cycle time was set to 3 s, and the maximum MS2 scan could be acquired within that time. The MS2 (isolation: quadrupole, detection: ion trap) scan maximum injection time and range were 0.035 s and the *m/z* range was defined based on the MS1 scan, respectively. The measurement was performed three times for each sample. The protein identification and label-free quantification were conducted using the Proteome Discoverer 2.5 software embedded with the Sequest algorithm (Thermo Fisher Scientific). The list of human proteins was obtained from the UniProt database (taxonomy 9606; ver. Jun. 14, 2022). Volcano plot analysis was performed using Perseus software ver. 1.6.15.0. The information about nucleotide- or RNA-binding properties for the proteins whose increase rates were more than the third quartile score (178 proteins) was manually curated with the UniProt database.

### Protein labeling for SDS-PAGE (HK1, hnRNP A1, and CPA4)

G4-cat in 50 mM Tris-HCl buffer (pH 7.5) containing 100 mM KCl was annealed by incubation at 90 °C, followed by cooling to 4 °C at a rate of 0.5 °C/min. The labeling reaction was performed in a final volume of 30 μL in the Tris-HCl buffer. Purified proteins (final concentration 1.0 μM), calf thymus DNA (1 ng/μL), and MAUra-N_3_ (from 100 mM stock solution in dimethyl sulfoxide; final concentration, 1 mM) were mixed with photocatalyst-modified HT DNA (final concentration 2.0 μM) and then incubated for 30 min on ice. The reaction mixture was photo-irradiated on ice with a 455-nm LED light, DC8-12V 15 M Max, and 200 mA MAX. For Cys alkylation, 2-iodoacetamide (IAM, final concentration 10 mM) was added to the reaction mixture and incubated for 1 h at room temperature in a dark place. To visualize the labeled proteins, the reaction mixture was treated with DBCO-Cy3 (Sigma-Aldrich, from 1.0 mM stock solution in DMF; final concentration, 100 μM) and incubated for 1 h at 37 °C. After reactions, the labeled proteins were separated by sodium dodecyl sulfate polyacrylamide gel electrophoresis (SDS-PAGE) with 12% (hnRNP A1 and CPA4) or 8% (HK1) gels, respectively. A fluorescence image was obtained using a Molecular Imager PharosFX Plus (Bio-Rad Laboratories, Inc.). The gel was visualized with CBB stain.

### Electrophoretic mobility shift assay (EMSA) for HK1, hnRNP A1, and CPA4 proteins

The binding reaction was conducted in a 2 µL mix [50 mM Tris-HCl (pH 7.5) containing 100 mM KCl, 2 mM DTT, 15% (v/v) glycerol, 1.0 ng/µL calf thymus DNA, 1.0 µM of selected protein, and 5.0 nM of FAM-labeled annealed DNA] for 30 min at 0 °C. The complex was analyzed by electrophoresizing (2 h) on 6% nondenaturing PAGE (19:1 acrylamide: bis-acrylamide (wt/wt)) with 0.5× TBE at 4 °C. The gel was visualized using a Molecular Imager PharosFX Plus (Bio-Rad Laboratories, Inc.). The apparent dissociation constant (*K*_dapp_) was determined by plotting data from three replicates as *φ* (1 − fraction of free DNA) versus protein concentration (P). The binding reactions were performed in a final volume of 20 µL with 2 nM FAM-labeled oligonucleotides and various concentrations of purified protein in 50 mM Tris-HCl (pH 7.5) containing 100 mM KCl, 2 mM DTT, 15% (v/v) glycerol, and 1.0 ng/µL calf thymus DNA. *K*_dapp_ was calculated by nonlinear regression using Microsoft Excel for Microsoft 365 MSO, according to the following equation:

*φ* = [*P*]/ {*K*_dapp_ + [*P*]}

### Oligonucleotide sequences

All target DNA and RNA oligonucleotides used in this study were purchased from JBioS (Japan) or synthesized using DNA with a terminal amine-containing linker. The base sequences are listed below (chemical modifications such as FAM, Texas Red, amine-containing linkers, or photocatalysts are described in the respective method sections):

- *wt* Tel23 (HT G4): 5′-TAGGGTTAGGGTTAGGGTTAGGG-3′
- ssDNA: 5′-CTGAGTGAGTGAGTGAGA-3′
- Complementary strand for dsDNA (*w*/ G): 5′-TCTCACTCACTCACTCAG-3′
- Complementary strand for dsDNA (without G): 5′-TCTCACTCACTCACTCA-3′
- Myc G4 DNA: 5′-TGAGGGTGGGTAGGGTGGGTAA-3′
- NRAS G4 DNA: 5′-GGGAGGGGCGGGTCTGGG-3′
- TERRA G4 RNA: 5′-UAGGGUUAGGGUUAGGGUUAGGG-3′
- KRAS G4 RNA: 5′-GGCGGCGGCAGUGGCGGCGG-3′
- NRAS G4 RNA: 5′-GGGAGGGGCGGGUCUGGG-3′

### Reporting summary

Further information on research design is available in the Nature Portfolio Reporting Summary linked to this article.

## Supplementary information


Transparent Peer Review file
Supplementary Information
Description of Additional Supplementary Files
Supplementary Data 1
Supplementary Data 2
Supplementary Data 3
Reporting Summary


## Data Availability

Raw data from the nanoLC-MS/MS analysis and the search results were deposited in jPOSTrepo (JPST003776) and are available in the ProteomeXchange consortium with the accession number PXD063441. The uncropped gel images and other raw source data underlying this article are available on Figshare at https://doi.org/10.6084/m9.figshare.33032747. The source data for proteomics profiling and statistical analysis (Figs. 5B, C, D, and E) are provided in Supplementary Data 1 (for G4-Ru) and Supplementary Data 2 (for G4-BDP). The source data for the Gene Ontology (GO) enrichment analysis are provided in Supplementary Data 3. The other data underlying this article are available in the article and its online supplementary material.
