# Supplementary material for: Photocatalytic proximity labeling for the identification of G-quadruplex DNA-interacting proteins
Source: Commun Chem. 2026 Aug 27;9:286. doi: 10.1038/s42004-026-02170-9 (PMC13522520; doi:10.1038/s42004-026-02170-9)
Supplement: Supplementary file 1 [file 42004_2026_2170_MOESM1_ESM.pdf]

nature portfolio

Peer Review File

Photocatalytic proximity labeling for the identification of G-quadruplex DNA-interacting proteins

Corresponding Author: Dr Kazumitsu Onizuka

This file contains all reviewer reports in order by version, followed by all author rebuttals in order by version.

Version 0:

Reviewer comments:

Reviewer #1

(Remarks to the Author)
The manuscript by Abdelhady et al. describes a photocatalytic approach for the labelling of G-Quadruplex (G4)-binding proteins. In contrast to the previously adopted approaches, mainly focused either on the direct modification of a nucleic acid with a crosslinking moiety (e.g. the approach described by Chen and colleagues in Nat Chem. 2023 Jun;15(6):803-814. doi: 10.1038/s41557-023-01196-z) or the use of a ligand equipped by photocrosslinking moiety (e.g. the approach developed by Balasubramanian group in Nat. Chem. 13, 626-633 (2021)), this method relies on the modification of a G4 oligonucleotide with a ruthenium photocatalyst that, upon blue-light irradiation, locally enables the reaction of the nearby G4-binding proteins with an external labelling reagent.
Although the concept (as G4-binding protein labelling) is not entirely new, I believe that the manuscript is of good quality and the finding are worth for publication in this journal.
However, I have some concerns that the authors should address before the final publication of the work.

1) One major concern is the use of HT as a model system. Under physiological conditions (e.g. presence of potassium) Tel23 is highly polymorphic, adopting a variety of conformations based on the salt environment. Additionally, telomeric structures are highly sensitive to modifications and mutations (e.g. Chem. Sci., 2019, 10, 218-226 a single A T substitution lead to the formation of a stable antiparallel structure). The authors performed CD analysis, showing the formation of a hybrid G4, and comparing the native G4 with the modified ones. However, as commented by the authors as well in the text, some of the CD spectra reveal partial variation from the original hybrid structure.
Based on this, what was the rationale chosen by the authors to justify the use of HT sequence, rather than more conformationally stable, propeller type sequences, known to bind proteins? This is even more important considering that most of the G4-binding proteins reported so far are known to bind to parallel G4s. This is biologically more relevant, considering that it has been shown that G4 in long sequences/flanks (which is always the case for genomic DNA), they tend to fold more likely into parallel G4s (Nucleic Acids Research, Volume 49, Issue 16, 20 September 2021, Pages 9548–9559, https://doi.org/10.1093/nar/gkab681).

2) Related to the previous point, a brief discussion that their method should in principle be compatible with more conformationally homogeneous parallel sequences (e.g. promoter G4s such as Myc or Kit) and that choice of HT is mainly for historical comparability, if that was the rationale, should be added.

3) Also concerning the conformation point, CD is noteworthy a low resolution technique and could be misleading, as it offers mostly only a qualitative picture. Some other techniques can be used in support of the authors' claim that the conformation does not change significantly, such as native PAGE and other spectroscopic techniques (e.g. UV - Nucleic Acids Research, Volume 53, Issue 18, 14 October 2025, gkaf953, https://doi.org/10.1093/nar/gkaf953,). Given the known polymorphism of HT G4 and the reported CD changes upon modification, the authors should more rigorously document the G4 topology and possible ensemble shifts induced by Ru/BDP and linkers, or at least discuss how this might impact the interpretation of protein-binding and labelling data, which is currently quite limited in the discussion section.

4) In the main text the authors state that singlet oxygen generated by Ru/BDP oxidizes nearby residues, "e.g. histidine," to give endoperoxides trapped by MAUra. Later they mention that Ru can also mediate tyrosine labeling via SET, but they conclude that the singlet oxygen pathway is dominant based on the obtained data. Did the authors consider performing a control experiment with a known singlet oxygen quencher (e.g. sodium azide) to validate this claim?

5) Some more recent studies than ref. 31 cited by the authors suggest that the diffusion distance of singlet oxygen can extend well beyond 10 nm, in some cases up to ~220 nm, which is considerably larger than the value used to define the labeling radius in this work (e.g. Photochem. Photobiol. 2006, 82, 1178–1186). This relatively long effective diffusion distance could, in principle, permit labeling of proteins that are not in direct contact with the G4, but rather reside within the broader microenvironment. While a control experiment with the free photocatalyst is already partially addressing this concern (where "off-label" reaction are indeed seen), a more explicit discussion of how 'local' the authors consider their labeling to be, in light of the range of reported singlet oxygen diffusion lengths, would strengthen the mechanistic interpretation of the method.

6) Another major concern is the self-damage of the probes upon light irradiation. The authors mainly performed PAGE analysis to check on the DNA damage and self-labelling. It is known that the formation of 8-oxoG lead to a conformational change and unfolding of the G4. This could potentially lead to progressive unbinding of the G4-binding proteins over the course of the irradiation. I believe that a CD analysis/ EMSA prior and after the irradiation to check the G4 integrity and the binding to protein, should be performed.

Overall, I am supportive for the publication of this study in Communications Chemistry, upon revision of the present version of the manuscript. I am happy to re-evaluate the revised version with extra experiments and/or further clarification and discussion from the authors.

Reviewer #2

(Remarks to the Author)
In this article, the authors report a strategy for the identification of G-quadruplex-interacting proteins. To do so, the authors equipped a G-quadruplex (G4) sequence of the human telomere with a ruthenium complex or a BODIPY photocatalyst either at a terminal or an internal, loop position. The authors then demonstrated that the introduction of these modifications did not impair G4 structure formation. The modified sequences were then used in a photolabeling experiment with unwinding protein 1 which allowed to identify the best site for modification of G4, efficiency of the reaction, and which amino acid side-chains were involved. The authors also demonstrated that none of the modified G4 structure induced significant self-labelling under irradiation conditions. The authors then applied this method to a nuclear extract of proteins stemming from HEK293FT cells. A thorough analysis allowed to compare the nature of proteins identified with both modified G4 structures as well as control reactions with free photocatalysts, ss-cat, and ds-cat. In addition, this analysis allowed the identification of one protein, hexokinase-1 (HK1), which was not known to interact with G4 structures. The authors then photolabelled HK1 with modified G4 and determined the Kd value of an HK1-G4 complex by EMSA. The manuscript is very well-written and clear, all experiments were carried out in a competent manner, and the supporting information is of good quality. The method developed in this manuscript will be of high interest to a large community interested in identifying G4-protein interactions. Hence, publication in Communications Chemistry is recommended pending some minor revisions:

- Page 8 and Figure S10: the authors mention that "the SET mechanism is possible, and tyrosine can be labeled". However, no data to support this claim was provided. The authors should either show that tyrosine can indeed be labelled or remove this statement.
- Figure S11: gel image shown in part C) reveals a non-negligible proportion of a faster running band. Couldn't this be caused by damage to the G4 structure?
- Have the authors detected any damage to cysteines or disulfide bonds (10.1016/j.freeradbiomed.2023.08.024) after light irradiation?
- The authors may want to describe photocrosslinking agents-containing oligonucleotides for photolabeling to proteins in the introduction (see e.g. 10.1002/anie.202213764; 10.1002/chem.202104208; 10.1002/cbic.200800397; 10.1039/C0CC02450C) and may want to cite a recent article on Ru-containing aptamer (10.1021/jacs.5c13224).

Reviewer #3

(Remarks to the Author)
The study of Abdelhady et al. reports a photocatalytic proximity labeling (PPL) strategy based on Ru- and BODIPY-functionalized G4 DNA to identify G4-interacting proteins. The experimental design is generally sound and supported by appropriate controls; however, several critical issues regarding the conceptual advance, data interpretation, and protein validation substantially limit the impact and general applicability of the work. On this basis, this reviewer recommends major revision before the manuscript can be considered for publication.
Main concerns:
1. The rationale for selecting Ru complexes and BODIPY as photocatalysts is not sufficiently justified. In particular, the BODIPY-base system appears to suffer from quenching effects and reduced labeling efficiency depending on the local environment, which limits its practical utility. It is therefore unclear why this probe remains a central element of the study. A clearer justification, along with a more critical discussion of its limitations and possible alternatives, is needed.
2. The manuscript does not convincingly demonstrate a clear conceptual advance over existing methods (such as G4-LIMCAP, CMPP, and related proximity labeling strategies). The proposed advantage of avoiding ligand competition is not experimentally supported, as the substantial overlap with previously identified G4-interacting proteins raises concerns about the added value of the proposed methodology. The authors should more explicitly define what distinguishes their approach from others in terms of results.

3. A key limitation is the inability to distinguish direct binders from indirect interactors. The large number of identified candidates suggests limited specificity. The absence of orthogonal validation methods to demonstrate direct binding and of filtering strategies weakens the biological interpretation. Additional analyses, such as STRING network analysis, could help identify interaction patterns and improve data interpretation.
4. The study focuses almost exclusively on a single candidate protein (HK1) for validation. While this provides an initial proof of concept, it is not sufficient to validate a global proteomics approach. Validation of additional independent candidates would significantly strengthen the conclusions.
5. While EMSA is acceptable for qualitative binding assessment, its use for quantitative determination of Kd values is not ideal and may lack accuracy. More robust biophysical techniques (e.g., fluorescence, ITC, MST, or SPR) would strengthen the conclusions.
Minor concerns:
6. A Gene Ontology analysis should be useful to support the section on “Global profiling of G4 DNA-interacting proteins using PPL” and to provide functional context.
7. Possible inconsistencies between Figures S3 and S4 should be checked and corrected.

Version 1:

Reviewer comments:

Reviewer #1

(Remarks to the Author)
After reading this revised version, I am now convinced that the manuscript could be accepted for publication in Communications Chemistry. The authors did a meticulous work in solving all the concerns raised, performed new experiments, extended the discussion and strengthened, in my opinion, the conclusion of the manuscript.
Very minor point, the authors may want to cite this recent paper about in situ proximity labelling of rG4-binding proteins by Weng group (Mo, J., Chen, Z., Cui, M. et al. In situ proximity labeling of proteins associated with circRNA and RNA G-quadruplexes. Nat Chem Biol 22, 874–885 (2026). https://doi.org/10.1038/s41589-025-01993-2), and these two papers by Madder group about proximity (photo)labelling of G4 and G4-binding peptides (Barr, J., Colpaert, G., Waeyenberge, L., Cadoni, E., & Madder, A. (2026). Singlet oxygen-induced covalent trapping of G4-binding peptides onto their cognate target G4. Cell Reports Physical Science, 7(5), 103267. https://doi.org/10.1016/j.xcrp.2026.103267 and de Paepe, L., Marzano, S., Vesschemoet, C., Amato, J., Pagano, B., Cadoni, E., & Madder, A. (2026). From alkylating to shape-shifting G-quadruplex ligands: the RHAU peptide story. Nucleic Acids Research, 54(3), 54. https://doi.org/10.1093/NAR/GKAG039), if considered appropriate for literature coverage.

Reviewer #2

(Remarks to the Author)
The authors have revised this manuscript to account for the comments of three reviewers (I was Referee#2). My original review included various comments, and the authors have now comprehensively addressed all the points that I raised and certainly markedly improved the quality of the manuscript. The authors have carried out additional experiments as were suggested by most reviewers. Particularly, the authors carried out native gel electrophoresis to evaluate potential changes in configuration (reviewer#1), a control experiment to demonstrate that singlet oxygen was the active species (reviewer#1), additional CD spectroscopy experiments to assess potential damage to dG units after photoirradiation (reviewers#1 and #2), analysis of photodamage by MS on disulfide linkages (reviewer#2), and STRING network analysis and MST (reviewer#3). The response to the other reviewers was also satisfactory. Hence, publication in Communications Chemistry is now recommended without any additional revisions.

Reviewer #3

(Remarks to the Author)
The authors have made revisions and have performed additional experiments that improved the manuscript. In particular, the added controls addressing photocatalyst-induced perturbation, singlet oxygen dependence, and structural integrity of the probes strengthen the methodological section. However, the main concerns raised in the previous review have not been fully resolved, and several points still require clarification or additional experimental support before publication.

The revised manuscript still relies almost exclusively on HK1 as the validation target. While the additional discussion of this limitation is appreciated, validation of a single new candidate is not sufficient to support broad conclusions regarding the fidelity, specificity, and general reliability of the global interactome generated by this approach. Validation of additional independent candidates would substantially strengthen confidence in the screening strategy. This should include both enriched proteins and appropriate non-enriched or low-ranked controls. If additional validation is not feasible, the authors should further temper the general claims regarding the specificity and global reliability of the method.

Related to this point, parts of the revised discussion still appear to overinterpret individual candidates or network-analysis results. The STRING analysis provides useful functional context, but it does not establish direct G4 binding or demonstrate that specific candidates represent distinct biological pathways or mechanistic classes. In particular, the observed separation

of HK1 in the STRING network does not by itself demonstrate a distinct mechanistic role for HK1 in G4 biology. This interpretation should therefore be presented more cautiously.

The addition of MST is appreciated. However, quantitative affinity measurements were performed only for HT G4, whereas the manuscript extends the interpretation toward broader G4 recognition by HK1. Because EMSA experiments were expanded to multiple G4 structures, it would be more convincing to provide quantitative binding measurements for at least selected additional G4 targets rather than relying exclusively on qualitative EMSA assessment. If this is not possible, the claims regarding broader G4 recognition should be clearly described as qualitative.

Furthermore, the substantial discrepancy between the original EMSA-derived apparent Kd and the newly reported MST Kd deserves more careful discussion, particularly considering that high-concentration data points were excluded from MST fitting. Additionally, I noticed an internal inconsistency that should be clarified before the affinity data can be properly evaluated. In the rebuttal and revised Results section, the MST experiment is described as being performed using FAM-labeled HT G4 DNA. However, the Supporting Information (Methods and Figure S21 legend) describes the MST experiment as being performed using Texas Red-labeled HT G4 at 20 nM. Moreover, the inconsistency appears to involve not only the fluorophore and DNA concentration, but also the buffer composition and KCl concentration, which are particularly relevant for G4 folding and MST-derived affinity estimates. Please clarify which fluorescently labeled construct was actually used (FAM or Texas Red), which DNA concentration and buffer conditions were applied, and whether the reported Kd = 11.8 nM originates from the same experiment described in the Supplementary Information. Because fluorophore identity, DNA concentration, and buffer composition can influence fluorescence behavior, G4 folding, thermophoretic response, and apparent affinity estimates in MST experiments, especially in the presence of reported fluorescence quenching at high protein concentrations, this inconsistency should be corrected and reconciled throughout the manuscript and Supporting Information. Finally, because the reported affinity is substantially stronger than the EMSA-derived estimate, the robustness of the fit becomes especially important. To allow independent assessment of data quality, I suggest providing also the raw MST traces and capillary fluorescence data.

Overall, the revised manuscript is improved, but the current evidence still does not fully support the broader claims made for the global interactome. Additional candidate validation, a clearer presentation of the MST data, and a more cautious interpretation of STRING/HK1-related conclusions are needed before the manuscript can be considered suitable for publication.

Open Access This Peer Review File is licensed under a Creative Commons Attribution 4.0 International License, which permits use, sharing, adaptation, distribution and reproduction in any medium or format, as long as you give appropriate credit to the original author(s) and the source, provide a link to the Creative Commons license, and indicate if changes were made.
In cases where reviewers are anonymous, credit should be given to 'Anonymous Referee' and the source.
The images or other third party material in this Peer Review File are included in the article’s Creative Commons license, unless indicated otherwise in a credit line to the material. If material is not included in the article’s Creative Commons license and your intended use is not permitted by statutory regulation or exceeds the permitted use, you will need to obtain permission directly from the copyright holder.
To view a copy of this license, visit https://creativecommons.org/licenses/by/4.0/

Point-by-point responses to the reviewers' comments

Reviewer #1: We sincerely thank Reviewer 1 for the insightful comments and constructive suggestions, which have greatly helped us improve the clarity, rigor, and depth of the revised manuscript. We have made the necessary additions and corrections accordingly.

1-1: One major concern is the use of HT as a model system. Under physiological conditions (e.g. presence of potassium) Tel23 is highly polymorphic, adopting a variety of conformations based on the salt environment. Additionally, telomeric structures are highly sensitive to modifications and mutations (e.g. Chem. Sci., 2019, 10, 218-226 a single AT substitution lead to the formation of a stable antiparallel structure). The authors performed CD analysis, showing the formation of a hybrid G4, and comparing the native G4 with the modified ones. However, as commented by the authors as well in the text, some of the CD spectra reveal partial variation from the original hybrid structure.
Based on this, what was the rationale chosen by the authors to justify the use of HT sequence, rather than more conformationally stable, propeller type sequences, known to bind proteins? This is even more important considering that most of the G4-binding proteins reported so far are known to bind to parallel G4s. This is biologically more relevant, considering that it has been shown that G4 in long sequences/flanks (which is always the case for genomic DNA), they tend to fold more likely into parallel G4s (Nucleic Acids Research, Volume 49, Issue 16, 20 September 2021, Pages 9548–9559, https://doi.org/10.1093/nar/gkab681).
1-2: Related to the previous point, a brief discussion that their method should in principle be compatible with more conformationally homogeneous parallel sequences (e.g. promoter G4s such as Myc or Kit) and that choice of HT is mainly for historical comparability, if that was the rationale, should be added.

[Answer 1-1 & 1-2] We thank the reviewer for these insightful comments. We agree with the reviewer's perspective regarding the biological relevance and structural homogeneity of parallel G4s. As the reviewer correctly pointed out, our primary rationale for selecting the human telomeric (HT) sequence as the initial model system was based on its historical role as the most extensively characterized G4 structure. Because both the structural folding behaviors and the interacting proteome of the HT sequence have been thoroughly investigated over the decades, it serves as the ideal standard benchmark. This rich background knowledge allowed us not only to strictly evaluate the structural integrity of our probes (e.g., via CD spectra) but also to robustly validate the biological fidelity of our newly developed PPL methodology by successfully capturing well-established HT G4-binding proteins (such as hnRNP A1) as positive controls.

Furthermore, we completely agree that applying this method to conformationally homogeneous parallel G4s is biologically crucial. Our PPL method is, in principle, entirely sequence-agnostic and

fully compatible with such parallel sequences (e.g., c-Myc, c-Kit). In fact, as demonstrated in the manuscript (Figure S22), we have already validated the strong interaction of our highlighted candidate, HK1, with the c-Myc and NRAS promoter G4—a well-known parallel G4 structure. Encouraged by your constructive comment, we are currently designing our next comprehensive proteomics project utilizing parallel promoter G4 sequences to further explore the functional G4-interactome.

To clarify the rationale for using HT and explicitly state the compatibility with parallel sequences, we have expanded the Results and Discussion section in the revised manuscript as suggested.

Results and Discussion: (Page 4)

"For the molecular design of our photocatalyst-conjugated DNA probe, the HT sequence was deliberately chosen as the initial model system. The main reason for this choice is that the HT sequence is one of the most extensively characterized G4 structures to date. Because a substantial body of historical data is available on both its structural folding behaviors[35, 36] and its interacting proteome,[7, 8, 37, 38] it serves as an ideal, data-rich benchmark. Using this well-established system allows us not only to assess the structural integrity of our modified probes but also to validate the biological fidelity of our newly developed PPL methodology by accurately identifying known G4-binding partners as internal positive controls."

1-3: Also concerning the conformation point, CD is noteworthy a low resolution technique and could be misleading, as it offers mostly only a qualitative picture. Some other techniques can be used in support of the authors' claim that the conformation does not change significantly, such as native PAGE and other spectroscopic techniques (e.g. UV - Nucleic Acids Research, Volume 53, Issue 18, 14 October 2025, gkaf953, https://doi.org/10.1093/nar/gkaf953,). Given the known polymorphism of HT G4 and the reported CD changes upon modification, the authors should more rigorously document the G4 topology and possible ensemble shifts induced by Ru/BDP and linkers, or at least discuss how this might impact the interpretation of protein-binding and labelling data, which is currently quite limited in the discussion section.

[Answer 1-3] We appreciate the reviewer's suggestion to employ orthogonal techniques, such as the recently reported UV-based method (Nucleic Acids Res. 2025) and native PAGE, to further validate the G4 topology.

Regarding the UV-based approach, while we agree it is a highly informative tool, we encountered a fundamental biophysical limitation. We measured the UV absorbance of the free Ru and BDP catalysts and found that both molecules exhibit absorption in the UV region below 300 nm (Figure R1). This background absorbance overlaps with the intrinsic UV signatures of the nucleic acids, rendering UV-dependent topological analyses inapplicable for our specific photocatalyst-modified probes.

Alternatively, as suggested, we performed native polyacrylamide gel electrophoresis (native PAGE) for all photocatalyst-modified G4 sequences alongside the unmodified native HT G4 as a reference

(Figure S2). Native PAGE analysis revealed that G4-BDP-term, G4-Ru-term, and G4-Ru-loop migrated as relatively sharp single bands with slightly lower mobility compared to the unmodified native HT G4, which is reasonably attributable to the steric bulk of the covalently attached photocatalysts. In contrast, G4-BDP-loop exhibited a slightly broader band compared to the other constructs. This subtle broadening, combined with the mixed CD profile (showing both 295 nm and 265 nm peaks), suggests that BDP modification at the loop position slightly perturbs the original hybrid topology, leading to a dynamic and heterogeneous ensemble of G4 conformations under our assay conditions.

Crucially, we would like to clarify that G4-BDP-loop was strategically excluded from our primary global proteomics and downstream validation assays due to a combination of this conformational heterogeneity and significant fluorescence quenching observed upon loop modification. Therefore, the core biological conclusions of this manuscript—including the identification of the G4-interacting proteomics network—rely exclusively on the other three constructs (G4-BDP-term, G4-Ru-term, and G4-Ru-loop), all of which maintain their hybrid-type G4 topologies.

By clarifying the limitation of G4-BDP-loop and highlighting that it does not impact our main data, we have expanded the Results and Discussion section and added the native PAGE data to the Supporting Information.

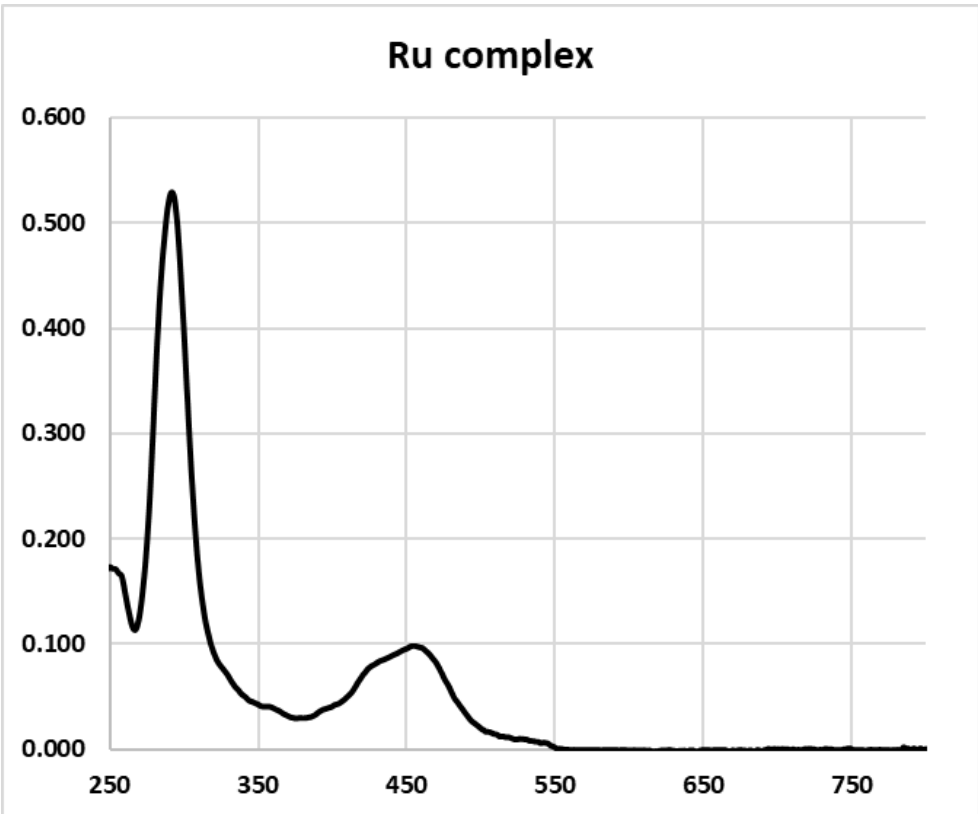

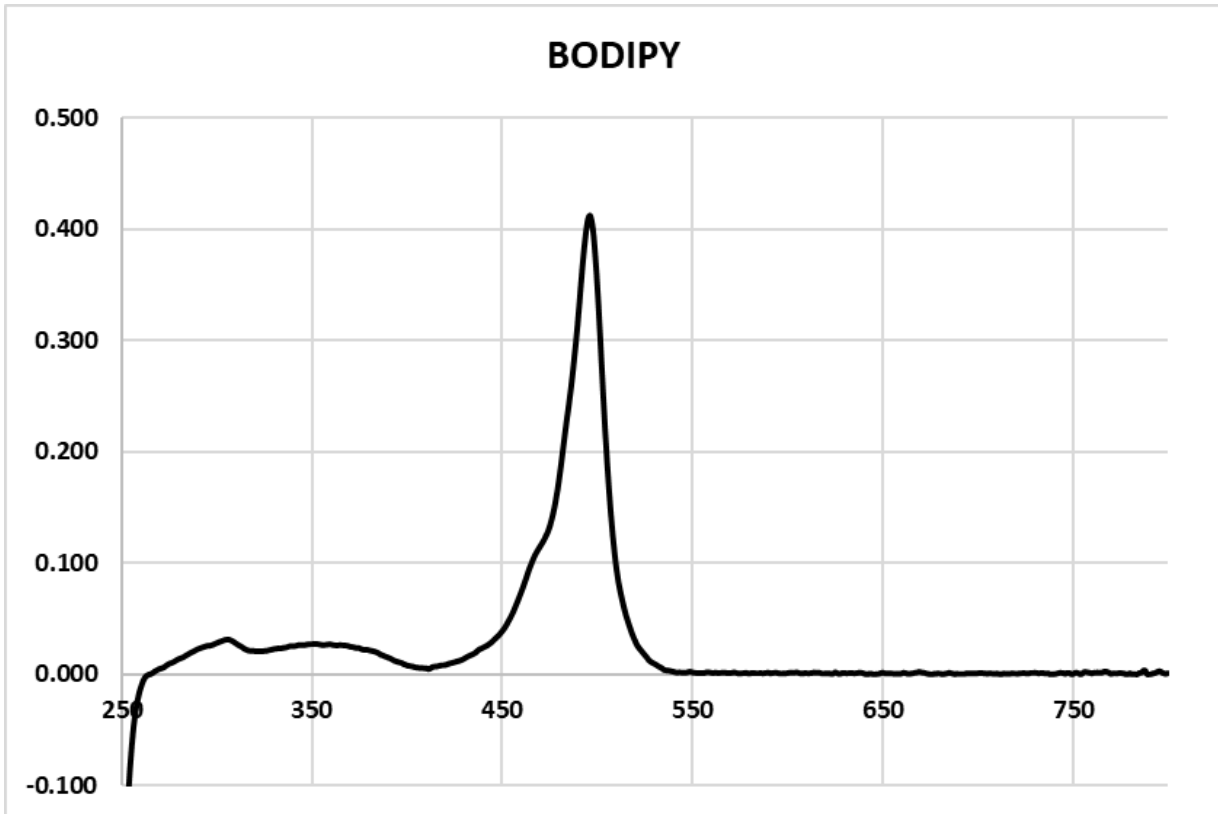

Figure R1. UV-Vis absorption spectra of the free Ru complex and BODIPY photocatalysts.

Results and Discussion: (Page 5)

"To further examine possible conformational effects caused by chemical modification, native PAGE analysis was performed (Supplementary Figure S2). The constructs G4-BDP-term, G4-Ru-term, and G4-Ru-loop migrated as sharp single bands, suggesting that their overall hybrid-type topology is preserved with minimal disturbance beyond the expected steric bulk of the catalysts. In contrast, the G4-BDP-loop showed a slightly broader band migration pattern. Considered together with its mixed CD profile, this broadening suggests that modification in the loop region slightly perturbs the original hybrid topology, resulting in a dynamic ensemble of G4 conformations under our assay conditions."

(Page 10)
"Owing to its severe quenching and conformational heterogeneity, G4-BDP-loop was excluded from the mass spectrometry-based global proteomics analysis to ensure the integrity of our overall interactome datasets."

1-4: In the main text the authors state that singlet oxygen generated by Ru/BDP oxidizes nearby residues, "e.g. histidine," to give endoperoxides trapped by MAUra. Later they mention that Ru can also mediate tyrosine labeling via SET, but they conclude that the singlet oxygen pathway is dominant based on the obtained data. Did the authors consider performing a control experiment with a known singlet oxygen quencher (e.g. sodium azide) to validate this claim?

[Answer 1-4] We thank the reviewer for this constructive suggestion. To experimentally and rigorously validate our claim that singlet oxygen ($^1O_2$) is the dominant reactive species in the MAUra-based proximity labeling, we performed a control photolabeling experiment using sodium azide (NaN_3), a well-established $^1O_2$ quencher.

Specifically, UP1 was incubated with each of the four photocatalyst-modified G4 constructs (G4-BDP-term, G4-BDP-loop, G4-Ru-term, and G4-Ru-loop) and MAUra-N_3 in the presence or absence of NaN_3 (at a final concentration of 10 mM), followed by photo-irradiation at 455 nm under our standard conditions. After the reaction with DBCO-Cy3, the fluorescence intensity of the labeled UP1 band was quantified by SDS-PAGE (New Figure S12). As shown in the new figure, in the presence of NaN_3, the fluorescence labeling of UP1 was substantially reduced (>90% reduction) for all constructs tested compared to the control without the quencher. This robust suppression provides strong experimental support for our conclusion that the photolabeling reaction is predominantly mediated by the singlet oxygen pathway in our system.

These results have been incorporated into the revised manuscript. The text in the Results and Discussion section has been updated to include this finding, and the detailed quenching experiment protocol along with the corresponding figure (Figure S12) have been added to the Supporting Information (Section 1.12).

Results and Discussion: (Page 9)
"To confirm that singlet oxygen ($^1O_2$) is the predominant reactive species in MAUra-based PL, we performed a quenching experiment using sodium azide (NaN_3), a well-established $^1O_2$ quencher. UP1 was incubated with each photocatalyst-modified G4 construct and MAUra-N_3 in the presence or absence of 10 mM NaN_3, followed by standard photoirradiation at 455 nm. Subsequent SDS-PAGE analysis revealed that fluorescence labeling of UP1 was substantially reduced (>90% reduction) in the presence of NaN_3 across all constructs tested (Supplementary Figure S12). This strong suppression provides compelling experimental evidence that the photolabeling reaction in our system is predominantly mediated by the singlet oxygen pathway."

1-5: Some more recent studies than ref. 31 cited by the authors suggest that the diffusion distance of singlet oxygen can extend well beyond 10 nm, in some cases up to ~220 nm, which is considerably larger than the value used to define the labeling radius in this work (e.g. Photochem. Photobiol. 2006, 82, 1178–1186). This relatively long effective diffusion distance could, in principle, permit labeling of proteins that are not in direct contact with the G4, but rather reside within the broader microenvironment. While a control experiment with the free photocatalyst is already partially addressing this concern (where "off-label" reaction are indeed seen), a more explicit discussion of how 'local' the authors consider their labeling to be, in light of the range of reported singlet oxygen diffusion lengths, would strengthen the mechanistic interpretation of the method.

[Answer 1-5] We agree with the reviewer's insightful point and appreciate the reference provided. We acknowledge that the diffusion distance of singlet oxygen ($^1O_2$) can extend up to ~220 nm in pure aqueous solutions (*Photochem. Photobiol.* 2006, 82, 1178–1186). However, in the complex biological matrix of our assay, even though the nuclear extract is diluted with buffer, it contains a vast array of diverse proteins and other biomolecules. The abundant reactive amino acid residues (e.g., histidine, tryptophan, and methionine) within this mixture act as highly efficient endogenous quenchers, significantly shortening the $^1O_2$ lifetime compared to that in pure water. Based on these chemical dynamics in biological extracts, we consider the "local" labeling radius in our system to be restricted to a macromolecular scale (estimated to be within a few tens of nanometers).

As the reviewer pointed out, even within this restricted local environment, our PPL approach captures not only direct G4-binders but also indirect interactors or neighboring members within larger multiprotein complexes. While this means the method cannot independently distinguish direct physical interactors from indirect complex members without orthogonal validation, we view this inherent characteristic as a powerful asset for mapping higher-order networks. We have now explicitly defined our interpretation of "local" and discussed this unique capability in the revised Conclusions section.

Conclusions: (Page 16)

"Furthermore, although singlet oxygen can diffuse up to ~220 nm in pure water[52], abundant endogenous quenchers in the nuclear extract limit its effective labeling radius to a local macromolecular scale (tens of nanometers). Within this microenvironment, our PPL approach captures both direct G4 binders and indirect complex members. Rather than being a limitation, this capability offers a distinct advantage by enabling comprehensive mapping of the functional, higher-order interaction networks that dynamically assemble around G4 scaffolds and are often lost in conventional direct-binding assays."

1-6: Another major concern is the self-damage of the probes upon light irradiation. The authors mainly performed PAGE analysis to check on the DNA damage and self-labelling. It is known that the formation of 8-oxoG lead to a conformational change and unfolding of the G4. This

could potentially lead to progressive unbinding of the G4-binding proteins over the course of the irradiation. I believe that a CD analysis/ EMSA prior and after the irradiation to check the G4 integrity and the binding to protein, should be performed.

[Answer 1-6] We thank the reviewer for pointing out this critical aspect regarding potential photo-induced damage, such as the formation of 8-oxoG, which could potentially lead to G4 unfolding.

To directly address this concern, we performed complementary experiments—CD spectroscopy and electrophoretic mobility shift assay (EMSA)—to assess both the structural integrity and the functional protein-binding ability of the probes before and after standard photo-irradiation.

For CD spectroscopy, G4-Ru-loop and G4-Ru-term were irradiated at 455 nm for 30 seconds, and G4-BDP-loop and G4-BDP-term for 10 minutes. While a slight decrease in the overall CD ellipticity was observed for G4-Ru after irradiation, the spectral shapes and diagnostic peak positions remained essentially unchanged (New Supplementary Figure S14). This indicates that although minor photo-damage may occur, the global G4 topologies are adequately maintained.

Furthermore, we confirmed by EMSA that the functional G4-binding capability is preserved. When the irradiated modified G4 (FAM-labeled, 5.0 nM) was incubated with UP1 (1.0 μM), the band-shift patterns were comparable to those of the non-irradiated controls, confirming that UP1 binding to the G4 structure is maintained even after photo-irradiation (New Supplementary Figure S15).

Importantly, the CD measurements were performed in a simple buffer without proteins, representing a relatively harsh condition where the naked DNA is fully exposed. In our actual proximity labeling assays using nuclear extracts, target proteins physically bind to the G4 structures. This protein binding effectively covers and protects the DNA from reactive species through steric shielding (a footprinting-like effect). Combined with the abundant surrounding proteins acting as competitive singlet oxygen quenchers, the oxidative damage to the DNA is expected to be substantially mitigated in the actual labeling environment.

Together, these findings alleviate the concern that irradiation leads to progressive G4 unfolding or the unbinding of interactors, ensuring that structural disruption does not critically compromise our assay. We have incorporated these results into the revised manuscript, and the new figures have been added to the Supporting Information.

Results and Discussion: (Page 9-10)

"We also evaluated the structural and functional integrity of the probes before and after photoirradiation. CD spectroscopy showed that although a slight decrease in overall ellipticity was observed after standard irradiation, the characteristic G4 spectral profiles and peak positions remained essentially unchanged for all constructs (Supplementary Figure S14). This indicates that the overall G4 topologies are preserved. Furthermore, EMSA confirmed that the binding affinity of UP1 for the irradiated G4 probes was maintained, with band-shift patterns comparable to those of the non-irradiated controls (Supplementary Figure S15). Taken together, these validations confirm that both the G4 topologies and their protein-binding capabilities are adequately preserved during the labeling reaction, ensuring the reliability of our interactome analysis."

Reviewer #2: We sincerely thank Reviewer 2 for the positive evaluation of our work and the encouraging remarks. We greatly appreciate the constructive comments and the helpful literature suggestions, which have further refined the clarity and completeness of the revised manuscript. We have carefully addressed all the points and made the necessary corrections accordingly.

2-1: Page 8 and Figure S10: the authors mention that "the SET mechanism is possible, and tyrosine can be labeled". However, no data to support this claim was provided. The authors should either show that tyrosine can indeed be labelled or remove this statement.

[Answer 2-1] We thank the reviewer for this careful observation. We agree that speculative claims without direct experimental evidence should be avoided. To avoid any speculative claims, we have removed the statement regarding the SET mechanism and tyrosine labeling from the revised text.

2-2: Figure S11: gel image shown in part C) reveals a non-negligible proportion of a faster running band. Couldn't this be caused by damage to the G4 structure?

[Answer 2-2] We thank the reviewer for this accurate observation. We agree that the minor faster-running band of irradiated G4-BDP-term (Figure S11C, new: S13C) indeed indicates slight photodegradation of the DNA probe. We have revised the main text to accurately reflect this observation, acknowledging the minor degradation while noting that the vast majority of the probe remains intact.

Results and Discussion: (Page 9)

"For the G4-BDP-term, although a slightly faster-migrating band suggesting minor photodegradation was observed upon photoirradiation, the vast majority of the G4 probe remained intact (Supplementary Figure S13C right, lanes 2–4). Additionally, negligible G4 labeling was observed in the presence of MAUra-N3 with or without calf thymus DNA (Supplementary Figure S13C right, lanes 3 and 4)."

2-3: Have the authors detected any damage to cysteines or disulfide bonds (10.1016/j.freeradbiomed.2023.08.024) after light irradiation?

[Answer 2-3] We thank the reviewer for raising this important point regarding potential oxidative damage to amino acid residues during photocatalytic labeling. We have carefully considered this issue and provide the following analysis.

First, we note an inherent experimental limitation: standard bottom-up proteomics sample preparation includes a reduction and alkylation step (e.g., TCEP/chloroacetamide), which reduces disulfide bonds and reversible cysteine oxidation products prior to digestion. As a consequence, reversible oxidative modifications—including methionine sulfoxide (+O on Met) and disulfide bond disruption—are not retained in the processed samples and cannot be assessed in our datasets. This is a general limitation shared by most proximity labeling proteomics workflows.

However, to address the reviewer's concern regarding irreversible oxidative damage, we performed a re-analysis of our mass spectrometry data, specifically searching for the following irreversible oxidative modifications: mono-oxidation (+O on His, Trp, Tyr), oxidation with water loss (+O−H_2O on His, Trp, Tyr), and di-oxidation (+O_2 on His, Met, Trp, Tyr). We then examined whether peptides enriched by proximity labeling (i.e., those showing differential abundance between catalyst-targeted and control conditions) were correlated with increased oxidative modification levels. As a representative comparison, we analyzed the G4-photocatalyst-targeted condition versus the free photocatalyst control. The results showed no significant correlation between labeling-dependent peptide enrichment and the abundance of oxidatively modified peptides. This finding suggests that proteins in close proximity to the photocatalyst do not undergo notably greater irreversible oxidative damage than those in the bulk population, indicating that the labeling conditions employed in our system do not induce appreciable oxidative perturbation at the proteome level.

2-4: The authors may want to describe photocrosslinking agents-containing oligonucleotides for photolabeling to proteins in the introduction (see e.g. 10.1002/anie.202213764; 10.1002/chem.202104208; 10.1002/cbic.200800397; 10.1039/C0CC02450C) and may want to cite a recent article on Ru-containing aptamer (10.1021/jacs.5c13224).

[Answer 2-4] We thank the reviewer for these excellent literature suggestions. We agree that citing these precedent studies provides a more comprehensive and accurate background for our work. We have carefully incorporated these relevant references into the Introduction section to properly introduce oligonucleotide-based photolabeling and Ru-containing aptamer strategies.

Introduction: (Page 2)

"Chemical labeling methods using photosensitive functional groups (e.g., diazirine, aryl azide, and benzophenone) or electrophiles (chloroacetamide, glyoxal, and tosyl groups) introduced onto nucleic acids circumvent the use of engineered fusion proteins and enable target protein tagging.[13-18]"

(Page 3)

"Indeed, Ru-containing functional oligonucleotides, such as aptamers, have recently been developed as targeted photosensitizing agents for photodynamic therapy.[31]"

Reviewer #3: We sincerely thank Reviewer 3 for the rigorous evaluation and highly constructive feedback. The reviewer’s insightful comments, particularly the suggestions to perform network and functional pathway analyses, have been immensely valuable in strengthening the biological interpretation and the overall impact of our study. We have thoroughly addressed all the concerns and made the necessary revisions as detailed below.

3-1: The rationale for selecting Ru complexes and BODIPY as photocatalysts is not sufficiently justified. In particular, the BODIPY-base system appears to suffer from quenching effects and reduced labeling efficiency depending on the local environment, which limits its practical

utility. It is therefore unclear why this probe remains a central element of the study. A clearer justification, along with a more critical discussion of its limitations and possible alternatives, is needed.

[Answer 3-1] We thank the reviewer for this constructive critique regarding our choice of photocatalysts and the limitations of the BODIPY-based system. We apologize for the lack of clarity in our previous draft and appreciate the opportunity to clarify the central rationale for including the BODIPY probe.

Our initial rationale for selecting both Ru and BODIPY was to systematically evaluate and compare the performance of two physicochemically distinct classes of photocatalysts—a bulky, cationic transition-metal complex (Ru) versus a relatively compact, neutral organic dye (BODIPY)—in the context of DNA-conjugated proximity labeling. As the reviewer correctly pointed out, we found that the BODIPY-loop construct suffered from severe intramolecular quenching by adjacent guanine bases, which significantly compromised its utility, and it was therefore excluded from the global proteomics assays.

However, the terminal-modified BODIPY probe (G4-BDP-term) maintained sufficient labeling efficiency despite experiencing a slight degree of guanine quenching. Crucially, we included G4-BDP-term in our global proteomics profiling to serve as an indispensable control for assessing potential catalyst-dependent biases. Strikingly, the proteomics data obtained with G4-BDP-term exhibited a substantial correlation with that of G4-Ru-term. This strong consistency demonstrates that the drastic differences in the physicochemical properties of the photocatalysts (e.g., charge, steric bulk, and structural class) do not induce artificial bias in the identified protein profiles, validating the generality and fidelity of our PPL methodology.

Therefore, the BODIPY probe remains a key element because it provides essential methodological insights into structural limitations (as seen with the loop construct) while simultaneously confirming the absence of catalyst-dependent bias (as seen with the terminal construct). We have extensively revised the Discussion section to critically address the limitations of BODIPY and to highlight the valuable insights gained from this comparative study.

Introduction: (Page 4)

"In this study, we systematically compared these two catalysts to evaluate how physicochemically distinct molecules—a bulky, cationic transition-metal complex versus a relatively compact, neutral organic fluorophore—behave when conjugated to specific local microenvironments of G4 DNA structures."

Conclusions: (Page 16)

"By systematically comparing distinct photocatalysts (a Ru complex and BDP) in this profiling, we showed that their interactome profiles are strongly correlated, confirming that our PPL approach is free of catalyst-dependent bias. However, because BDP is susceptible to intramolecular quenching by nearby guanine bases, the Ru complex is the optimal choice for this profiling."

3-2: The manuscript does not convincingly demonstrate a clear conceptual advance over existing methods (such as G4-LIMCAP, CMPP, and related proximity labeling strategies). The proposed advantage of avoiding ligand competition is not experimentally supported, as the substantial overlap with previously identified G4-interacting proteins raises concerns about the added value of the proposed methodology. The authors should more explicitly define what distinguishes their approach from others in terms of results.

[Answer 3-2] We thank the reviewer for this critical comment, which allows us to clarify the unique conceptual advance of our method and its complementary relationship with existing ligand-based approaches. Regarding the concern about the substantial overlap with previously identified G4-interacting proteins, we view this consensus as a highly positive and biologically meaningful outcome. Because our PPL method operates on fundamentally different physicochemical principles compared to non-covalent, diazirine-based methods (such as G4-LIMCAP and CMPP), this overlap robustly cross-validates the reliability of both strategies in capturing authentic G4 interactors.

Beyond this shared baseline, the conceptual advance of our PPL method is two-fold, providing a distinct analytical perspective. First, unlike conventional stoichiometric (1:1) photo-crosslinking, our photocatalytic approach enables continuous signal amplification, offering the inherent advantage of enhanced sensitivity for capturing proximal proteins. Second, and more importantly in terms of empirical results, our method features unique positional freedom (site-flexibility). Ligand-based methods are inherently tethered to specific fixed binding pockets, which can inadvertently cause local steric hindrance or displace certain native binders depending on the microenvironment.

In contrast, our covalently targeted PPL approach allows for the strategic repositioning of the photocatalyst (e.g., terminal vs. loop). The practical value of this site-flexibility is exemplified by the profiling of Hexokinase-1 (HK1). HK1 was effectively captured by the terminal-modified probe (G4-Ru-term) but not by the loop-modified counterpart. If a probe is restricted to a single fixed location (as often occurs with small-molecule ligands), such site-specific constraints could lead to the complete omission of specific interactors like HK1. The ability of our method to switch the catalyst location successfully rescued and validated this novel interactor.

Therefore, rather than merely competing with ligand-based methods, our site-flexible PPL approach serves as a highly versatile and complementary toolkit. We have expanded the Discussion section to explicitly highlight this complementarity, noting how the substantial overlap cross-validates previous findings while our conceptual advances minimize methodological blind spots.

Results and Discussion: (Page 13-14)

"This distinct capture profile, which depends on the modification site, illustrates how local steric environments determine the accessible interactome, highlighting the critical importance of site flexibility. Existing ligand-based PL methods have considerably advanced the mapping of G4 interactomes. However, because these small-molecule ligands inherently bind to specific G4 pockets, they can sterically displace certain native interactors, a phenomenon conceptually similar to the specific masking effect observed with our loop modification toward HK1 and hnRNP A1. Our PPL

approach overcomes this established limitation through its unique site flexibility. Instead of relying on a single binding pocket, our PPL enables the strategic placement of the photocatalyst (e.g., terminal vs. loop). While the terminal probe successfully captured HK1 and hnRNP A1, the loop probe enriched a complementary set of candidates. By combining these site-specific profiles, our PPL reduces the risk of overlooking interactors owing to local microenvironmental constraints. Importantly, the substantial overlap in proteins identified by our PPL method and previous ligand-based approaches validates the reliability of both strategies. Thus, our site-flexible PPL method provides a versatile and complementary toolkit, broadening the technical repertoire for comprehensively mapping complex nucleic acid interactomes without predefined spatial constraints."

3-3: A key limitation is the inability to distinguish direct binders from indirect interactors. The large number of identified candidates suggests limited specificity. The absence of orthogonal validation methods to demonstrate direct binding and of filtering strategies weakens the biological interpretation. Additional analyses, such as STRING network analysis, could help identify interaction patterns and improve data interpretation.

[Answer 3-3] We strongly agree with the reviewer’s insightful point regarding the inherent limitations of proximity labeling. We acknowledge that the inability to strictly distinguish direct from indirect interactors without orthogonal validation is a limitation, and we have explicitly stated this in the revised Discussion. We sincerely thank the reviewer for the excellent suggestion to perform additional network analyses to improve our data interpretation.

To address the concern regarding specificity, we have newly performed comprehensive STRING network and Gene Ontology (GO) enrichment analyses specifically on the candidate proteins identified by our terminal-modified probes (e.g., G4-Ru-term). Strikingly, these analyses revealed that our candidates are not random, non-specific binders, but rather form highly organized and functionally interconnected multiprotein clusters involved in G4-associated nuclear processes, such as DNA replication/repair, chromatin remodeling, and RNA splicing.

Crucially, two major features in this network validate our methodology and highlight its novelty:

1) Inclusion of the positive control: Our positive control, hnRNP A1 (a well-established G4-binding protein), is highly interconnected and perfectly embedded within the massive RNA splicing/processing cluster. This serves as a strong internal validation, confirming that our PPL approach accurately captures the authentic biological interactome surrounding G4 scaffolds in cell extracts.

2) Distinct isolation of HK1: In sharp contrast, Hexokinase-1 (HK1)—which we identified and validated as a novel direct G4-binder—is located notably apart from these major, pre-existing functional clusters. This distinct isolation suggests that HK1 interacts with G4 through a biochemical context distinct from traditional G4-mediated pathways (such as splicing or replication). This accentuates the biological novelty of our discovery, indicating that HK1 may represent a previously uncharacterized functional facet in G4 biology rather than merely an additional component of previously known complexes.

While proximity labeling inherently captures neighboring complex members alongside direct

binders, these network characteristics demonstrate that our method provides a highly specific and unique window into both classical higher-order networks and entirely novel G4 biology. The new STRING and GO analysis data have been incorporated into the revised manuscript (New Supplementary Figure S18 and Supplementary Data 3).

Results and Discussion: (Page 13)

"To gain deeper biological insight and assess the functional connectivity of these identified candidates, we performed STRING network[50] and Gene Ontology (GO) enrichment[51] analyses on the proteins identified by the terminal-modified probes (Supplementary Figure S18 and Supplementary Data 3). The GO analysis revealed significant enrichment in biological processes closely associated with established G4 functions, forming highly interconnected clusters in RNA splicing, DNA replication, and chromatin organization. Notably, the topological distribution of specific proteins within this network provides valuable insights into both the reliability and the novelty of our approach. On one hand, the well-known G4-binding protein hnRNP A1 is robustly integrated into the center of the major RNA splicing cluster, serving as an ideal internal benchmark that confirms the biological relevance of our dataset. On the other hand, consistent with its unique functional background described above, HK1 stands clearly apart from these major, classical macromolecular complexes. This distinct separation suggests that HK1 is involved in a previously uncharacterized, non-canonical G4-mediated pathway that differs from classical G4-associated nuclear processes, thereby emphasizing the novelty of our finding. Consequently, although PL inherently captures broader molecular environments, the ability of our comprehensive PPL method to detect such isolated yet genuine interactors underscores its unique value in expanding the known landscape of G4 biology."

3-4: The study focuses almost exclusively on a single candidate protein (HK1) for validation. While this provides an initial proof of concept, it is not sufficient to validate a global proteomics approach. Validation of additional independent candidates would significantly strengthen the conclusions.

[Answer 3-4] We thank the reviewer for this valuable feedback regarding the validation of our global proteomics approach. We agree that verifying the fidelity of a large-scale interactome dataset is highly important. As suggested by the reviewer in Comment 3-3, we have newly performed a STRING network analysis. We are grateful for this insightful advice, as the resulting network and pathway analyses provide a robust, system-level validation of our quantitative proteomics data.

Specifically, the analyses clearly demonstrate the following: 1) Systemic validation via classical G4 networks: Our dataset successfully captured the well-established positive control hnRNP A1 within the expected major RNA processing cluster. Furthermore, the analysis highlighted the comprehensive enrichment of classical macromolecular machineries intrinsically associated with G4 biology (such as the MCM and FACT complexes). This highly organized functional clustering of known interactors provides a strong internal validation of our method's biological fidelity. 2) Consensus with established platforms: The external validity of our platform is supported by the substantial and meaningful overlap

with the candidate profiles mapped by existing milestone live-cell approaches (G4-LIMCAP and CMPP), confirming the reliability of our covalent, site-flexible labeling strategy.

While our dataset provides a rich reservoir of other uncharacterized candidates, detailed biochemical and functional validation of each protein represents an extensive undertaking that we believe warrants separate future investigations.

Instead, we strategically focused our resources on the deep in vitro validation of HK1. We respectfully note that this focused validation, although centered on a single candidate, was specifically aimed at demonstrating the unique discovery capability of our platform—namely, its ability to identify and authenticate a genuinely novel G4-binding protein not reported by previous methods. HK1 was a candidate that stood distinctly isolated from all classical nuclear clusters in the STRING network (Figure S18), and its novelty is now supported on multiple independent levels:

- Target Specificity: It was significantly enriched alongside the known G4-binder hnRNP A1, whereas the negative-control CPA4 was not (Figure 5).
- Binding Selectivity: Its selective binding to G4 structures over dsDNA was confirmed via EMSA (Figure 7 and Supplementary Figure S20).
- Quantitative Affinity: Its strong binding affinity for HT G4 was rigorously quantified by MST (K_d = 11.8 nM, Figure S21).
- Broad Versatility: It bound a broad panel of physiologically relevant G4 sequences, including the parallel promoter G4s of c-Myc, NRAS, KRAS, and TERRA G4 RNA (Figure S22).

Furthermore, the high reliability of our dataset is highlighted by the fact that several of our identified candidates were independently validated in a recent study (*Commun. Chem.* 2025; ref 10). These results collectively demonstrate that our platform does not merely re-identify known binders but genuinely expands the G4 interactome, paving the way for uncovering new biology. We believe this robust combination of system-level network validation and the comprehensive authentication of a novel target thoroughly addresses the reviewer's concern. We have revised the conclusions section to explicitly highlight these points.

Conclusions: (Page 16-17)

"The reliability of our PPL proteomics approach is strongly supported by both internal and external benchmarks. Internally, the well-organized functional clustering of classical G4 machinery and the successful capture of the positive control (hnRNP A1) provide robust system-level validation. Externally, our identified interactome showed meaningful overlap with candidate profiles mapped by established milestone approaches (e.g., G4-LIMCAP and CMPP), and several of our identified candidates were independently validated in a recent study[10], confirming the reliability of our methodology. Furthermore, although singlet oxygen can diffuse up to ~220 nm in pure water[52], abundant endogenous quenchers in the nuclear extract limit its effective labeling radius to a local macromolecular scale (tens of nanometers). Within this microenvironment, our PPL approach captures both direct G4 binders and indirect complex members. Rather than being a limitation, this capability offers a distinct advantage by enabling comprehensive mapping of the functional, higher-

order interaction networks that dynamically assemble around G4 scaffolds and are often lost in conventional direct-binding assays.

Among the identified candidates, we strategically prioritized the rigorous in vitro validation of HK1. The successful confirmation of HK1—a novel interactor distinctly isolated from classical nuclear complexes—clearly underscores the unique screening capability and discovery potential of our platform. Indeed, its selective binding to G4 DNA/RNA over dsDNA was confirmed via EMSA (Figure 7 and Supplementary Figure S20), and its strong binding affinity (Kd = 11.8 nM) was accurately quantified by microscale thermophoresis (MST) (Supplementary Figure S21)."

3-5: While EMSA is acceptable for qualitative binding assessment, its use for quantitative determination of Kd values is not ideal and may lack accuracy. More robust biophysical techniques (e.g., fluorescence, ITC, MST, or SPR) would strengthen the conclusions.

[Answer 3-5] We agree with the reviewer's valid concern regarding the quantitative accuracy of EMSA. We sincerely thank the reviewer for the excellent suggestion to employ more robust biophysical techniques, which has significantly strengthened our conclusions. Following your constructive advice, we have newly performed microscale thermophoresis (MST) measurements to provide a more accurate and rigorous quantification of the binding affinity of HK1 toward the HT G4. Briefly, FAM-labeled HT G4 DNA (5.0 nM) was titrated with increasing concentrations of HK1 protein in 50 mM Tris-HCl (pH 7.5) containing 100 mM KCl and 0.05% Tween-20. The MST measurements were performed on a Monolith NT.115 instrument (NanoTemper Technologies) at 25 °C. As shown in the new Figure S21, the MST-derived K_d value for the HK1–HT G4 interaction was determined to be 11.8 nM. It should be noted that data points at the highest HK1 concentrations were excluded from the curve fitting, as a sharp decrease in FAM fluorescence intensity was observed in the raw data at these concentrations, indicative of fluorescence quenching. This phenomenon precluded reliable thermophoresis measurements at these concentration ranges, and the remaining data points span from well below to well above the determined K_d, ensuring robust and reliable curve fitting. This value indicates a significantly tighter binding affinity than the initial EMSA-based estimate (apparent K_d = 320 nM). Such discrepancies between MST and EMSA are well documented in the literature and are generally attributed to differences in buffer conditions, equilibrium disruptions during electrophoresis, and caging effects inherent to gel-based assays (König et al., *PLoS ONE*, 2017, doi:10.1371/journal.pone.0178875). Importantly, this highly rigorous MST data strongly aligns with our proteomics results and strongly supports the conclusion that HK1 binds HT G4 with robust affinity and selectivity. In the revised manuscript, we have updated the K_d value reported in the main text to reflect this MST result. The initial EMSA-based result is retained in the Supporting Information for qualitative comparison. Furthermore, the detailed MST protocol and the binding curve (Figure S21) have been added to the revised Supporting Information.

Results and Discussion: (Page 15-16)

"Although EMSA clearly demonstrated the selective binding of HK1 to G4 structures, we performed

microscale thermophoresis (MST) measurements to obtain a more precise quantitative thermodynamic parameter under solution-phase equilibrium conditions. The MST-derived dissociation constant (Kd) for the interaction of HK1 with the FAM-labeled HT G4 DNA was determined to be 11.8 nM (Supplementary Figure S21). This strong binding affinity confirms the specific interaction between HK1 and the G4 structure in solution, further supporting our PPL proteomics results."

3-6: A Gene Ontology analysis should be useful to support the section on "Global profiling of G4 DNA-interacting proteins using PPL" and to provide functional context.

[Answer 3-6] We thank the reviewer for this constructive suggestion. As detailed in our response to Comment 3-3, we have newly conducted comprehensive Gene Ontology (GO) enrichment and STRING network analyses for our identified candidate proteins. The results provided a strong functional context, revealing the substantial involvement of our PPL-identified networks in specific, G4-associated biological processes, particularly DNA replication and repair, chromatin organization, and RNA splicing/processing. These significant findings have been thoroughly incorporated into the Results and Discussion section to improve our biological interpretations.

3-7: Possible inconsistencies between Figures S3 and S4 should be checked and corrected.

[Answer 3-7] We sincerely thank the reviewer for pointing out this oversight. We have carefully reviewed Figures S3 and S4 (New Figures S4 and S5), corrected the inconsistencies, and updated the figures and their corresponding legends in the revised Supporting Information.

Point-by-point responses to the reviewers' comments

Reviewer #1: We sincerely thank Reviewer 1 for the highly positive evaluation of our revised manuscript and the recommendation for publication. We also appreciate the suggestion regarding the recent literature on proximity labeling of G-quadruplexes. As suggested, we have cited the three recent papers (Mo et al., 2026; Barr et al., 2026; de Paepe et al., 2026) in the Introduction section to provide more comprehensive literature coverage.

Introduction: (Page 4) "Other recent proximity labeling approaches have also targeted RNA G4s[33] or utilized G4-binding peptides.[34,35]"

Reviewer #2: We sincerely thank Reviewer 2 for the positive feedback, the thorough evaluation of our revised manuscript, and the recommendation for publication without additional revisions. Your constructive comments during the previous review round significantly improved the quality of our work.

Reviewer #3: We sincerely thank Reviewer 3 for the thorough re-evaluation of our revised manuscript and the highly constructive feedback. The reviewer's insightful comments have been immensely valuable in helping us refine our claims, avoid overinterpretation, and improve the accuracy of our data presentation. We have carefully addressed all the remaining concerns and made the necessary revisions as detailed below.

3-1: The revised manuscript still relies almost exclusively on HK1 as the validation target. While the additional discussion of this limitation is appreciated, validation of a single new candidate is not sufficient to support broad conclusions regarding the fidelity, specificity, and general reliability of the global interactome generated by this approach. Validation of additional independent candidates would substantially strengthen confidence in the screening strategy. This should include both enriched proteins and appropriate non-enriched or low-ranked controls. If additional validation is not feasible, the authors should further temper the general claims regarding the specificity and global reliability of the method.

[Answer 3-1] We sincerely appreciate the reviewer's insightful comment and agree that validating a single new candidate (HK1) cannot, by itself, establish the fidelity, specificity, and reliability of the entire global interactome. In line with the Editor's guidance, we have focused this revision on tempering our claims accordingly.

We note that our validation already spans both directions of the screen: a positive, enriched G4-binding protein (hnRNP A1) and a non-enriched, low-ranked protein (CPA4), which showed no G4 or dsDNA binding by EMSA (Figure 7C). This enriched-versus-non-enriched contrast is consistent with the discriminatory behavior expected of the screen, while we agree it does not replace broader orthogonal validation across many candidates.

Accordingly, we have revised the manuscript to position our PPL approach explicitly as a broad screening platform that generates high-confidence candidate lists requiring further independent validation, and we have removed self-evaluative terms such as "comprehensive" and "reliable" in reference to the global interactome. The specific changes are as follows:

Abstract: (Page 1)

- **Original:** Herein, we report a photocatalytic proximity labeling approach for the **comprehensive identification** of G-quadruplex (G4)-interacting proteins (G4IPs)...
- **Revised:** Herein, we report a photocatalytic proximity labeling approach for the **broad screening** of G-quadruplex (G4)-interacting proteins (G4IPs)...

Results and Discussion: (Page 13)

- **Original:** Notably, the topological distribution of specific proteins within this network provides valuable insights into both the **reliability** and the novelty of our approach. On one hand, the well-known G4-binding protein hnRNP A1 is **robustly** integrated into the center of the major RNA splicing cluster, serving as an **ideal** internal benchmark that **confirms** the biological relevance of our dataset.
- **Revised:** Notably, the topological distribution of specific proteins within this network **is informative regarding** both the **validity** and the novelty of our approach. On one hand, the well-known G4-binding protein hnRNP A1 is integrated into the center of the major RNA splicing cluster, serving as an internal benchmark that **supports** the biological relevance of our dataset.
- **Original: Consequently**, although PL inherently captures broader molecular environments, the ability of our **comprehensive** PPL method to **detect such isolated yet genuine interactors underscores its unique value in** expanding the known landscape of G4 biology.
- **Revised: Overall**, although PL inherently captures broader molecular environments, the ability of our PPL method to **surface such candidates outside the classical clusters highlights its potential as a discovery tool for** expanding the known landscape of G4 biology.

Conclusions: (Page 17)

- **Original:** The **reliability** of our PPL proteomics approach is strongly supported by both internal and external benchmarks.
- **Revised:** The **utility** of our PPL proteomics approach **as a screening platform** is strongly supported by both internal and external benchmarks.
- **Original:** Externally, our identified interactome showed meaningful overlap with candidate profiles mapped by established milestone approaches (e.g., G4-LIMCAP and CMPP), **confirming the reliability of our methodology**.
- **Revised:** Externally, our identified interactome showed meaningful overlap with candidate profiles mapped by established milestone approaches (e.g., G4-LIMCAP and CMPP), **supporting the validity of our methodology**.

- **Original:** Rather than being a limitation, this capability offers a distinct advantage by **enabling comprehensive mapping** of the functional, higher-order interaction networks...
- **Revised:** Rather than being a limitation, this capability offers a distinct advantage by **enabling broader mapping** of the functional, higher-order interaction networks...

(Limitation explicitly added in Conclusions)

- **Original:** (None)
- **Revised (Added): "Nevertheless, we acknowledge that, while our PPL platform successfully generates high-confidence candidate lists, the specificity and global reliability of the resulting interactome remain to be established, and individual candidates will require further orthogonal validation in future studies."**

3-2: Related to this point, parts of the revised discussion still appear to overinterpret individual candidates or network-analysis results. The STRING analysis provides useful functional context, but it does not establish direct G4 binding or demonstrate that specific candidates represent distinct biological pathways or mechanistic classes. In particular, the observed separation of HK1 in the STRING network does not by itself demonstrate a distinct mechanistic role for HK1 in G4 biology. This interpretation should therefore be presented more cautiously.

[Answer 3-2] We appreciate the reviewer's careful reading and agree that our previous wording was overstated. STRING analysis relies on existing functional annotations and does not, by itself, establish direct G4 binding or distinct biological pathways. We have carefully reviewed all discussion points derived from the STRING analysis and revised them to present functional clustering as suggestive context rather than mechanistic evidence.

Regarding HK1, we have revised the discussion to avoid mechanistic overinterpretation while maintaining the logical transition to our biochemical validation. We have removed the speculative statement regarding a "non-canonical G4-mediated pathway," and now clarify that the network separation reflects a lack of previously documented functional links. It was this absence of prior annotation that motivated our decision to test HK1's G4 binding directly, which the subsequent EMSA and MST data then confirmed. The specific changes are as follows:

Results and Discussion: (Page 13)

- **Original:** ...HK1 stands clearly apart from these major, classical macromolecular complexes. **This distinct separation suggests that HK1 is involved in a previously uncharacterized, non-canonical G4-mediated pathway that differs from classical G4-associated nuclear processes, thereby emphasizing the novelty of our finding.**
- **Revised:** ...HK1 stands clearly apart from these major, classical macromolecular complexes. **Because STRING reflects existing annotations, this separation highlights a lack of previously reported functional links. This lack of prior annotation made HK1 a highly attractive candidate, providing strong motivation to directly test its G4 binding.**

3-3: The addition of MST is appreciated. However, quantitative affinity measurements were performed only for HT G4, whereas the manuscript extends the interpretation toward broader G4 recognition by HK1. Because EMSA experiments were expanded to multiple G4 structures, it would be more convincing to provide quantitative binding measurements for at least selected additional G4 targets rather than relying exclusively on qualitative EMSA assessment. If this is not possible, the claims regarding broader G4 recognition should be clearly described as qualitative.

[Answer 3-3] We thank the reviewer for this highly constructive feedback. We completely agree that while our EMSA data across the broader G4 panel clearly demonstrate selective binding, they remain qualitative regarding relative affinities. As requested, we have explicitly revised the text to state that the topology preference observed via EMSA represents a qualitative trend.

Concurrently, we have reframed our Conclusions so that the MST measurement for HT G4 serves as a quantitative reference point of the study. Rather than resting broader claims solely on the qualitative EMSA panel, we now position the K_d value—together with the raw traces and capillary scans newly provided in the Supporting Information (see Answer 3-4)—as a foundation for future studies on its G4 topology preferences. The specific changes are as follows:

Results and Discussion: (Page 16)

- **Original:** HK1 bound to both DNA and RNA G4s **and showed a preference for NRAS G4 RNA.**
- **Revised:** HK1 bound to both DNA and RNA G4s**, showing the most prominent shift for NRAS G4 RNA. We note that this EMSA-based topology preference is a qualitative trend, and defining its quantitative basis is a natural next step opened up by these results.**

Conclusions: (Page 17)

- **Original:** Indeed, its selective binding to **G4 DNA/RNA over dsDNA was confirmed via EMSA (Figure 7 and Supplementary Figure S20), and its strong binding affinity (K_d = 11.8 nM) was accurately quantified by microscale thermophoresis (MST) (Supplementary Figure S21).**
- **Revised:** Indeed, **HK1 binds selectively to a range of G4 DNA/RNA structures over dsDNA (EMSA, Figure 7 and Supplementary Figure S22), and binds HT G4 with high affinity (K_d = 11.8 nM by MST, Supplementary Figures S21)—demonstrating that HK1 is a high-affinity G4-binding protein as quantified for HT G4 and providing a foundation for future studies on its G4 topology preferences.**

3-4: Furthermore, the substantial discrepancy between the original EMSA-derived apparent Kd and the newly reported MST Kd deserves more careful discussion, particularly considering that high-concentration data points were excluded from MST fitting. Additionally, I noticed an internal inconsistency that should be clarified before the affinity data can be properly evaluated. In the rebuttal and revised Results section, the MST experiment is described as

being performed using FAM-labeled HT G4 DNA. However, the Supporting Information (Methods and Figure S21 legend) describes the MST experiment as being performed using Texas Red-labeled HT G4 at 20 nM. Moreover, the inconsistency appears to involve not only the fluorophore and DNA concentration, but also the buffer composition and KCl concentration, which are particularly relevant for G4 folding and MST-derived affinity estimates. Please clarify which fluorescently labeled construct was actually used (FAM or Texas Red), which DNA concentration and buffer conditions were applied, and whether the reported Kd = 11.8 nM originates from the same experiment described in the Supplementary Information. Because fluorophore identity, DNA concentration, and buffer composition can influence fluorescence behavior, G4 folding, thermophoretic response, and apparent affinity estimates in MST experiments, especially in the presence of reported fluorescence quenching at high protein concentrations, this inconsistency should be corrected and reconciled throughout the manuscript and Supporting Information. Finally, because the reported affinity is substantially stronger than the EMSA-derived estimate, the robustness of the fit becomes especially important. To allow independent assessment of data quality, I suggest providing also the raw MST traces and capillary fluorescence data.

[Answer 3-4] We sincerely apologize for the confusion caused by the inconsistencies regarding the MST experimental details. To clarify, the correct experimental conditions that yielded the reported K_d of 11.8 nM are as follows: the experiment was performed using Texas Red-labeled HT G4 DNA at a concentration of 20 nM, in MST assay buffer (10 mM Tris-HCl, pH 7.5, 20 mM KCl, 0.05% Tween-20 to prevent non-specific adsorption to capillary surfaces). We confirm that the reported K_d = 11.8 ± 2.0 nM (mean ± SD) was determined from three independent experiments performed under identical conditions, and that all descriptions in the main text and the Supporting Information have been thoroughly corrected and reconciled.

Regarding the discrepancy between the apparent K_d derived from EMSA (320 ± 49 nM) and the MST-derived K_d (11.8 ± 2.0 nM), we have added a clarifying discussion in the revised text. This difference is expected from a methodological standpoint. EMSA is a non-equilibrium, gel-based assay in which complex dissociation during electrophoresis often leads to an underestimation of binding affinity (i.e., a higher apparent K_d). In contrast, MST directly measures the interaction in free solution under true equilibrium conditions. We therefore consider the MST-derived value to more accurately represent the in-solution affinity, while noting that the two values should be regarded as complementary rather than directly comparable.

The lower KCl concentration used in the MST assay buffer (20 mM) relative to the EMSA buffer (100 mM) is not expected to compromise G4 folding. Human telomeric G4 DNA has been shown to fold with a midpoint near 0.3 mM KCl, reaching >95% folding at 5 mM KCl (Gray & Chaires, *Biophys. Chem.* 2011, 159, 205–209). Thus, 20 mM KCl is well above the concentration required for complete G4 formation. The observed difference in KD values is therefore more likely attributable to the ionic strength dependence of protein–nucleic acid interactions, whereby higher salt concentrations shield

electrostatic interactions and reduce apparent binding affinity.

Regarding the exclusion of high-concentration data points, data points at HK1 concentrations ≥400 nM (the four highest concentrations tested) were excluded from fitting. At these concentrations, a consistent decrease in both the capillary fluorescence intensity and the Fnorm values was observed, indicating fluorescence quenching of Texas Red by HK1 at high protein concentrations rather than a true thermophoretic response. The remaining 12 data points, spanning concentrations below and above the MST-derived K_d, were used for fitting and yielded a well-defined binding curve, supporting the reliability of the reported K_d value. As requested, we have provided the raw MST traces, the capillary fluorescence scans, and a representative binding curve (K_d= 11.8 ± 2.0 nM) as Supplementary Figure S21.

Results and Discussion: (Page 14-15)

- **Original:** Although EMSA clearly demonstrated the selective binding of HK1 to G4 structures, we performed microscale thermophoresis (MST) measurements to obtain a more precise quantitative thermodynamic parameter under solution-phase equilibrium conditions. The MST-derived dissociation constant (K_d) for the interaction of HK1 with the FAM-labeled HT G4 DNA was determined to be 11.8 nM (Supplementary Figure S21).
- **Revised:** Although EMSA clearly demonstrated the selective binding of HK1 to G4 structures, we performed microscale thermophoresis (MST) measurements to obtain a more precise quantitative thermodynamic parameter under solution-phase equilibrium conditions. The MST-derived dissociation constant (K_d) for the interaction of HK1 **with Texas Red-labeled HT G4 DNA (20 nM, in 10 mM Tris-HCl (pH 7.5) containing 20 mM KCl and 0.05% Tween-20) was determined to be 11.8 ± 2.0 nM (n = 3; Supplementary Figure S21). It should be noted that this MST-derived affinity is substantially stronger than the apparent K_d estimated from EMSA (320 ± 49 nM). This discrepancy reflects methodological differences: EMSA is a non-equilibrium assay in which complex dissociation during electrophoresis often leads to an underestimation of affinity, whereas MST measures the interaction in free solution under true equilibrium conditions. Additionally, the higher KCl concentration used in the EMSA buffer (100 mM) relative to the MST assay buffer (20 mM) is expected to attenuate electrostatic protein–DNA interactions, contributing to the weaker apparent affinity observed by EMSA. Data points at or above 400 nM (≥400 nM) HK1 were excluded from the fitting owing to fluorescence quenching of Texas Red by HK1 at high protein concentrations, as evidenced by a consistent decrease in both the capillary fluorescence intensity and Fnorm values; the raw MST traces and capillary fluorescence data are provided in Supplementary Figure S21 to allow independent assessment of data quality and fitting robustness.**

Supporting Information: (Methods – Section 1.16)

- **Original:** ...The normalized fluorescence change (Fnorm) was plotted against HK1 concentration, and the equilibrium dissociation constant (K_d) was determined by fitting data to a standard 1:1 binding model using the equation: ΔFnorm = ΔFnorm,max × [HK1] / (K_d + [HK1])
- **Revised:** ...The normalized fluorescence change (Fnorm) was plotted against HK1 concentration, and the equilibrium dissociation constant (K_d) was determined by fitting data to a standard 1:1 binding model using the equation: ΔFnorm = ΔFnorm,max × [HK1] / (K_d + [HK1])

  Data points at HK1 concentrations ≥400 nM (the four highest concentrations tested) were excluded from fitting owing to fluorescence quenching of Texas Red by HK1 at high protein concentrations, as evidenced by a consistent decrease in both the capillary fluorescence intensity and Fnorm values.

Figure legend: (**Figure S21**)

- **Original:** Figure S21. Microscale thermophoresis (MST) determination of the binding affinity of HK1 for G4. Binding of HK1 to the human telomeric G4 sequence (HT G4; 5′-d(TAGGGTTAGGGTTAGGGTTAGGG)-3′) labeled at the 3′ end with Texas Red (HT-Texas Red, 20 nM) was measured by MST. HK1 was serially diluted in MST assay buffer (10 mM Tris-HCl, pH 7.5, 20 mM KCl, 0.05% Tween-20) and titrated from 3.2 μM to 0.1 nM (16 two-fold dilutions). The normalized fluorescence (Fnorm) is plotted as a function of HK1 concentration. The data were fitted to a standard 1:1 binding model.
- **Revised:** Figure S21. Microscale thermophoresis (MST) determination of the binding affinity of HK1 for G4. Binding of HK1 to the human telomeric G4 sequence (HT G4; 5′-d(TAGGGTTAGGGTTAGGGTTAGGG)-3′) labeled at the 3′ end with Texas Red (HT-Texas Red, 20 nM) was measured by MST. HK1 was serially diluted in MST assay buffer (10 mM Tris-HCl, pH 7.5, 20 mM KCl, 0.05% Tween-20) and titrated from 3.2 μM to 0.1 nM (16 two-fold dilutions). (A) Capillary fluorescence scan showing the initial fluorescence intensity across all 16 capillaries. (B) Raw MST traces. (C) The normalized fluorescence (Fnorm) is plotted as a function of HK1 concentration. The data were fitted to a standard 1:1 binding model. Data points at HK1 concentrations ≥400 nM were excluded from fitting owing to fluorescence quenching of Texas Red at high protein concentrations, as evidenced by a consistent decrease in both the capillary fluorescence intensity and Fnorm values. The reported K_d = 11.8 ± 2.0 nM represents the mean ± SD from three independent experiments.
