# Supplementary material for: Photocatalytic proximity labeling for the identification of G-quadruplex DNA-interacting proteins
Source: Commun Chem. 2026 Aug 27;9:286. doi: 10.1038/s42004-026-02170-9 (PMC13522520; doi:10.1038/s42004-026-02170-9)

Supplementary Information for

Photocatalytic proximity labeling for the identification of G-quadruplex DNA-interacting proteins

Ahmed Mostafa Abdelhady[1,2,3], Shinichi Sato[4*], Tatsuki Masuzawa[5], Keishi Deguchi[5], Mizuki Oba[5], Takemaru Sato[5], Nodoka Mase[5], Toshifumi Yamanaka[5], Jamila Abbas Osman[1,2], Maho Kato[1,2], Keita Nakane[4], Zhengyi Liu[4], Kazuki Kuwahara[1,2], Satoru Nagatoishi[6], Kouhei Tsumoto[6], Fumi Nagatsugi[1,2], Takanori Oyoshi[5,7,8*], Kazumitsu Onizuka[1,2,9*]

[1] Institute of Multidisciplinary Research for Advanced Materials, Tohoku University, 2-1-1 Katahira, Aoba-ku, Sendai, Miyagi 980-8577, Japan.

[2] Department of Chemistry, Graduate School of Science, Tohoku University, Aoba-ku, Sendai, Miyagi 980-8578, Japan.

[3] Department of Chemistry, Faculty of Science, Al-Azhar University, Nasr City, 11884 Cairo, Egypt.

[4] Frontier Research Institute for Interdisciplinary Sciences and Graduate School of Life Sciences, Tohoku University, Aoba-ku, Sendai, Miyagi 980-8578, Japan.

[5] Graduate School of Integrated Science and Technology, Shizuoka University, 836 Ohya, Suruga-ku, Shizuoka, 422-8529, Japan.

[6] Department of Bioengineering, School of Engineering, The University of Tokyo, Tokyo, Japan.

[7] Graduate School of Science and Technology, Shizuoka University, 836 Ohya, Suruga-ku, Shizuoka, 422-8529, Japan.

[8] Research Institute of Green Science and Technology, Shizuoka University, 836 Ohya, Suruga-ku, Shizuoka, 422-8529, Japan.

[9] Division for the Establishment of Frontier Sciences of Organization for Advanced Studies, Tohoku University, Aoba-ku, Sendai, Miyagi 980-8577, Japan.

List of Contents:

	Content	Page
**1.**	**Experimental data**	**2**
**1.1.**	**General Materials and methods**	**2**
**1.2.**	**MAUra-N_3 synthesis (7)**	**2**
**1.3.**	**MAUra-DTB synthesis (10)**	**4**
**1.4.**	**Preparation of G4-NH_2-loop**	**5**
**1.5.**	**Photocatalyst conjugation for G4-cat-loop, G4-cat-term, or ss-cat**	**5**
**1.6.**	**MALDI-TOF/MS measurements data for DNA-cat conjugates**	**6**
**1.7.**	**CD measurements**	**6**
**1.8.**	**Native PAGE analysis for cat-DNA**	**7**
**1.9.**	**Melting temperature (T_m) measurement (CD)**	**7**
**1.10.**	**Melting temperature (T_m) measurement (UV-vis)**	**7**
**1.11.**	**Fluorescence measurement for G4-cat**	**7**
**1.12.**	**UP1 labeling with sodium azide**	**8**
**1.13.**	**CD spectroscopy before and after photo-irradiation**	**8**
**1.14.**	**EMSA-based assessment of protein binding after photo-irradiation**	**9**

**1.15.**	**Protein-Protein Interaction (PPI) Network Analysis.**	**9**
**1.16.**	**Microscale thermophoresis (MST) measurement**	**9**
**2.**	**Additional data and figures**	**10**
**3.**	**NMR-spectra**	**30**
**4.**	**MALDI-TOF MS data**	**40**

1. Experimental data

1.1. General Materials and methods. The general chemicals were purchased from commercial suppliers (FUJIFILM Wako Pure Chemical, the Tokyo Chemical Industry, Kanto Chemical, Nacalai tesque, or Aldrich) and used without further purification unless specifically mentioned. The target DNAs and RNAs were purchased from JBioS (Japan). The ^{1}H NMR spectra (400 MHz) were recorded by a Bruker AVANCE III 400 spectrometer. The ^{1}H NMR spectra (600 MHz) and ^{13}C NMR spectra (150 MHz) were recorded by a Bruker AVANCE III 600 spectrometer. The high-resolution electrospray mass analysis was performed by a Bruker MicrOTOFQ II. The HPLC purification was performed by a JASCO HPLC System (PU-4180, UV-4075, and CO-2067Plus). The MALDI-TOF MS measurements were performed by a Bruker Autoflex Speed instrument utilizing a 3-hydroxypicolinic acid/diammonium hydrogen citrate matrix.

1.2. 1-methyl-4-aryl-urazole-azide (MAUra-N_3) synthesis (7). 4-((6-azidohexyl)oxy)aniline **4** was prepared according to the previously reported procedure.

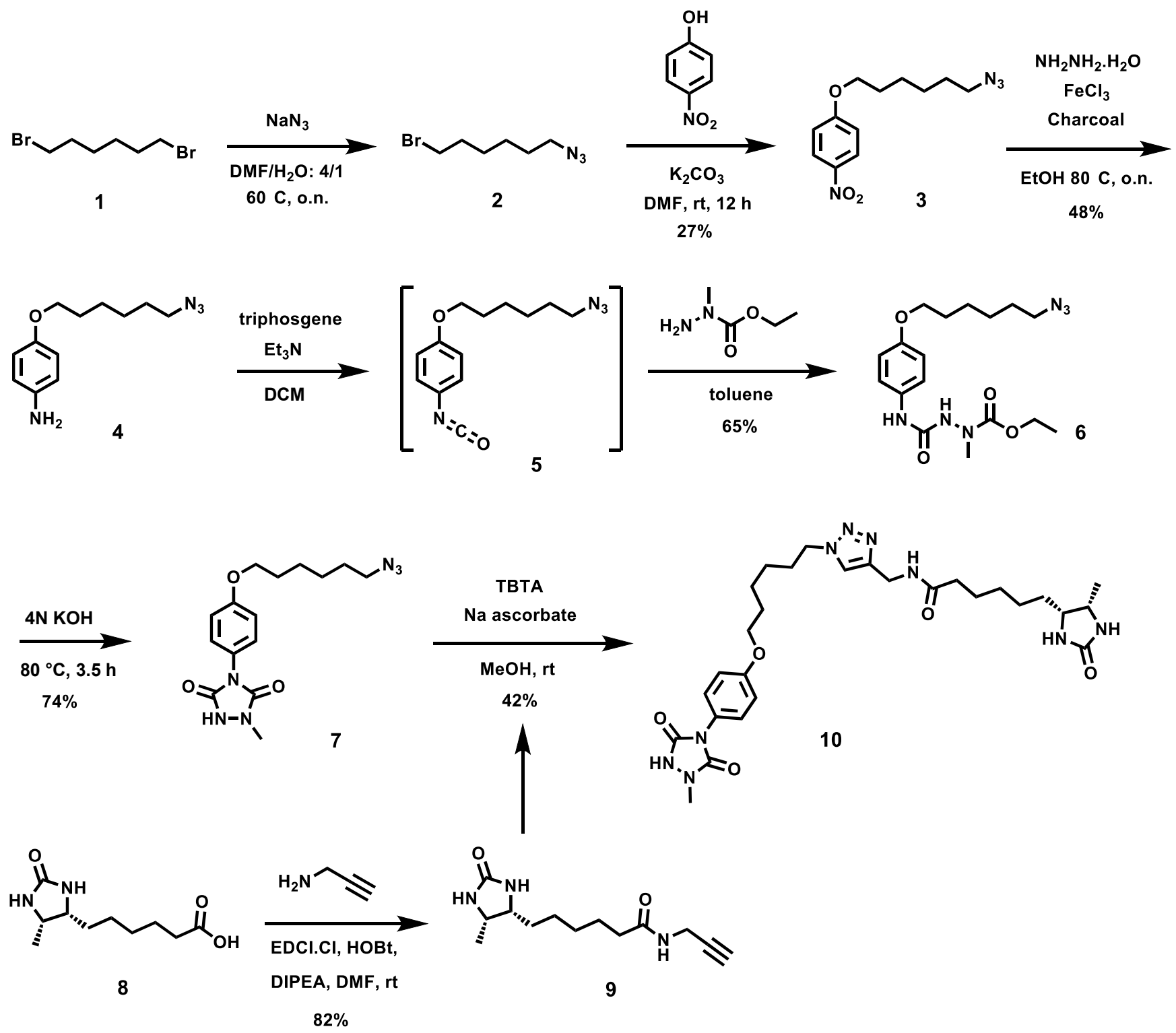

1.2.1. 1-azido-6-bromohexane (2). Sodium azide (1.88 g, 29.0 mmol) was added to the solution of 1,6-dibromohexane **1** (6.42 g, 26.3 mmol) in the mixture of *N,N*-dimethylformamide (28.8 mL), and H_2O (9.6 mL). The mixture was heated at 60 °C under an argon atmosphere. After 17 hours, the reaction mixture was cooled to room temperature and diluted with dichloromethane (50 mL). The organic layer was washed with H_2O (50 mL×3) and brine (50 mL×1), dried over Na_2SO_4, and evaporated under vacuum to obtain **2**. The crude was used for the subsequent reaction without further purification.

^{1}H NMR (600 MHz, $CDCl_3$) δ 1.47-1.48 (2H, m), 1.48-1.49 (2H, m), 1.59-1.64 (2H, m), 1.85-1.90 (2H, m), 3.28 (2H, t, J = 6.6 Hz), 3.42 (2H, t, J = 6.6 Hz).

1.2.2. 1-((6-azidohexyl)oxy)-4-nitrobenzene (3). 4-Nitrophenol (1.14 g, 8.19 mmol) and potassium carbonate (1.71 g, 12.4 mmol) were combined with the solution of **2** (2.03 g, 9.84 mmol) in *N,N*-dimethylformamide (10 mL) and then stirred at room temperature under argon atmosphere. After 22 h, the reaction mixture was diluted with ethyl acetate (50 mL) and washed with H_2O (50 mL ×3) and brine (50 mL ×3), then dried over Na_2SO_4 and evaporated. The crude was purified by flash silica gel chromatography (hexane only to hexane/ethyl acetate = 12/1 → 8:1 → 2:1 → 0/1) to afford the desired product (750 mg, yield = 27%) as a pale-yellow oil.

^{1}H NMR (400 MHz, $CDCl_3$) δ 1.41-1.56 (4H, m), 1.62-1.68 (2H, m), 1.81-1.92 (2H, m), 3.30 (2H, t, J = 5.6 Hz), 4.06 (2H, t, J = 6.4 Hz), 6.94 (2H, d, J = 12.8 Hz), 8.20 (2H, d, J = 9.2 Hz). HRMS (ESI): *m/z* calcd. for $C_{12}H_{17}N_4O_3^+$ $[M+H]^+$, 265.1295; found, 265.1301.

1.2.3. 4-((6-azidohexyl)oxy)aniline (4). Compound **3** (572 mg, 2.16 mmol) in tetrahydrofuran/ethanol (6.3 mL/19.1 mL) was mixed with charcoal (292.5 mg), $FeCl_3.6H_2O$ (58.5 mg, 0.22 mmol), and hydrazine monohydrate (8.60 g, 173 mmol). The mixture was stirred for 24 h at 80 °C, then cooled to room temperature, followed by celite filtration. The solvent was evaporated under reduced pressure. The residue was dissolved into ethyl acetate, washed with H_2O (100 mL ×2) and brine (100 mL ×1), and dried over Na_2SO_4. The residue was concentrated under reduced pressure and purified by a silica gel column (ethyl acetate/hexane: 1/8→1/3) to afford **4** as a pale brown oil (242 mg, 48%).

^{1}H NMR (600 MHz, $CDCl_3$): δ 6.74-6.72 (m, 2H), 6.65-6.63 (m, 2H), 3.88 (t, J = 6.6 Hz, 2H), 3.28 (t, J = 6.6 Hz, 2H), 1.78-1.73 (m, 2H), 1.66-1.61 (m, 2H), 1.51-1.41 (m, 4H).
$^{13}C\{^1H\}$ (150 MHz; $CDCl_3$) δ 152.3, 139.9, 116.4, 115.7, 68.4, 51.4, 29.3, 28.8, 26.5, 25.7.
HRMS (ESI): *m/z* calcd. for $C_{12}H_{19}N_4O^+$ $[M+H]^+$, 235.1553; found, 235.1550.

1.2.4. MAUra-N_3 precursor (6). A triphosgene (27.6 mg, 0.093 mmol) in dichloromethane (0.12 mL) was mixed dropwise with compound **4** (21.8 mg, 0.093 mmol) in dichloromethane (0.2 mL) on ice followed by triethylamine (39.2 µL, 0.279 mmol) dropwise. Stirring commenced for 1h at room temperature. The reaction mixture was diluted with dichloromethane (5 mL) and washed with brine (5 mL, ×1). The organic layer was dried over Na_2SO_4 and dried under reduced pressure to give intermediate **5**. The intermediate in toluene (0.31 mL) was mixed with ethyl-1-methyl hydrazine carboxylate (9.9 mg, 0.084 mmol). The mixture was stirred for 1 h at room temperature and then for 3.5 h at 90 °C, cooled to room temperature, quenched with methanol (0.5 mL), and dried under reduced pressure. Purification by silica gel column (ethyl acetate/hexane: 1/1) afforded **6** (23 mg, two steps 65%) as a white solid.

1.2.5. MAUra-N_3 (7). Compound **6** (23 mg, 0.06 mmol) in 4 N KOH aq. (1 mL) was stirred for 3.5 h at 80 °C, cooled to room temperature, and acidified by 5.5 N HCl (0.7 mL) to reach pH 1.0. The product was extracted by dichloromethane (20 mL ×2), dried over Na_2SO_4, and the solvent evaporated under reduced pressure to obtain compound **7** as a pale pink solid (17 mg, 74%).

^{1}H NMR (600 MHz, $CDCl_3$): δ 7.36 (d, J = 9.2 Hz, 2H), 6.97 (d, J = 9.2 Hz, 2H), 3.98 (t, J = 6.6 Hz, 2H), 3.29 (t, J = 6.6 Hz, 2H), 3.25 (s, 3H), 1.83-1.78 (m, 2H), 1.67-1.62 (m, 2H), 1.53-1.44 (m, 4H). ^{13}C{^{1}H} (150 MHz; $CDCl_3$) δ 159.0, 154.2, 153.9, 127.2, 123.5, 115.1, 68.0, 51.4, 34.0, 29.0, 28.8, 26.5, 25.6. HRMS (ESI): *m/z* calcd. for $C_{15}H_{19}N_6O_3$ $[M-H]^-$, 331.1513; found 331.1538.

1.3. 1-methyl-4-aryl-urazole-desthiobiotin conjugate (MAUra-DTB) synthesis (10):

1.3.1. Alkynylated desthiobiotin (9). DIPEA (489 µL, 2.8 mmol), HOBt.H_2O (126.1 mg, 0.933 mmol), EDCI.HCl (357.7 mg, 1.866 mmol) and propargylamine (92 µL, 1.4 mmol) were added to *d*-desthiobiotin (200 mg, 0.93 mmol) in *N,N*-dimethylformamide (3.28 mL) and stirred at room temperature. After 26 h, the solvent was removed *in vacuo*. The residue was purified by a silica gel column (chloroform/methanol: 100/1→100/2), yielding **9** as a pale-yellow foam. Yield = 191 mg, 82% (calculated from ^{1}H NMR).

^{1}H NMR (400 MHz, CD_3OD): δ 3.95 (d, 2H), 3.85-3.78 (m, 1H), 3.72-3.67 (m, 1H), 2.57 (t, J = 2.4 Hz, 1H), 2.20 (t, J = 7.6, 2H), 1.67-1.6 (m, 2H), 1.52-1.29 (m, 6H), 1.10 (d, 3H).

1.3.2. MAUra-desthiobiotin conjugate (10). To a solution of MAUra-N_3 **7** (18.7 mg, 56.3 µmol) in MeOH (1.15 mL), *d*-desthiobiotin derivative **9** (14.8 mg, 59.1 µmol), TBTA (14.9 mg, 28.1 µmol), $CuSO_4.5H_2O_{aq.}$ (4.9 mg, 20 µmol, 175 µL) and L(+)-sodium ascorbate (11.5 mg) were added and stirred at room temperature. After 17 h, the solvent was removed *in vacuo*. The residue was purified by PTLC (chloroform/methanol: 4/1) followed by HPLC (5-70% acetonitrile/H_2O), yielding MAUra-desthiobiotin conjugate as a white solid (13.7 mg, 42%).

^1H NMR (600 MHz, CD_3OD): δ 7.84 (s, 1H), 7.30 (d, 2H), 6.89 (d, 2H), 4.41-4.38 (m, 4H), 3.99 (t, J = 6.6 Hz, 2H), 3.82-3.77 (m, 1H), 3.69-3.65 (m, 1H), 3.24 (s, 3H), 2.22 (t, J = 7.2 Hz, 2H), 1.95-1.90 (m, 2H), 1.80-1.75 (m, 2H), 1.65-1.60 (m, 2H), 1.55-1.10 (m, 10H), 1.09 (d, 3H). $^{13}C\{^1H\}$ (150 MHz; CD_3OD) δ 174.7, 164.8, 158.5, 154.9, 152.8, 144.8, 127.7, 125.5, 122.8, 114.4, 67.6, 55.9, 51.3, 49.9, 35.3, 34.2, 30.6, 29.8, 29.3, 28.8, 28.7, 25.8, 25.7, 25.3, 25.1, 14.2. HRMS (ESI): *m/z* calcd. for $C_{28}H_{42}N_9O_5^+[M+H]^+$,584.3309; found, 584.3304.

1.4. Preparation of DNA oligonucleotide (G4-NH_2-loop). Amino group-bearing modified thymine phosphoramidite, which was commercially available, was incorporated into the HT DNA sequence. The DNA oligonucleotide was synthesized according to standard protocols (1.0 µmol scale) utilizing an Applied Biosystems model 392 DNA/RNA synthesizer. Deprotection and cleavage from the CPG support were carried out under ultra-mild conditions with a 28% ammonia solution at room temperature for 4 h. Next, the DMTr-ON oligonucleotide was purified by HPLC. The HPLC purification conditions were as follows: C18 column (Nacalai tesque: COSMOSIL $5C_{18}$-MS-II, 4.6ID x 250 mm) by a linear gradient of 10-40%/20 min acetonitrile in TEAA buffer (0.1 M) at the flow rate of 4 mL/min and 35 °C. Peaks were monitored by a UV detector (λ = 254 nm). Subsequently, the DMTr group was deprotected by 10% acetic acid at room temperature for 1 h. The oligonucleotide was then purified by HPLC using the same conditions mentioned above. The concentration of the ODN was calculated by UV absorption at 260 nm.

1.5. Photocatalyst conjugation for G4-cat-loop, G4-cat-term, or ss-cat. ODN-NH_2 (500 µM) in sodium borate buffer (100 mM, pH 8.7) was incubated with BDP FL NHS ester (10 mM, in DMSO) or $Ru(bpy)_2$(mcbpy-O-Su-ester)$(PF_6)_2$ (12.5 mM, in DMSO) at room temperature for 15 min in a dark room. The photocatalyst-oligonucleotide conjugate was purified by HPLC. The HPLC purification conditions were as follows: C-18 column (Nacalai tesque: COSMOSIL $5C_{18}$-MS-II, 4.6ID × 250 mm) by a linear gradient of 5-30%/20 min acetonitrile in TEAA buffer (0.1 M) at the flow rate of 1 mL/min and 35 °C. Peaks were monitored by a UV detector (λ = 254 nm). To remove Ru complex NHS ester reagent altogether,

Ru complex-modified ODN was further purified by polyacrylamide gel electrophoresis (PAGE). The sample was mixed with loading buffer (2×, 95% formamide, 50 mM EDTA pH 8.0). PAGE was performed by a 16% polyacrylamide and 20% formamide with 1X TBE and 7.5 M urea at 400 V for 90 min. The yielding product was purified again by HPLC, and molecular weight was then checked by MALDI-TOF MS.

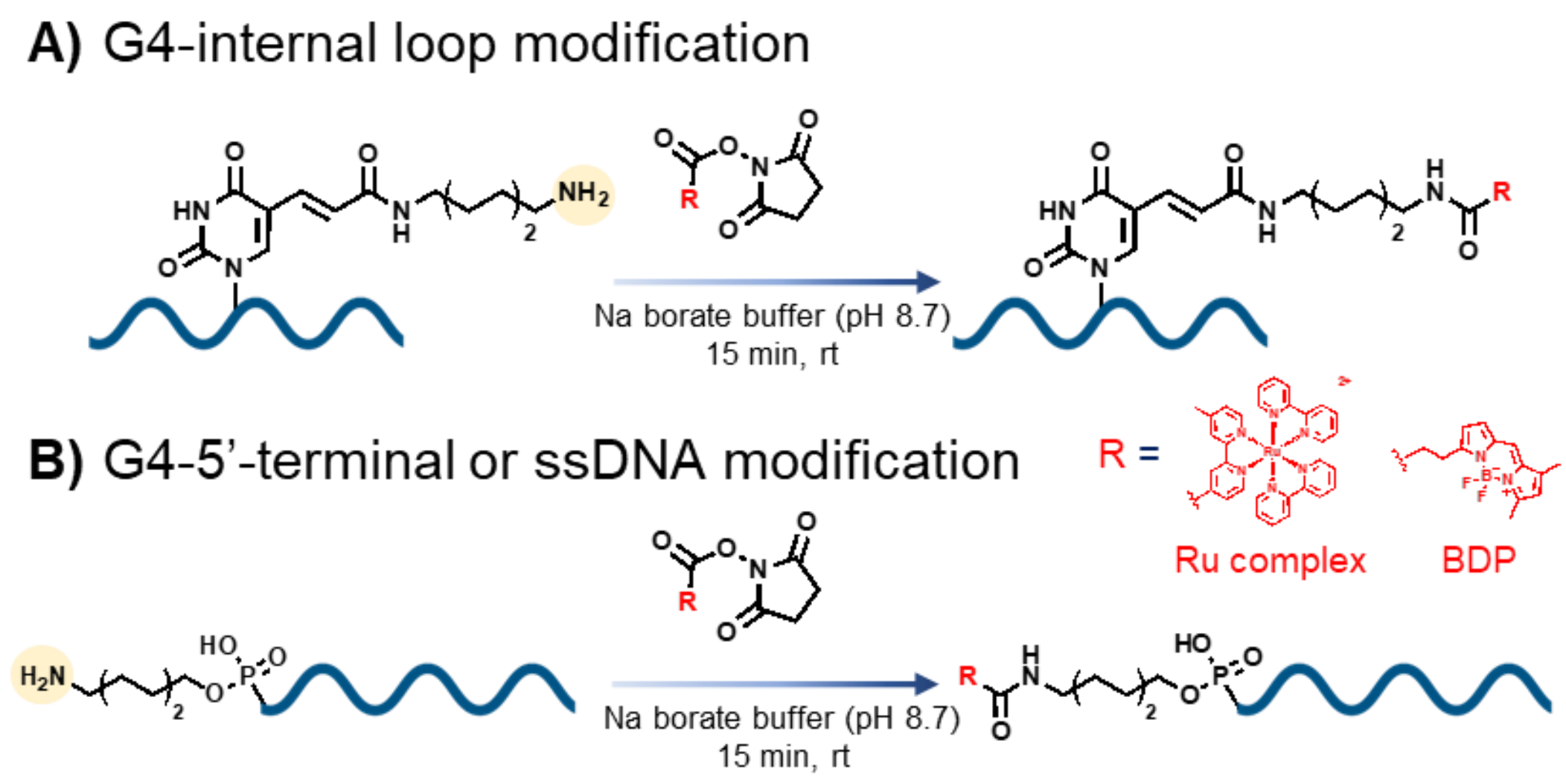

Scheme S1. Synthetic scheme of the photocatalyst-conjugated DNA. The catalyst modification for A) G4-cat-loop and B) G4-cat-term and ss-cat.

1.6. MALDI-TOF/MS measurements data for DNA-cat conjugates.

Oligomer	Sequence (5' to 3')	MALDI-TOF MS	
		Calcd. [a]	Found[b]
G4-Ru-loop	TA**GGG**TTA**GGG**T[Ru]TA**GGG**TTA**GGG**	8030.4	8030.7
G4-Ru-term	**Ru**-TA**GGG**TTA**GGG**TTA**GGG**TTA**GGG**	8055.3	8055.7
ss-Ru	**Ru**-CTGAGTGAGTGAGTGAGA	6429.1	6429.5
G4-BDP-loop	TA**GGG**TTA**GGG**T[BDP]TA**GGG**TTA**GGG**	7694.4	7695.5
G4-BDP-term	**BDP**-TA**GGG**TTA**GGG**TTA**GGG**TTA**GGG**	7719.3	7719.9
ss-BDP	**BDP**-CTGAGTGAGTGAGTGAGA	6093.1	6093.5

MALDI-TOF/MS measurement of the catalyst-modified ONs. [a] $[M-H]^-$, [b] all data were collected in negative mode.

1.7. CD measurements. A solution (100 μL) of the photocatalyst-G4 DNA conjugate (5.0 μM) in 50 mM Tris-HCl buffer (pH 7.5) containing 100 mM KCl was annealed by incubation at 90 °C, followed by cooling to 4 °C at a rate of 0.5 °C/min. The mixture was transferred to a micro quartz cell with a 1-cm path length. The CD spectra were measured at 20 °C by a J-720WI (JASCO Corporation) equipped with a Peltier temperature controller.

1.8. Native PAGE analysis for cat-DNA. To assess the overall G4 fold of the photocatalyst-modified HT DNA sequences, native polyacrylamide gel electrophoresis (native PAGE) was performed. A solution of each G4 sequence (G4-Ru-loop, G4-Ru-term, G4-BDP-loop, G4-BDP-term, and unmodified native HT G4; 0.1 μM each) in 50 mM Tris-HCl buffer (pH 7.5) containing 100 mM KCl was annealed by incubation at 90 °C, followed by slow cooling to 4 °C at a rate of 0.5 °C/min. Each sample was mixed with 50% glycerol. Electrophoresis was performed using a 12% non-denaturing polyacrylamide gel (19:1 acrylamide:bis-acrylamide) prepared in 0.5× TBE supplemented with 50 mM KCl. After loading, electrophoresis was performed at 4 °C and 120 V for 60 min. The gel was stained with SYBR Gold (Thermo Fisher Scientific), and fluorescence imaging was performed with a Molecular Imager PharosFX Plus (Bio-Rad Laboratories).

1.9. Melting temperature (T_m) measurement (CD). A mixture (100 μL) of the photocatalyst-G4 DNA conjugate (1.0 μM) in 50 mM Tris-HCl buffer (pH 7.5) containing 100 mM KCl was annealed by incubation at 90 °C, followed by cooling to 4 °C at a rate of 0.5 °C/min. The mixture was transferred to a micro quartz cell with a 1 cm path length. The ellipticity was recorded at 290 nm from 25 °C to 90 °C at an interval of 1 °C, with temperature increase at a rate of 1 °C/min. The measurement was repeated three times and averaged to obtain the final value. The CD spectra were recorded on a J-720WI (JASCO Co., Hachioji, Japan) equipped with a Peltier temperature controller.

1.10. Melting temperature (T_m) measurement (UV-vis). A mixture (100 μL) of the photocatalyst-modified DNA duplex (1.0 μM) in 50 mM Tris-HCl buffer (pH 7.5) containing 100 mM KCl was transferred to a micro quartz cell with a 1-cm path length. The melting temperature was then measured under UV irradiation at 260 nm from 20 to 90 °C at the rate of 0.5 °C/min. The measurements were carried out three times per sample and averaged to obtain the final value. The melting temperature measurement was performed by a V-730 (JASCO Corporation) equipped with a temperature controller.

1.11. Fluorescence measurement for G4-cat. A mixture (50 μL) of the G4-BDP (0.5 μM) or G4-Ru (4.0 μM) in 50 mM Tris-HCl buffer (pH 7.5) containing 100 mM KCl was transferred to a quartz cell with a 3-mm path length. The emission spectra were obtained with an excitation wavelength of 470 nm for BDP or 455 nm for Ru complex at 25 °C. The fluorescence measurement was performed by an FP-6500 (JASCO Corporation) equipped with a temperature controller.

1.12. UP1 labeling with sodium azide. To validate the dominant contribution of the singlet oxygen ($^1O_2$) pathway in the MAUra-based proximity labeling, a quenching experiment was performed using sodium azide (NaN_3) as a selective $^1O_2$ quencher. The labeling reaction was conducted in a final volume of 30 µL in 50 mM Tris-HCl buffer (pH 7.5) containing 100 mM KCl. Purified UP1 (1.0 µM), calf thymus DNA (1 ng/µL), and MAUra-N_3 (final concentration 1 mM, from a 100 mM stock in DMSO) were mixed with each G4-conjugated photosensitizer probe (G4-Ru-loop, G4-Ru-term, G4-BDP-loop, or G4-BDP-term; final concentration 0.5 µM) and incubated on ice for 30 min. NaN_3 was added to designated samples at a final concentration of 10 mM and allowed to equilibrate for 30 min on ice before irradiation. The reaction mixture was then photo-irradiated on ice with a 455 nm LED light (DC8-12V 15M Max, 200 mA MAX) for 30 sec (G4-Ru-loop and G4-Ru-term) or 10 min (G4-BDP-loop and G4-BDP-term). For Cys alkylation, 2-iodoacetamide (final concentration 10 mM) was added to the reaction mixture and incubated for 1 h at room temperature in the dark. To visualize labeled proteins, DBCO-Cy3 (final concentration 100 µM, from a 1.0 mM stock in DMSO) was added and incubated for 1 h at 37 °C. The labeled proteins were separated by SDS-PAGE (12% gel), and fluorescence imaging was performed using a Molecular Imager PharosFX Plus (Bio-Rad Laboratories, Inc.). The gel was subsequently stained with Coomassie Brilliant Blue (CBB) to confirm equal protein loading.

1.13. CD spectroscopy before and after photo-irradiation

To examine whether photo-irradiation under the labeling conditions induces structural changes in the G4 conformation, CD measurements were performed before and after irradiation. Solutions of G4-Ru-loop, G4-Ru-term, G4-BDP-loop, and G4-BDP-term (1.0 µM each) in 50 mM Tris-HCl buffer (pH 7.5) containing 100 mM KCl were annealed by incubation at 90 °C, followed by slow cooling to 4 °C at a rate of 0.5 °C/min. CD spectra were first recorded at 20 °C using a J-820 spectropolarimeter (JASCO Corporation) equipped with a Peltier temperature controller (1-cm path length quartz cell, wavelength range 220–350 nm). MAUra (final concentration 100 µM, from a 100 mM stock in DMSO) was then added to each sample, and the solutions were transferred to microtubes and irradiated on ice with a 455 nm LED light (DC8-12V 15M Max, 200 mA MAX) for 30 sec (G4-Ru-loop and G4-Ru-term) or 10 min (G4-BDP-loop and G4-BDP-term), replicating the conditions used in the protein-labeling experiments. After irradiation, MAUra was removed by ultrafiltration using an Amicon Ultra 0.5 mL 3K filter (Millipore), and the buffer was exchanged to 50 mM Tris-HCl (pH 7.5) containing 100 mM KCl. The samples were then transferred back to the quartz cell, and CD

spectra were re-recorded under identical conditions. For each spectrum, the spectrum of the corresponding buffer was subtracted; no further processing was applied. Spectra before and after irradiation were overlaid for comparison.

1.14. EMSA-based assessment of protein binding after photo-irradiation

To evaluate whether photo-irradiation under the labeling conditions affects the G4-binding ability of UP1, EMSA was performed with irradiated G4 constructs. G4-Ru-loop, G4-Ru-term, G4-BDP-loop, and G4-BDP-term (50 nM each, in 50 mM Tris-HCl, pH 7.5, 100 mM KCl) were annealed as described above. Each sample was photo-irradiated on ice with a 455 nm LED light for 30 sec (Ru constructs) or 10 min (BDP constructs). Non-irradiated samples were kept on ice throughout as controls. EMSA binding reactions were conducted in a 20 µL volume [50 mM Tris-HCl (pH 7.5) containing 100 mM KCl, UP1 (0.5 µM), and irradiated or non-irradiated G4 (50 nM)] for 30 min on ice. Complexes were resolved by 6% non-denaturing PAGE (19:1 acrylamide:bis-acrylamide) in 0.5× TBE at 4 °C, 200 V, for 1 h. The gel was stained with SYBR Gold (Thermo Fisher Scientific), and fluorescence imaging was performed with a Molecular Imager PharosFX Plus (Bio-Rad Laboratories).

1.15. Protein-Protein Interaction (PPI) Network Analysis. To predict the functional interaction networks among the identified G4-interacting proteins, the enriched protein dataset obtained from the comparison of G4-Ru-term versus free Ru was analyzed using the STRING database (Search Tool for the Retrieval of Interacting Genes/Proteins, version 12.0, https://string-db.org/). A list of 168 proteins that met the stringent threshold (-log10(*p* value) > 4 and log2(fold change) > 2.5) was subjected to the Multiple proteins search module. The network edges were defined by both physical and functional associations based on the default settings with a minimum required interaction score of 0.700 (High confidence). To identify highly connected subnetworks and functional modules, the Markov Cluster Algorithm (MCL) was applied to the generated network with an inflation parameter of 3. Functional enrichment analysis, including Gene Ontology (GO) biological processes and their corresponding false discovery rates (FDR), was simultaneously retrieved from the STRING analysis platform to statistically validate the specificity of the captured protein complexes.

1.16. Microscale thermophoresis (MST) measurement. The binding affinity of HK1 toward HT G4 DNA was quantified by microscale thermophoresis (MST) using a Monolith NT.115 instrument (NanoTemper Technologies, Munich, Germany). HT G4 DNA labeled with Texas Red at its 3' end (HT-Texas Red) was annealed and diluted to a final concentration of 20 nM in MST assay buffer (10 mM Tris-HCl, pH 7.5, 20 mM KCl, 0.05% Tween-20). A dilution

series of purified HK1 protein (ranging from 0.1 nM to 3.2 µM, 16 two-fold dilutions) was prepared in the same buffer. Each HK1 dilution was mixed with the HT-Texas Red G4 solution and incubated at 25 °C for 30 min. The samples were loaded into standard capillaries (Monolith Premium Capillaries, NanoTemper Technologies) and measurements were performed at 25 °C using 20% LED excitation power and medium MST power. The K_d values are reported as mean ± standard deviation from three independent experiments. The thermophoresis data were analyzed using MO. Affinity Analysis software (NanoTemper Technologies). The normalized fluorescence change (Fnorm) was plotted against HK1 concentration, and the equilibrium dissociation constant (K_d) was determined by fitting data to a standard 1:1 binding model using the equation:

$$\Delta Fnorm = \Delta Fnorm,max \times [HK1] / (K_d + [HK1])$$

Data points at HK1 concentrations ≥400 nM (the four highest concentrations tested) were excluded from fitting owing to fluorescence quenching of Texas Red by HK1 at high protein concentrations, as evidenced by a consistent decrease in both the capillary fluorescence intensity and Fnorm values.

2. Additional data and figures:

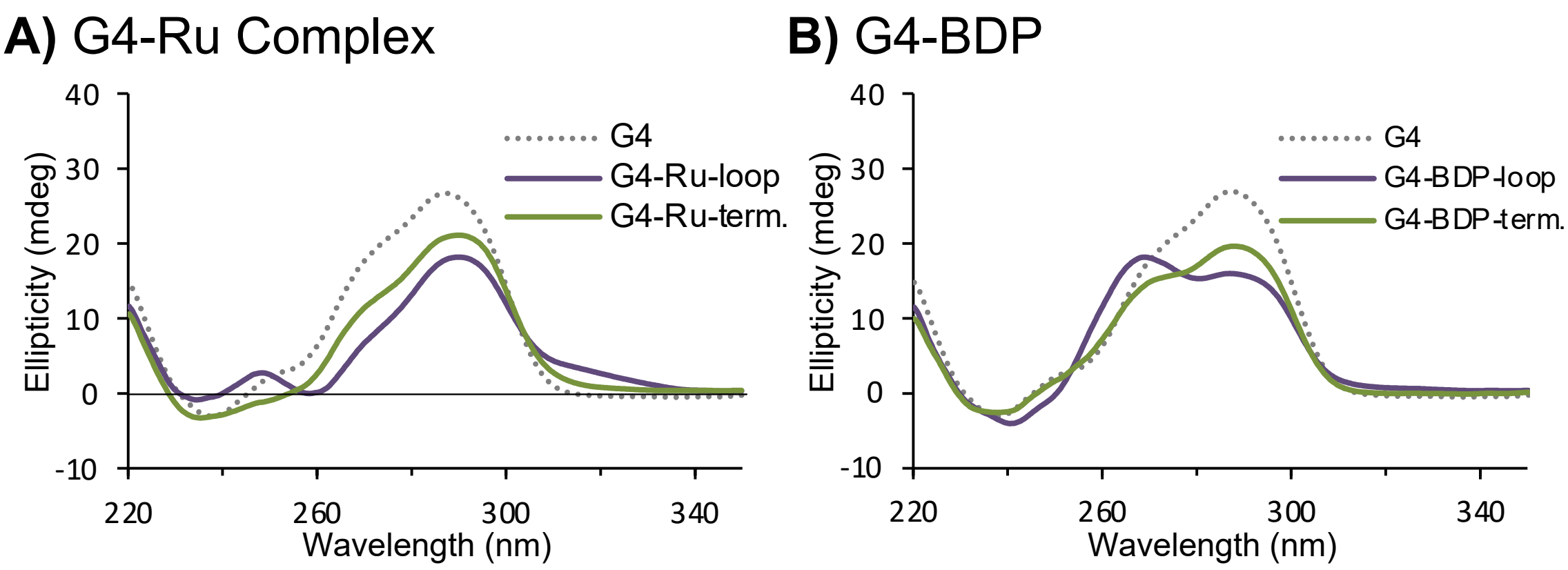

Figure S1. The circular dichroism (CD) spectra measurement for native G4 and G4-cat. The CD spectra were measured for unmodified HT DNA-G4 sequence (dotted gray line) and photocatalyst-modified HT DNA-G4 sequences at the internal loop (purple line) and at the 5′ terminal (green line) positions for **A**) Ru complex and **B**) BDP. CD measurement conditions: The pre-annealed DNA or DNA-cat (5.0 µM), 50 mM Tris-HCl buffer (pH 7.5), 100 mM KCl at 20 °C.

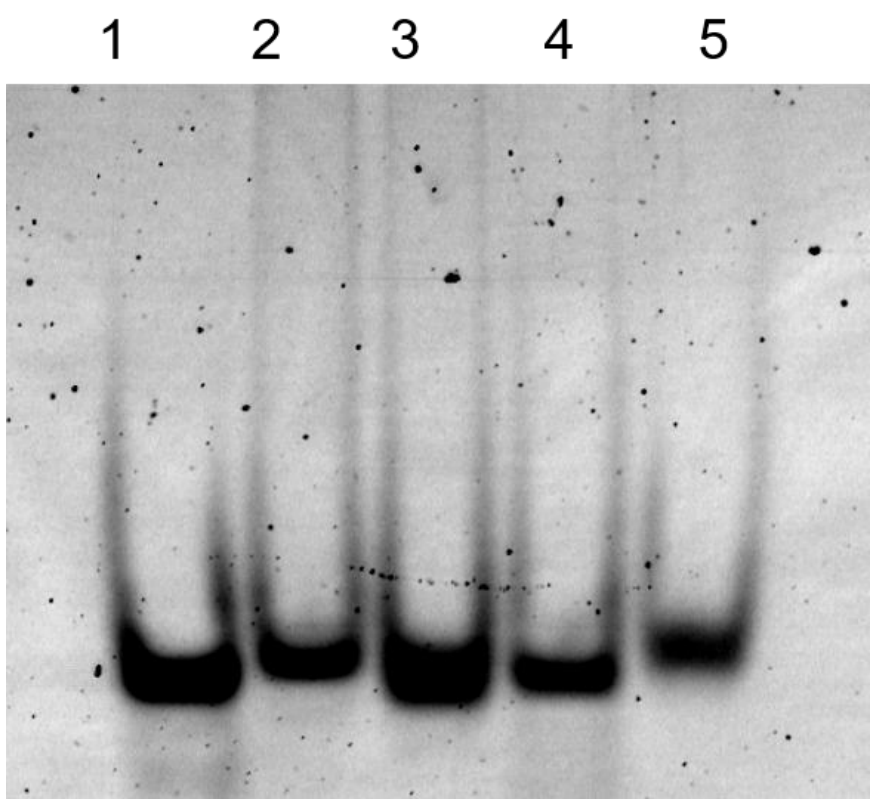

Figure S2. Native PAGE analysis of G4 topology for photosensitizer-modified G4 sequences. Native polyacrylamide gel electrophoresis (12%) was performed to assess the G4-folded structure of photosensitizer-modified sequences. Each G4 sample (0.1 µM in 50 mM Tris-HCl, pH 7.5, 100 mM KCl) was annealed by heating at 90 °C followed by slow cooling to 4 °C (0.5 °C/min). Lane 1, unmodified G4 (HT G4); Lane 2, G4-BDP-term; Lane 3, G4-BDP-loop; Lane 4, G4-Ru-term; Lane 5, G4-Ru-loop. Electrophoresis was carried out in 0.5× TBE supplemented with 50 mM KCl at 4 °C, 120 V, for 60 min. DNA was visualized by SYBR Gold staining.

Oligomer	Sequence (5' to 3')	T_m (°C)	ΔT_m (°C)
Native G4	TA**GGG**TTA**GGG**TTA**GGG**TTA**GGG**	71	--
G4-Ru-loop	TA**GGG**TTA**GGG**T^{Ru}TA**GGG**TTA**GGG**	74	+3
G4-Ru-term	Ru-TA**GGG**TTA**GGG**TTA**GGG**TTA**GGG**	80	+9
G4-BDP-loop	TA**GGG**TTA**GGG**T^{BDP}TA**GGG**TTA**GGG**	69	-2
G4-BDP-term	BDP-TA**GGG**TTA**GGG**TTA**GGG**TTA**GGG**	71	0

Table S1. The T_m values for native G4 and G4-cat. T_m measurements conditions were as follows: 1.0 µM DNA, 50 mM Tris-HCl buffer (pH 7.5), 100 mM KCl. The ellipticity was recorded at 290 nm from 25 ℃ to 90 ℃ at an interval of 1 ℃, with temperature increase at a rate of 1 ℃/min.

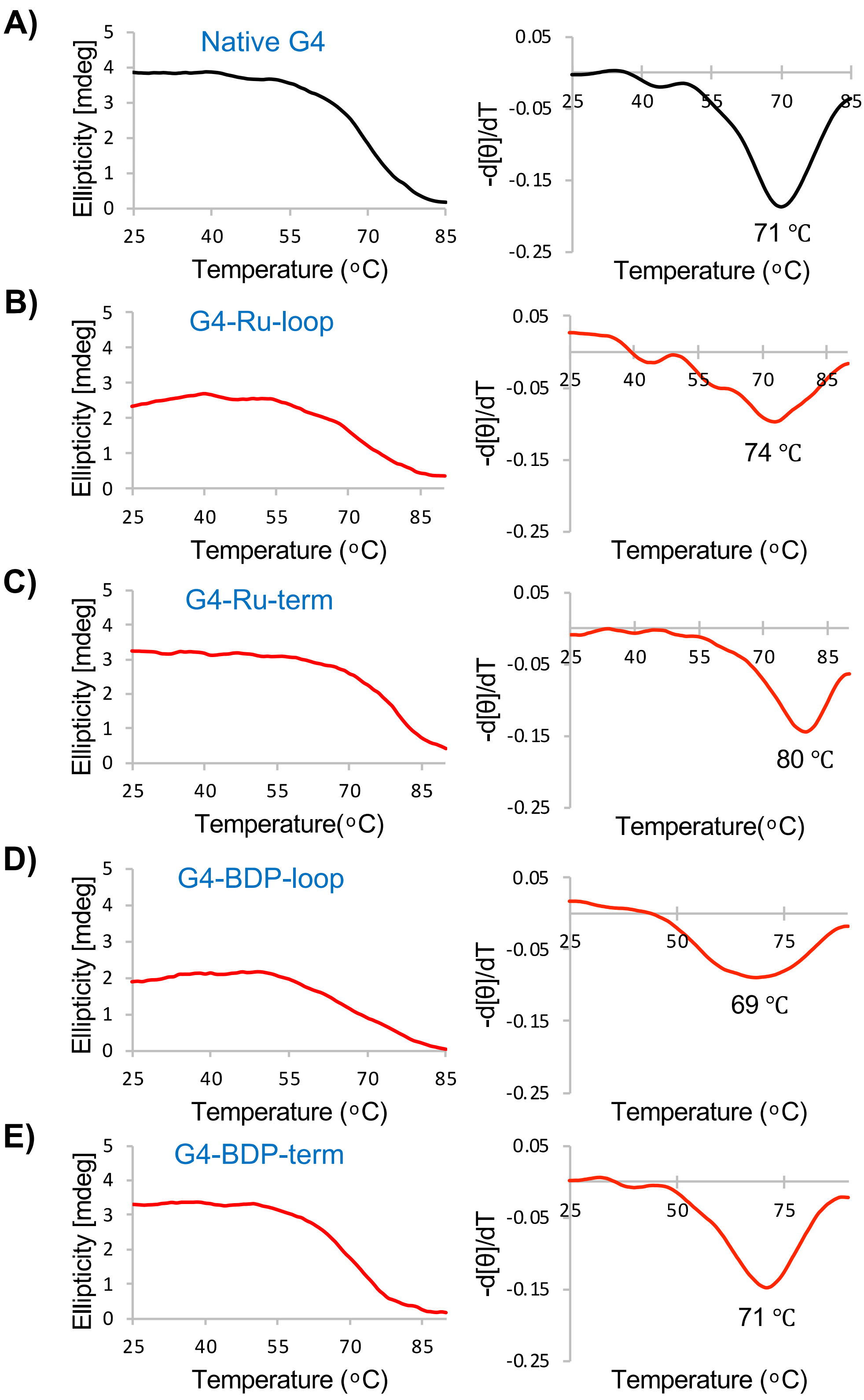

Figure S3. The thermal stability (T_m) of HT DNA G4-cat using CD melting measurements. T_m curves were recorded for (A) native G4, (B,C) Ru-complex-modified G4, and (D,E) BDP-modified G4.

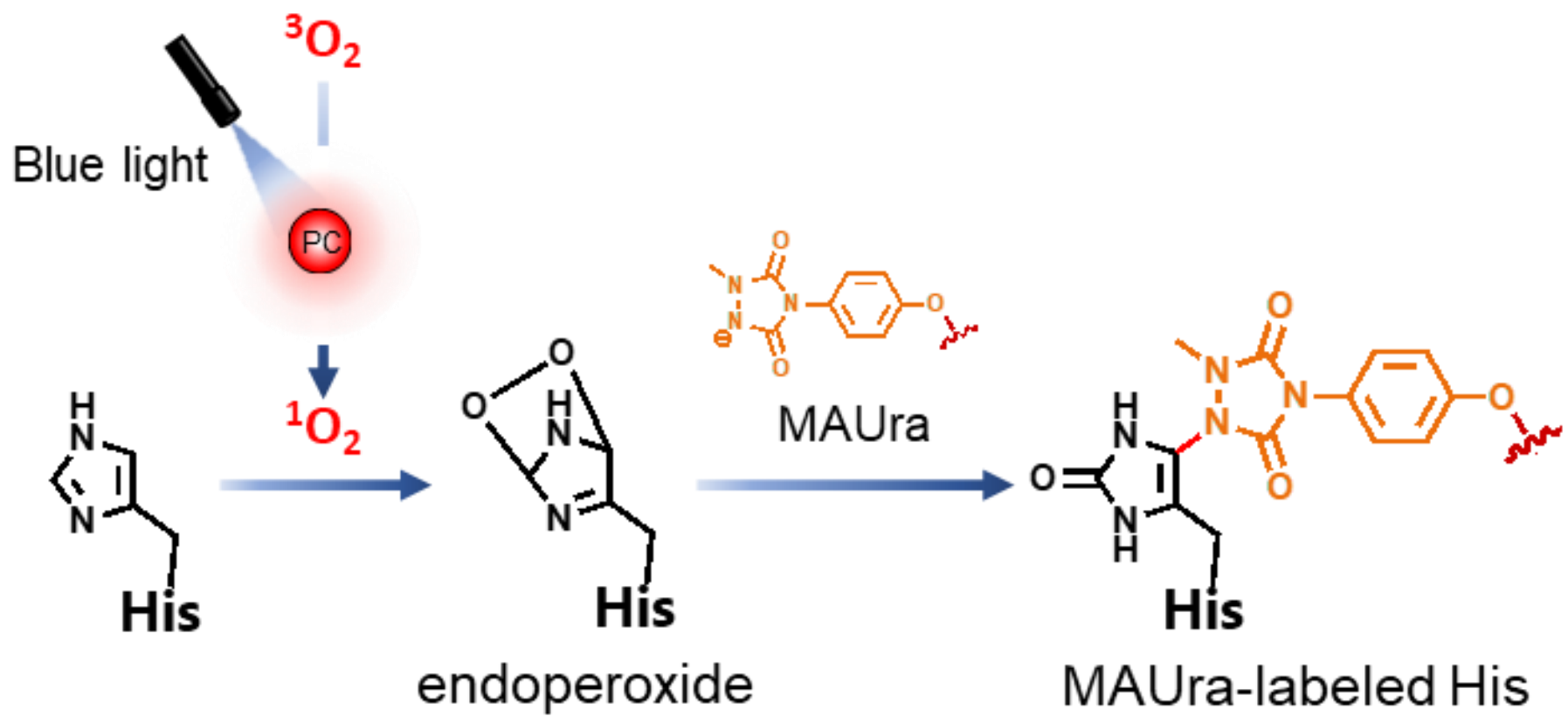

Figure S4. The Mechanism of photocatalytic histidine labeling using BODIPY or Ru complex.

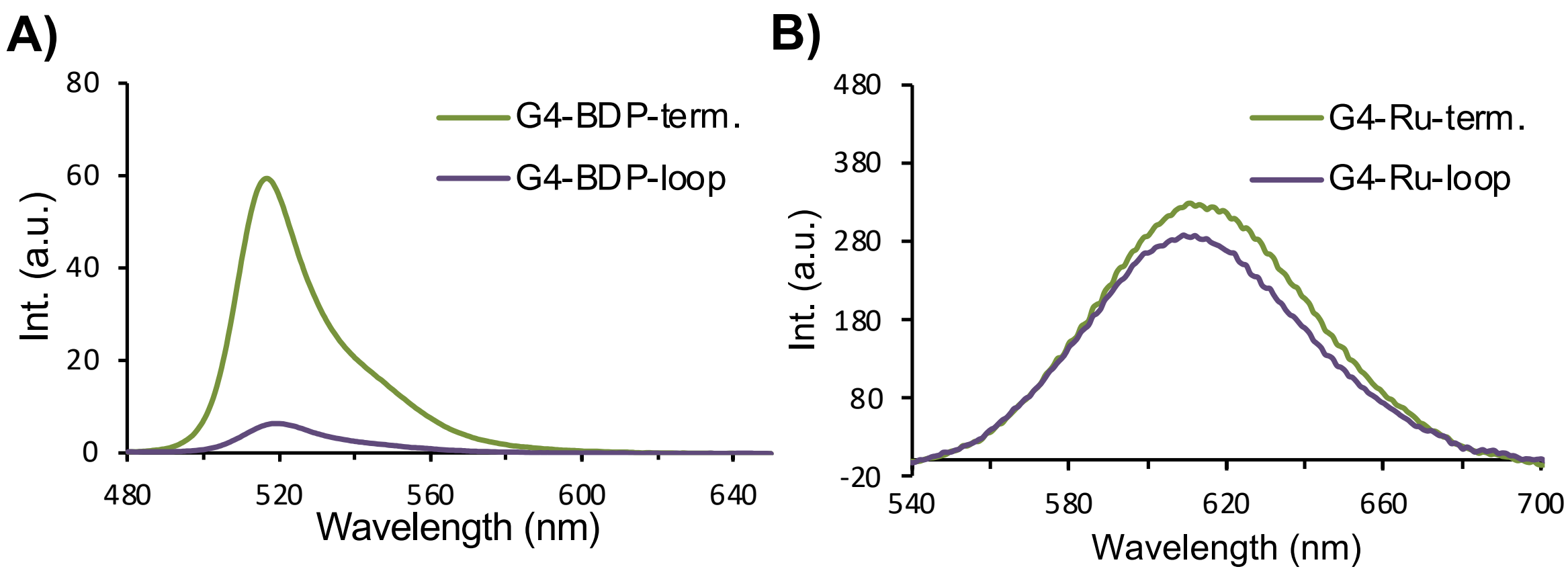

Figure S5. The fluorescence spectra measurement for A) G4-BDP and B) G4-Ru. Measurement conditions: G4-BDP [0.5 µM] or G4-Ru [4.0 µM] in Tris-HCl buffer (pH 7.5) [50 mM] containing KCl [100 mM]. Excitation wavelength: 470 nm (for BDP) or 455 nm (for Ru) at 25 ℃.

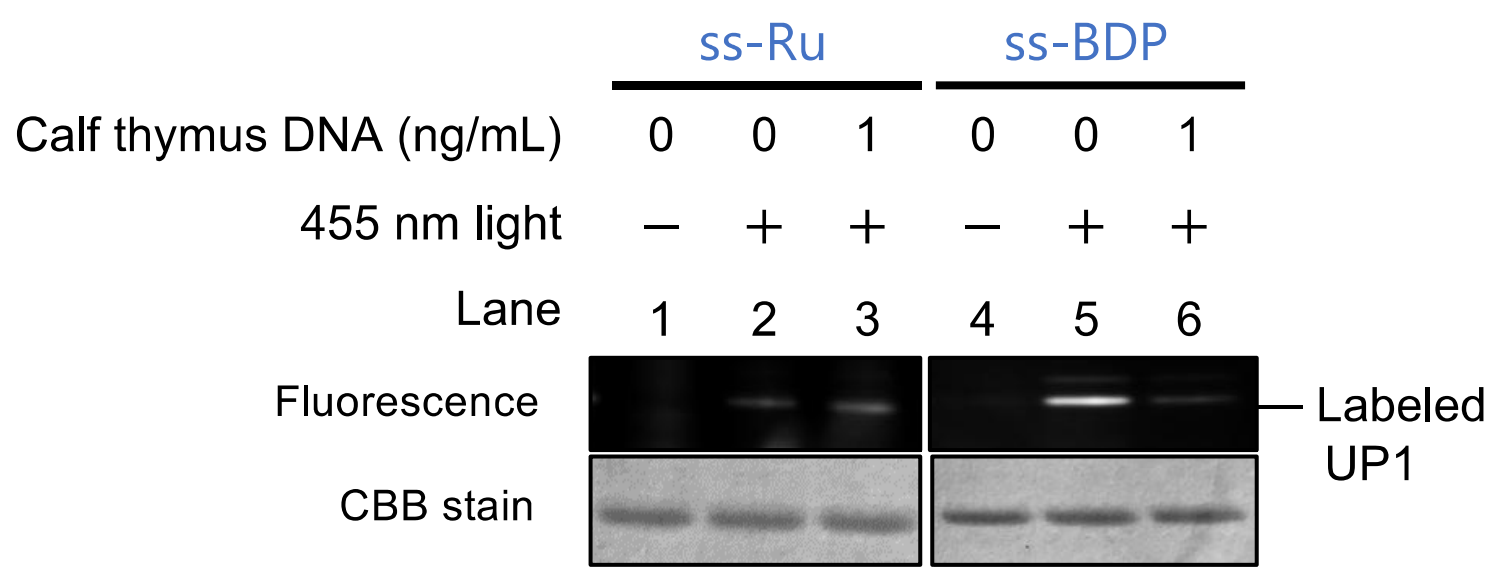

Figure S6. The photolabeling reaction of unwinding (UP1) protein using ss-Ru (left) and ss-BDP (right) with MAUra-azide labeling reagent in the presence and absence of calf thymus DNA. Reaction conditions were as follows: ss-cat [0.5 µM], MAUra-N_3 [1.0 mM], UP1 [1.0 µM], calf thymus DNA [1 ng/µL], in 50 mM Tris-HCl buffer (pH 7.5) containing KCl [100 mM]. 455 nm LED light, 0 ℃ for 30 sec (Ru complex) or 10 min (BDP).

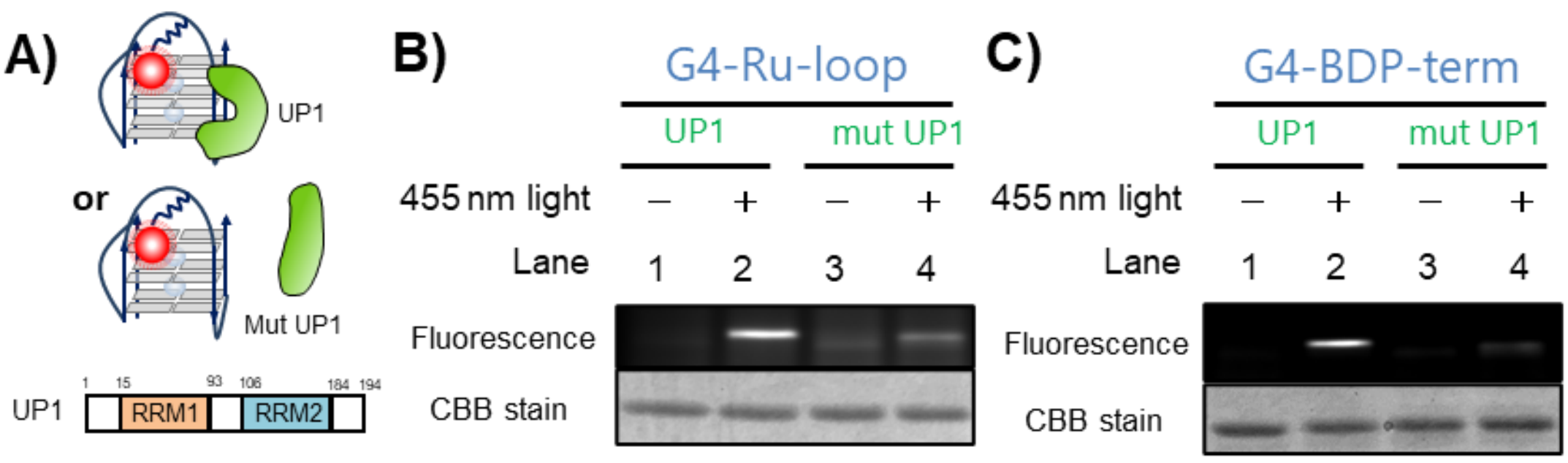

Figure S7. The photolabeling reaction for UP1 and mutated UP1 (in which R55 (in RRM1) and R146 (in RRM2) amino acids of UP1 were replaced with A55 and A146) proteins with MAUra-azide labeling reagent. A) Schematic represents the binding of UP1 and lack of binding of mutated UP1. **B**) SDS-PAGE analysis of G4-Ru-loop-mediated labeling reaction. **C**) SDS-PAGE analysis of G4-BDP-term-mediated photolabeling reaction. Reaction conditions were as follows: G4-cat [0.5 µM], MAUra-N_3 [1.0 mM], UP1 / mut UP1 [1.0 µM], calf thymus DNA [1 ng/µL], in 50 mM Tris-HCl buffer (pH 7.5) containing KCl [100 mM]. 455 nm LED light, 0 ℃ for 30 sec (Ru complex) or 10 min (BDP).

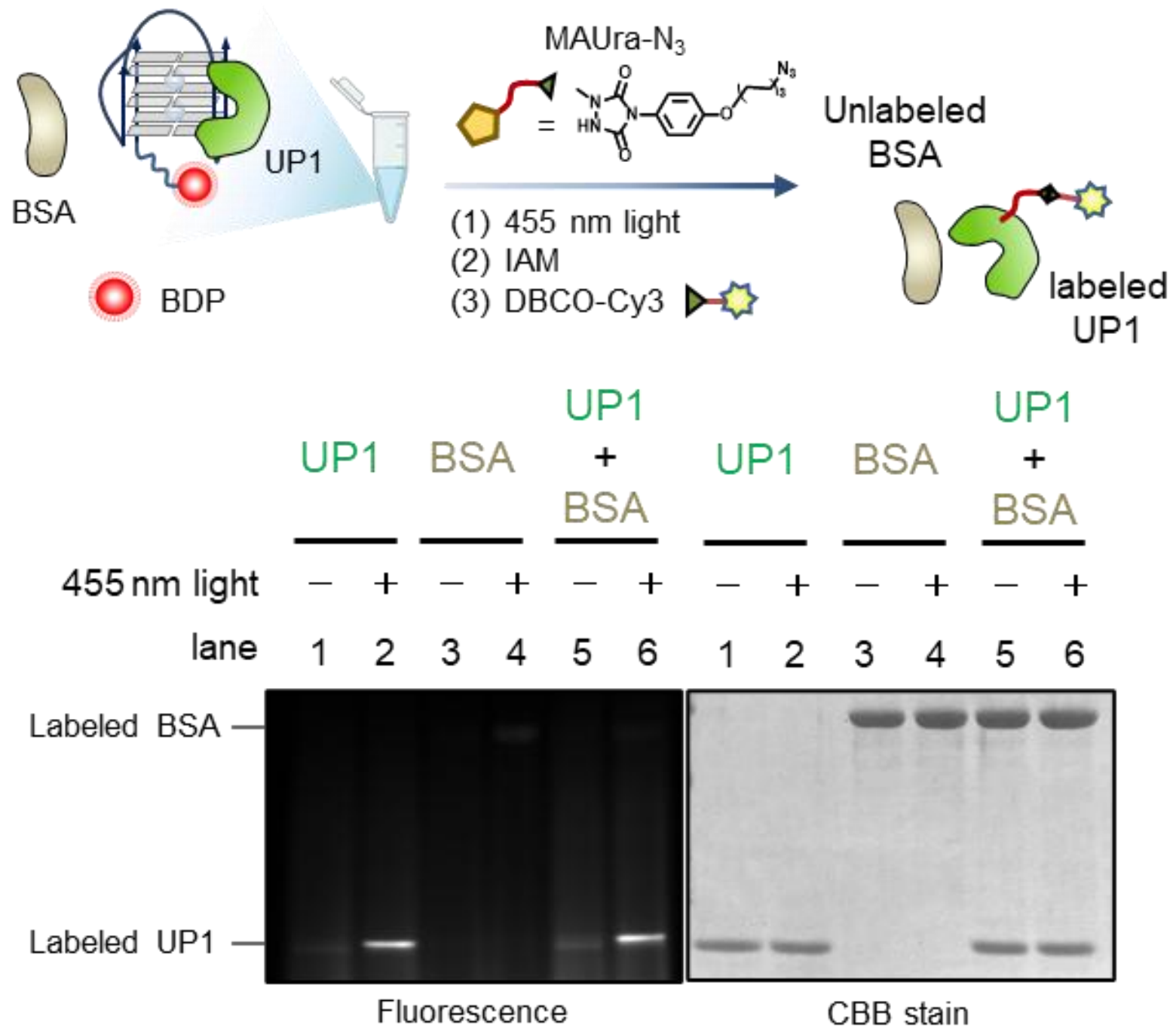

Figure S8. The UP1 labeling in the presence of BSA using G4-BDP-term. G4-BDP [0.5 µM], MAUra-N_3 [1.0 mM], UP1 [1.0 µM], BSA [1.0 µM], calf thymus DNA [1 ng/µL] in 50 mM Tris-HCl buffer (pH 7.5) containing KCl [100 mM]. The reaction mixture was photoirradiated with a 455 nm LED Light for 10 min at 0 ℃. The labeled protein was visualized by DBCO-Cy3.

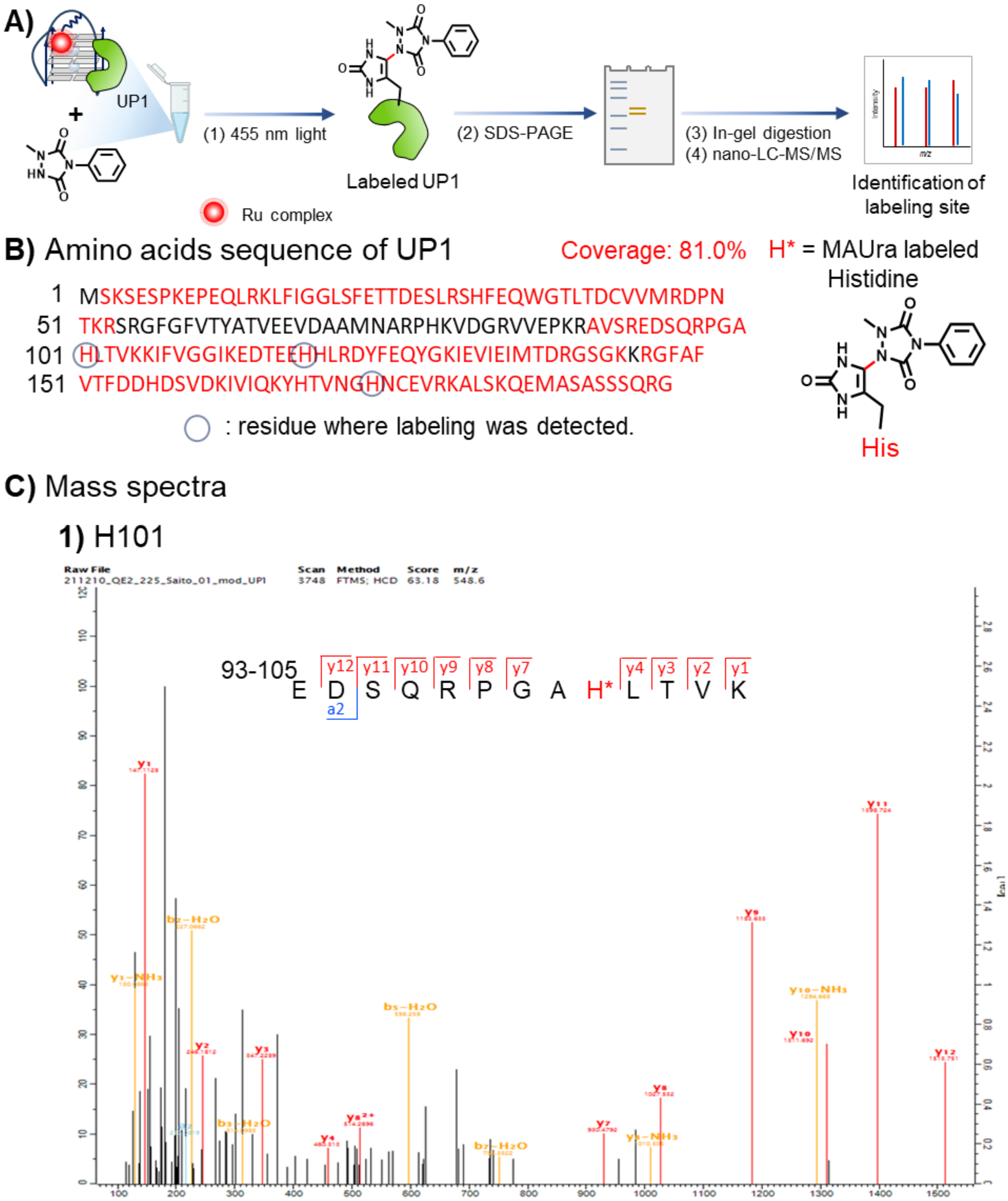

Figure S9. NanoLC-MS/MS analysis of trypsin-digested peptide fragments of non-azide MAUra-labeled UP1 for G4-Ru-loop. A) Schematic workflow of identification of UP1 labeling site. **B**) Amino acids sequence of UP1. **C**) MS/MS fragment assignment of labeled peptide in H101, H119, and H173. Reaction conditions: G4-Ru-loop [0.5 μM], non-azide MAUra [1.0 mM], UP1 [1.0 μM], calf thymus DNA [1 ng/μL], in 50 mM Tris-HCl buffer (pH 7.5) containing KCl [100 mM]. 455 nm LED light, 30 sec, 0 ℃.

2) H119

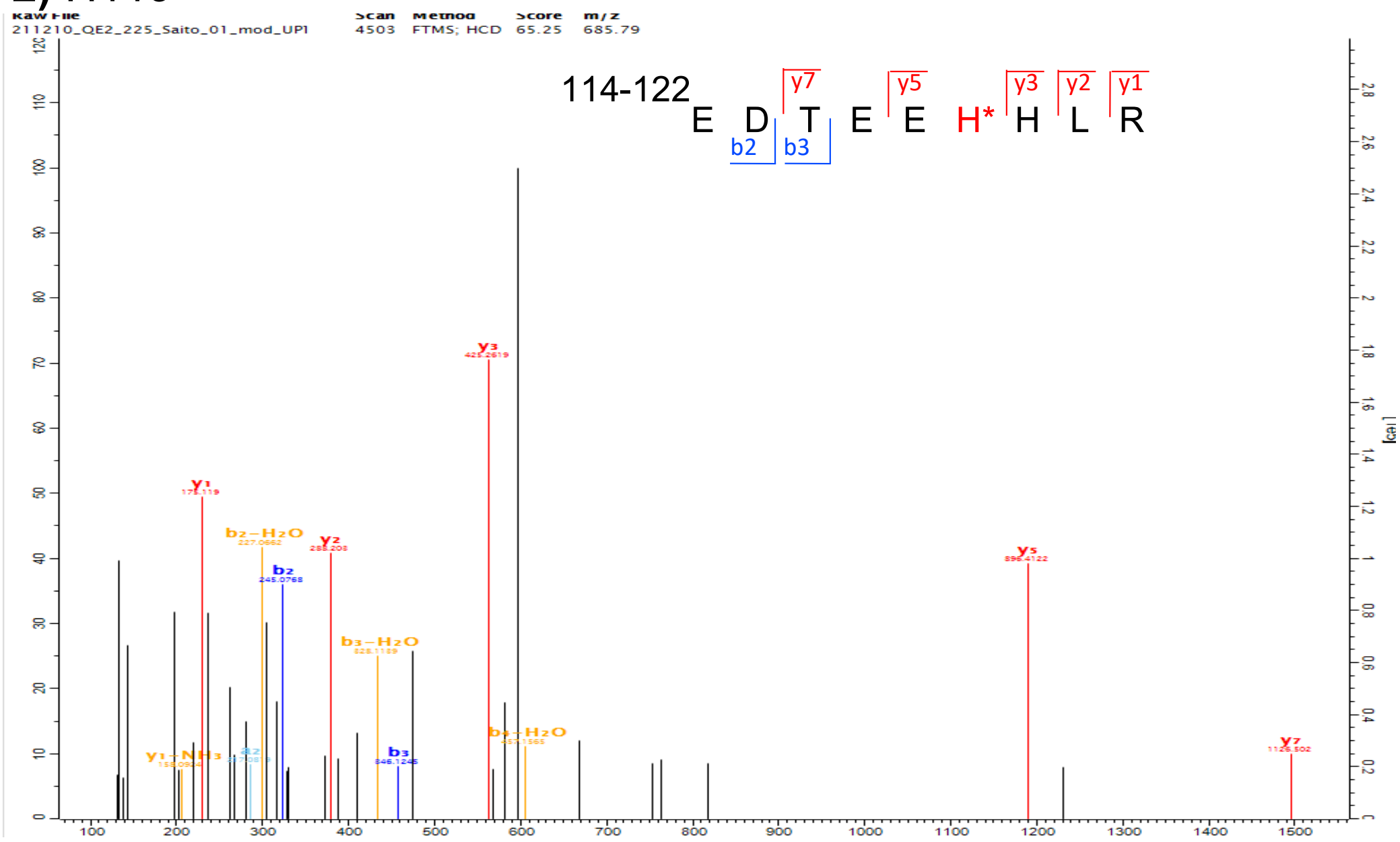

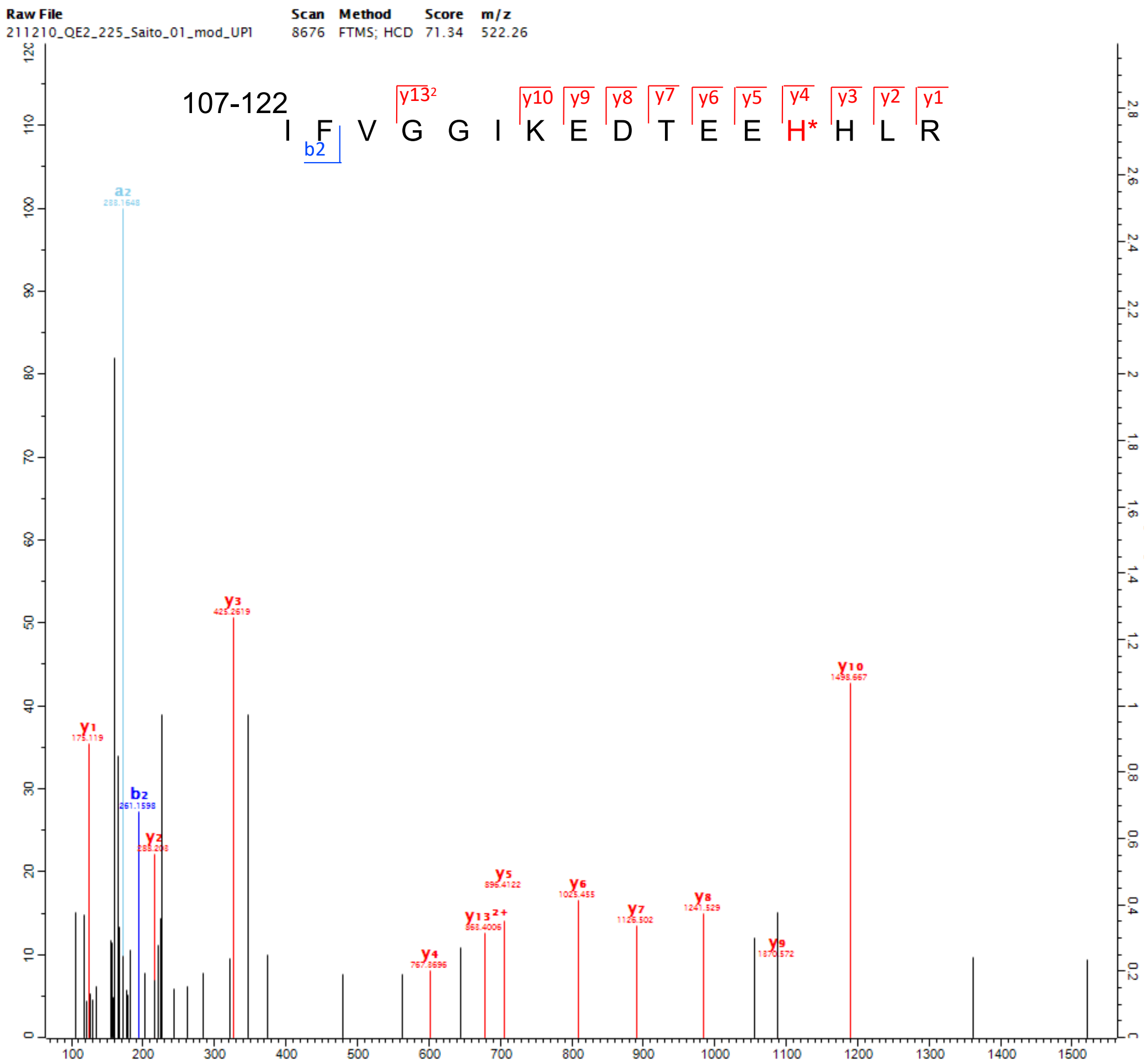

Figure S9. (continued)

3) H173

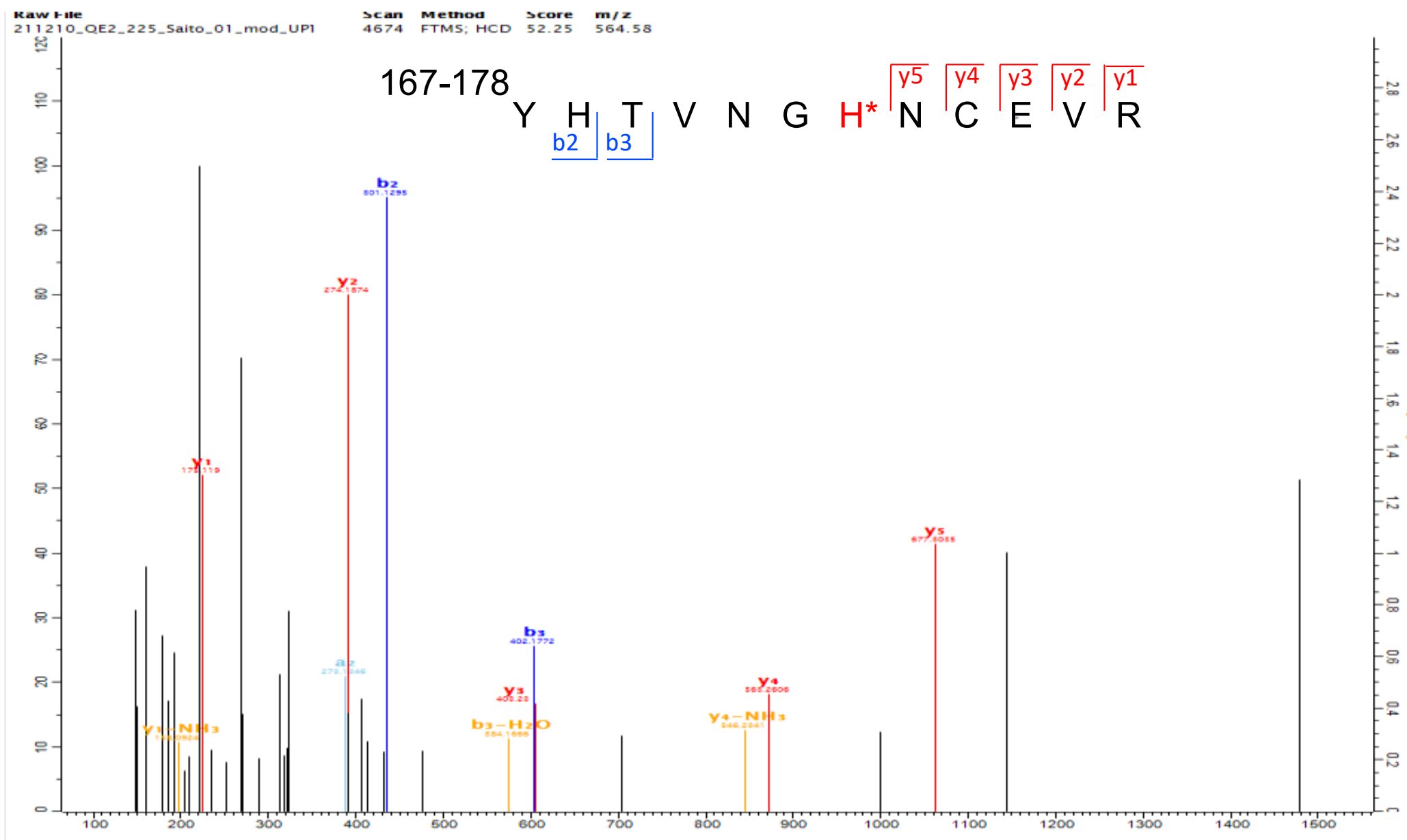

Figure S9. (continued)

	Annotated Sequence	Modifications
1	YHTVNGHNCEVR	3 Oxidation(+O) (HWY)
2	IVIQKYHTVNGHNCEVR	Carbamidomethyl (C)
3	YHTVNGHNCEVR	Carbamidomethyl (C)
4	YHTVNGHNCEVRK	Carbamidomethyl (C)
5	SHFEQWGTLTDCVVMR	Carbamidomethyl (C);Oxidation(+O) (HWY)
6	EDSQRPGAHLTVK	Dioxidation (H)
7	EDSQRPGAHLTVKK	Dioxidation (H)
8	EDTEEHHLR	Dioxidation (H)
9	IFVGGIKEDTEEHHLR	Dioxidation (H)
10	KIFVGGIKEDTEEHHLR	Dioxidation (H)
11	YHTVNGHNCEVR	Dioxidation (H)
12	EDSQRPGAHLTVK	MAUra_His
13	EDTEEHHLR	MAUra_His
14	IFVGGIKEDTEEHHLR	MAUra_His
15	YHTVNGHNCEVR	MAUra_His
16	IEVIEIMTDR	Oxidation (M)
17	QEMASASSSQR	Oxidation (M)
18	SHFEQWGTLTDCVVMR	Oxidation (M)
19	SHFEQWGTLTDCVVMRDPNTK	Oxidation (M)
20	SHFEQWGTLTDCVVMRDPNTKR	Oxidation (M)
21	SHFEQWGTLTDCVVMR	Oxidation (M);Dioxidation (MW)
22	DYFEQYGK	Oxidation(+O) (HWY)
23	IFVGGIKEDTEEHHLR	Oxidation(+O) (HWY)
24	IVIQKYHTVNGHNCEVR	Oxidation(+O) (HWY)
25	ALSKQEMASASSSQR	Unmodified
26	ALSKQEMASASSSQRG	Unmodified
27	AVSREDSQRPGAHLTVK	Unmodified
28	DYFEQYGK	Unmodified
29	EDSQRPGAHLTVK	Unmodified
30	EDSQRPGAHLTVKK	Unmodified
31	EDTEEHHLR	Unmodified
32	EDTEEHHLRDYFEQYGK	Unmodified
33	GFAFVTFDDHDSVDK	Unmodified
34	IEVIEIMTDR	Unmodified
35	IEVIEIMTDRGSGK	Unmodified
36	IFVGGIK	Unmodified
37	IFVGGIKEDTEEHHLR	Unmodified
38	IVIQKYHTVNGHNCEVR	Unmodified
39	KALSKQEMASASSSQR	Unmodified
40	KIFVGGIK	Unmodified
41	KIFVGGIKEDTEEHHLR	Unmodified
42	KLFIGGLSFETTDESLR	Unmodified
43	LFIGGLSFETTDESLR	Unmodified
44	PGAHLTVK	Unmodified
45	QEMASASSSQR	Unmodified
46	RGFAFVTFDDHDSVDK	Unmodified
47	SESPKEPEQLR	Unmodified
48	SESPKEPEQLRK	Unmodified
49	SHFEQWGTLTDCVVMR	Unmodified
50	SHFEQWGTLTDCVVMRDPNTK	Unmodified
51	SKSESPKEPEQLR	Unmodified
52	YHTVNGHNCEVR	Unmodified
53	YHTVNGHNCEVRK	Unmodified

Table S2. List of detected UP1 fragments by nanoLC-MS/MS analysis for G4-Ru-loop.

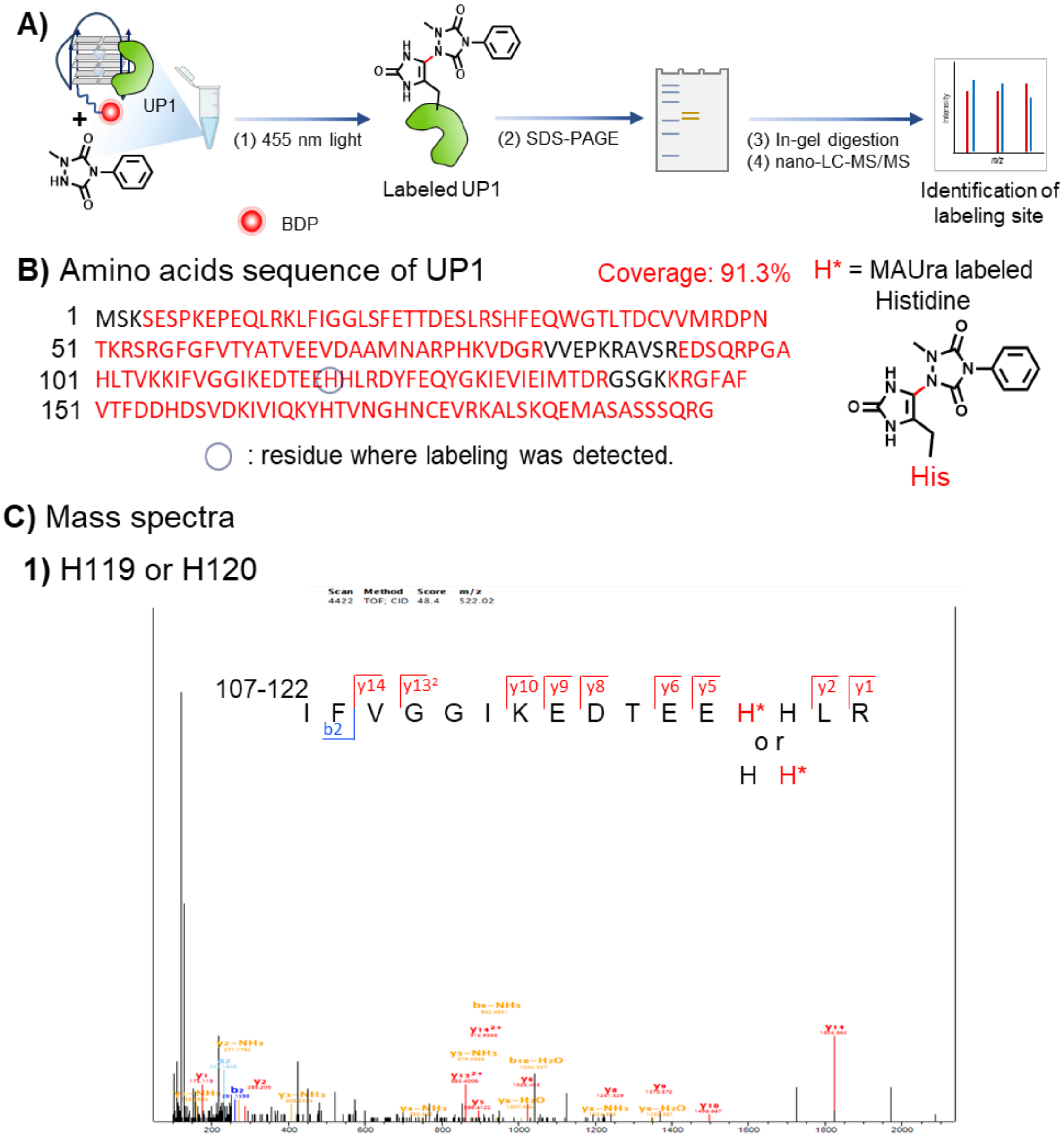

Figure S10. NanoLC-MS/MS analysis of trypsin-digested peptide fragments of non-azide MAUra-labeled UP1 for G4-BDP-term. A) Schematic workflow of identification of UP1 labeling site. **B**) Amino acids sequence of UP1. **C**) MS/MS fragment assignment of labeled peptide in H119 or H120. Reaction conditions: G4-BDP-terminal [0.5 µM], non-azide MAUra [1.0 mM], UP1 [1.0 µM], calf thymus DNA [1 ng/µL], in 50 mM Tris-HCl buffer (pH 7.5) containing KCl [100 mM]. 455 nm LED light, 10 min, 0 ℃.

	Annotated Sequence	Modifications
1	IFVGGIKEDTEEHHLR	2 Oxidation(+O) (HWY)
2	IVIQKYHTVNGHNCEVR	Carbamidomethyl (C)
3	SHFEQWGTLTDCVVMR	Carbamidomethyl (C)
4	SHFEQWGTLTDCVVMRDPNTK	Carbamidomethyl (C)
5	SHFEQWGTLTDCVVMRDPNTKR	Carbamidomethyl (C)
6	IFVGGIKEDTEEHHLR	MAUra_His
7	GFGFVTYATVEEVDAAMNARPHK	Oxidation (M)
8	IEVIEIMTDR	Oxidation (M)
9	SHFEQWGTLTDCVVMRDPNTKR	Oxidation (M)
10	GFGFVTYATVEEVDAAMNARPHK	Oxidation(+O) (HWY)
11	IVIQKYHTVNGHNCEVR	Oxidation(+O) (HWY)
12	SHFEQWGTLTDCVVMR	Oxidation(+O) (HWY)
13	SHFEQWGTLTDCVVMRDPNTK	Oxidation(+O) (HWY)
14	SHFEQWGTLTDCVVMRDPNTKR	Oxidation(+O) (HWY)
15	ALSKQEMASASSSQRG	Unmodified
16	DYFEQYGK	Unmodified
17	DYFEQYGKIEVIEIMTDR	Unmodified
18	EDSQRPGAHLTVK	Unmodified
19	EDSQRPGAHLTVKK	Unmodified
20	EDTEEHHLRDYFEQYGK	Unmodified
21	GFAFVTFDDHDSVDK	Unmodified
22	GFAFVTFDDHDSVDKIVIQK	Unmodified
23	GFGFVTYATVEEVDAAMNARPHK	Unmodified
24	GFGFVTYATVEEVDAAMNARPHKVDGR	Unmodified
25	IEVIEIMTDR	Unmodified
26	IFVGGIKEDTEEHHLR	Unmodified
27	IFVGGIKEDTEEHHLRDYFEQYGK	Unmodified
28	IVIQKYHTVNGHNCEVR	Unmodified
29	KIFVGGIKEDTEEHHLR	Unmodified
30	KLFIGGLSFETTDESLR	Unmodified
31	KRGFAFVTFDDHDSVDK	Unmodified
32	LFIGGLSFETTDESLR	Unmodified
33	RGFAFVTFDDHDSVDK	Unmodified
34	RGFAFVTFDDHDSVDKIVIQK	Unmodified
35	SESPKEPEQLR	Unmodified
36	SESPKEPEQLRK	Unmodified
37	SHFEQWGTLTDCVVMR	Unmodified
38	SHFEQWGTLTDCVVMRDPNTK	Unmodified
39	SHFEQWGTLTDCVVMRDPNTKR	Unmodified
40	SRGFGFVTYATVEEVDAAMNARPHK	Unmodified
41	YHTVNGHNCEVR	Unmodified
42	YHTVNGHNCEVRK	Unmodified

Table S3. List of detected UP1 fragments by nanoLC-MS/MS analysis for G4-BDP-terminal.

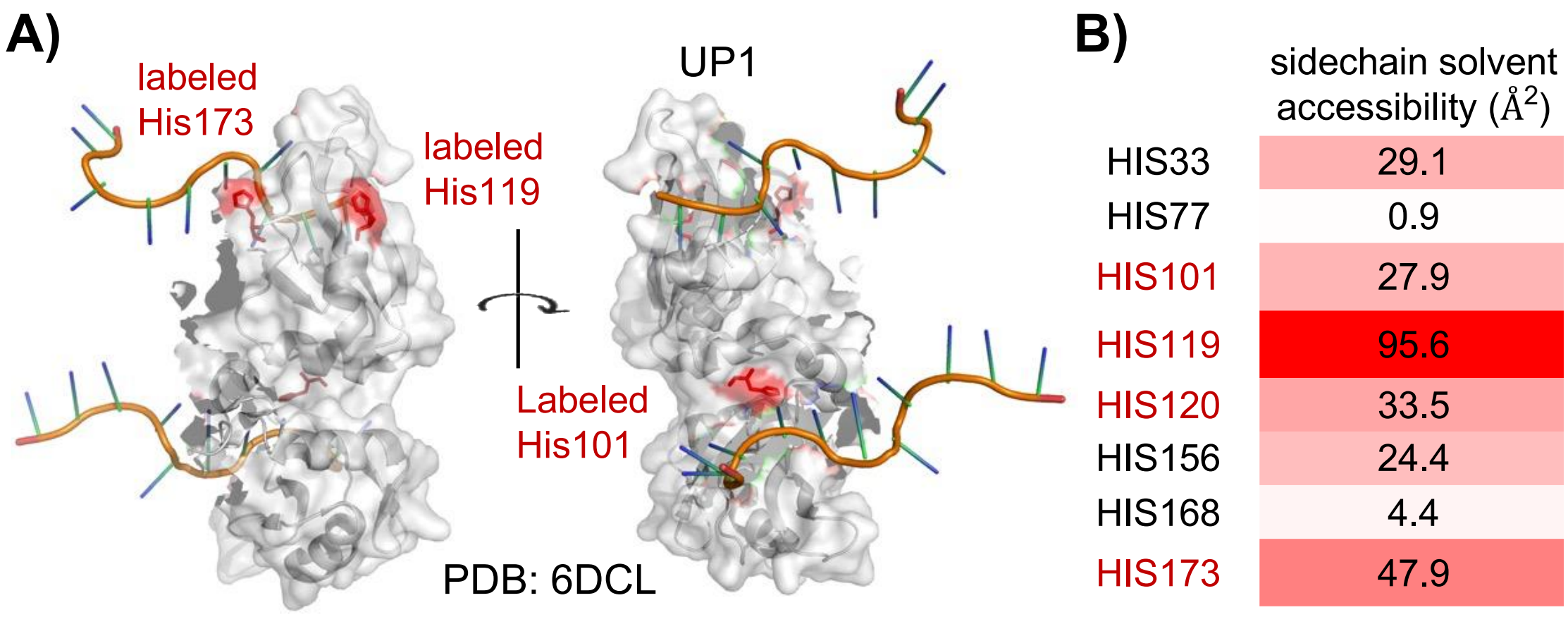

Figure S11. Histidine labeling. A) X-ray crystal structure of UP1 with HT RNA. The MAUra-labeled histidine residues are marked in red. **B**) Side chain solvent accessibilities (Å2) were estimated by discovery studio 4.1 and PDB: 6DCL.

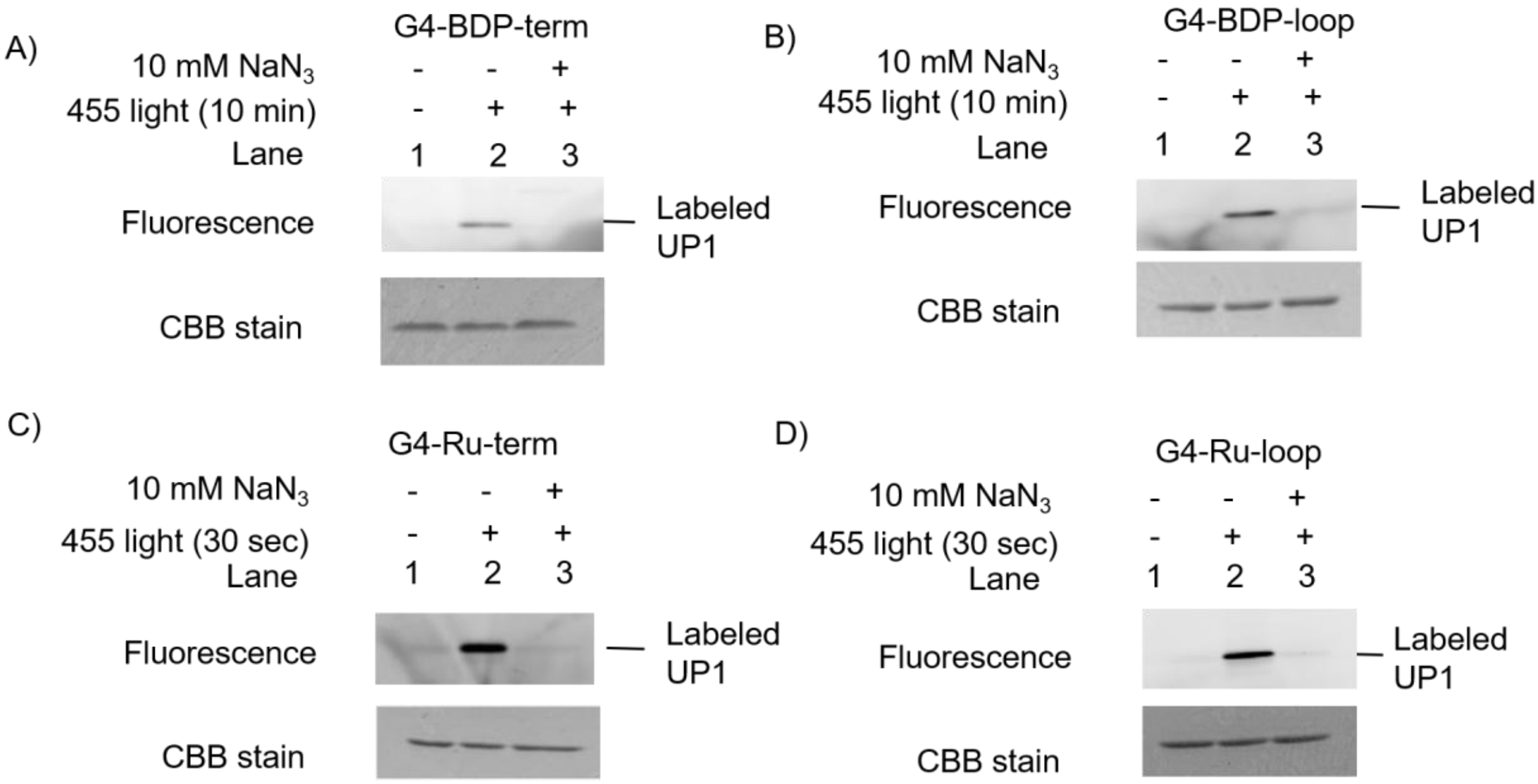

Figure S12. Singlet oxygen quenching assay with sodium azide validates the $^1O_2$-dependent mechanism of MAUra-based proximity labeling. UP1 (1.0 µM) was incubated with each G4-conjugated photosensitizer probe A) G4-BDP-term, B) G4-BDP-loop, C) G4-Ru- term, or D) G4-Ru-loop (0.5 µM) and MAUra-N_3 (1 mM) in 50 mM Tris-HCl (pH 7.5) containing 100 mM KCl for 30 min on ice. Where indicated, sodium azide (NaN_3, 10 mM) was added and allowed to equilibrate for 30 min on ice prior to irradiation. Samples were photo-irradiated at 455 nm for 30 sec (G4-Ru-loop and G4-Ru-term) or 10 min (G4-BDP-loop and G4-BDP-term). After Cys alkylation with iodoacetamide (IAM, 10 mM), labeled proteins were visualized by DBCO-Cy3 click reaction (100 µM, 1 h, 37 °C) and SDS-PAGE (12% gel). Lane 1: no irradiation, no NaN_3; Lane 2: irradiation, no NaN_3; Lane 3: irradiation with 10 mM NaN_3. Upper panels, fluorescence imaging (Cy3 channel); lower panels, Coomassie Brilliant Blue (CBB) staining confirming equal protein loading. Labeled UP1 band is indicated by an arrow.

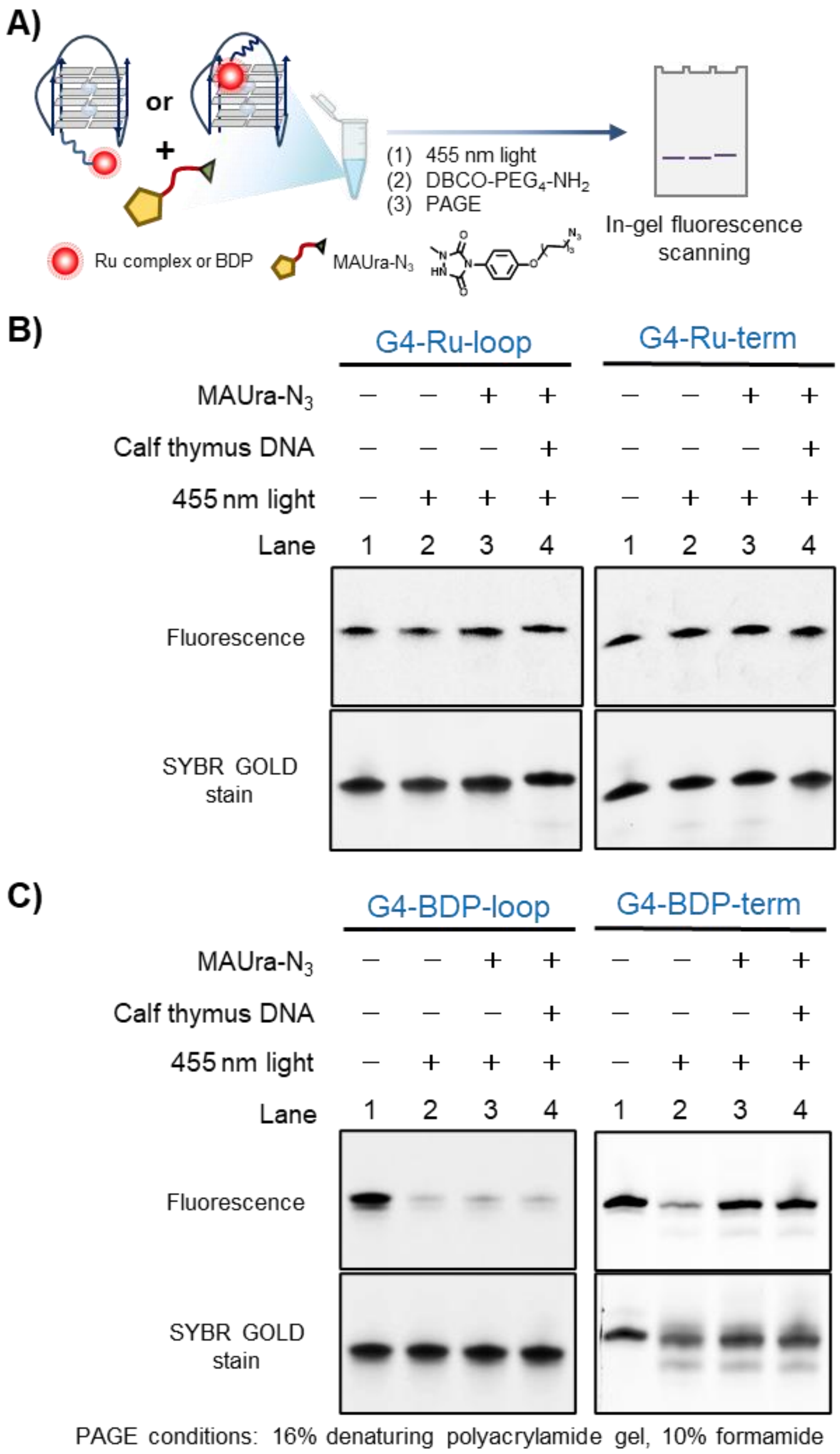

Figure S13. Investigating the G4-cat photolabeling. A) Schematic workflow of the labeling reaction. **B**) G4-Ru gel images **C**) G4-BDP gel images. Reaction conditions: G4-cat [5.0 µM], MAUra-N3 [0.5 mM], calf thymus DNA [1 ng/µL], in 50 mM Tris-HCl buffer (pH 7.5) containing KCl [100 mM]. 455 nm LED light, 30 sec (Ru) 10 min (BDP), 0 ℃.

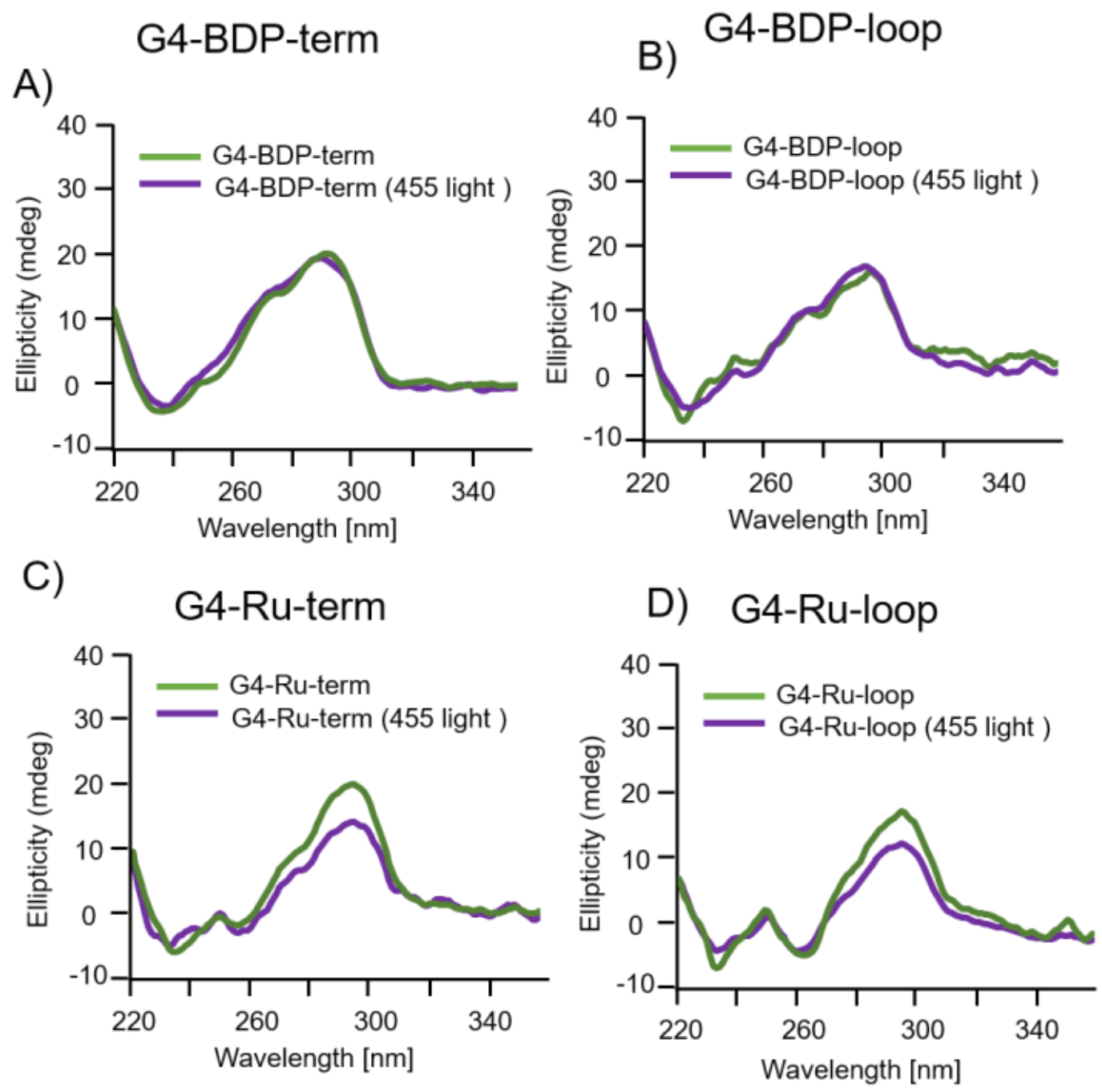

Figure S14. CD spectroscopy confirms preservation of G4 structure after photo-irradiation. CD spectra of each G4-conjugated photosensitizer probe (1.0 µM in 50 mM Tris-HCl, pH 7.5, 100 mM KCl) were recorded at 20 °C before and after photo-irradiation at 455 nm. MAUra (100 µM) was added before irradiation to replicate the labeling conditions. After irradiation, MAUra was removed by ultrafiltration (Amicon Ultra 0.5 mL 3K, Millipore) with buffer exchange to 50 mM Tris-HCl (pH 7.5) containing 100 mM KCl before CD measurement. Irradiation times: 30 sec for G4-Ru-loop and G4-Ru-term; 10 min for G4-BDP-loop and G4-BDP-term. Panels show A) G4-BDP-term, B) G4-BDP-loop, C) G4-Ru-term, and D) G4-Ru-loop. Green lines, before irradiation; purple lines, after irradiation.

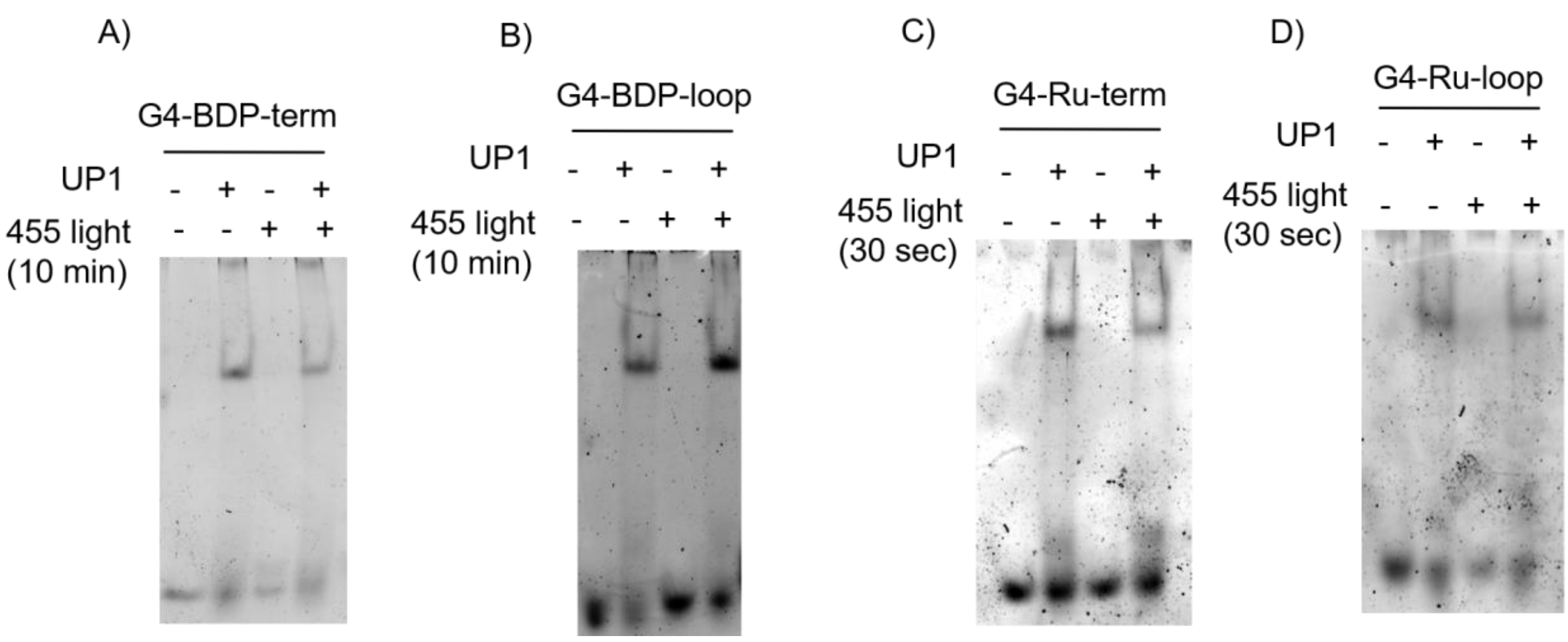

Figure S15. EMSA confirms that UP1 binding to G4 is retained after photo-irradiation. A) G4-BDP-term, B) G4-BDP-loop, C) G4-Ru-term, and D) G4-Ru-loop (50 nM each, in 50 mM Tris-HCl, pH 7.5, 100 mM KCl) were annealed and mixed with MAUra (100 µM). Samples were photo-irradiated at 455 nm for 30 sec (G4-Ru-loop and G4-Ru-term) or 10 min (G4-BDP-loop and G4-BDP-term), or kept on ice as non-irradiated controls. EMSA binding reactions were performed in 20 µL [50 mM Tris-HCl (pH 7.5), 100 mM KCl] containing UP1 (0.5 µM) and irradiated or non-irradiated G4 (50 nM) for 30 min on ice. Complexes were resolved by 6% non-denaturing PAGE (19:1 acrylamide:bis-acrylamide) in 0.5× TBE at 4 °C, 200 V, for 1 h, and visualized by SYBR Gold staining.

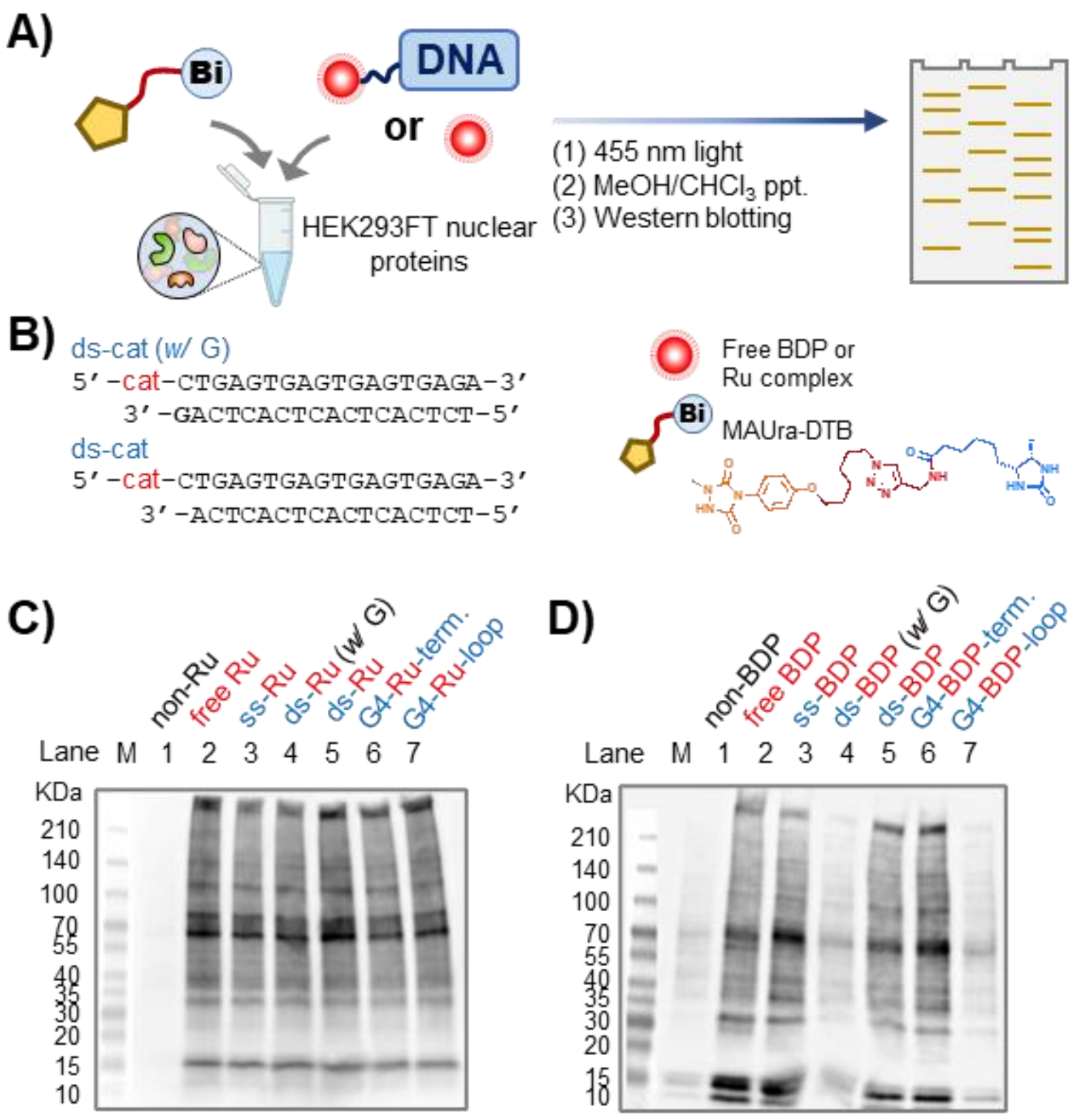

Figure S16. Evaluating the labeling reaction for catalyst-modified ss-, ds-, G4-DNA, or free catalyst with nuclear extract proteins. A) Schematic workflow of the labeling reaction. B) dsDNA-catalyst sequences. Western blotting gel image using **C**) Ru complex and **D**) BDP. Reaction conditions: DNA-cat or free cat [5.0 μM], MAUra-DTB [0.5 mM], nuclear extract [1.0 mg/mL], calf thymus DNA [1 ng/μL], in 50 mM Tris-HCl buffer (pH 7.5) containing KCl [100 mM]. 455 nm LED light for 1 min (Ru complex) or 10 min (BDP), 0 ℃.

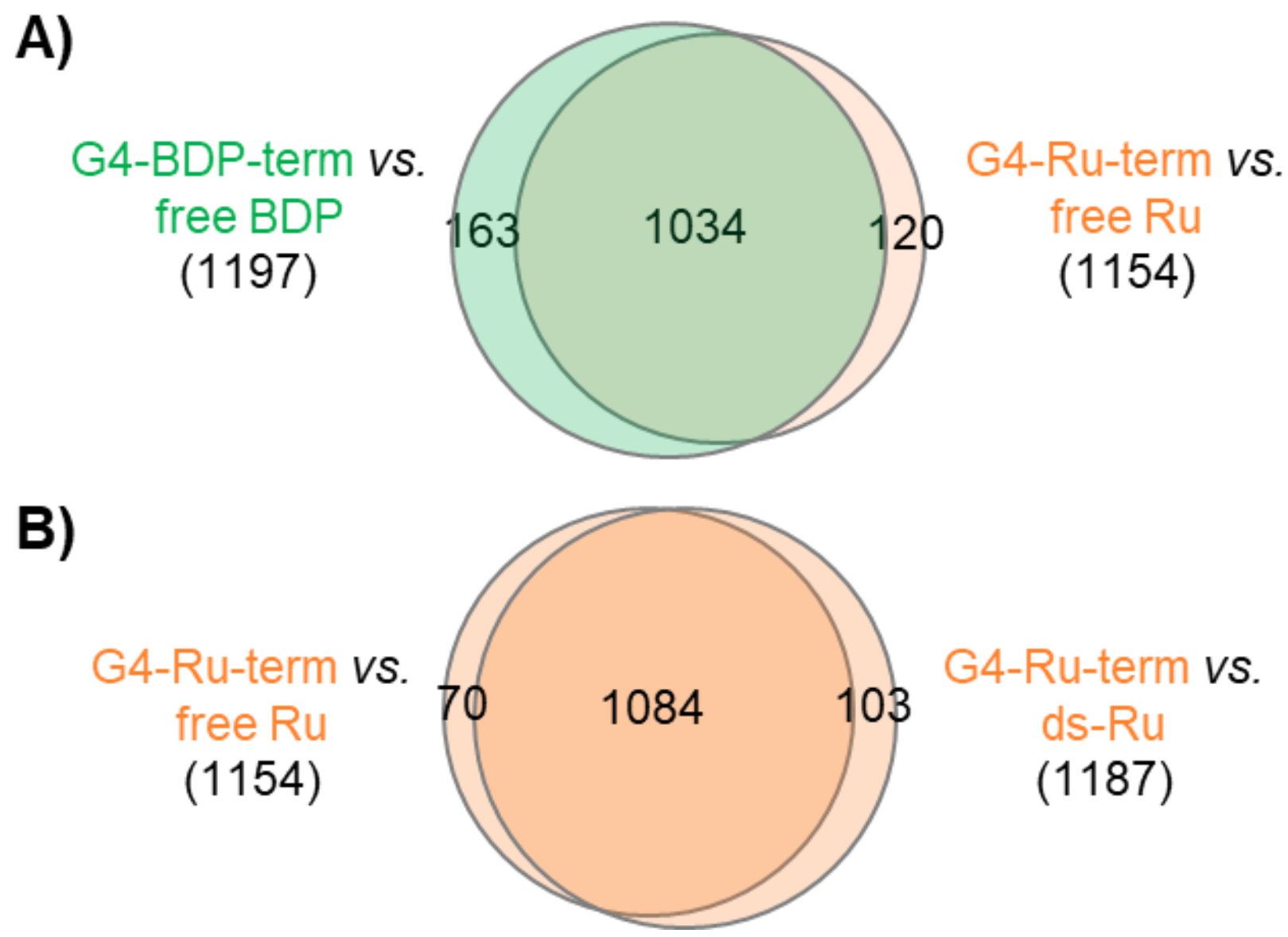

Figure S17. Overlapping between lists of enriched proteins. A) Venn diagram showing the overlapping between enriched proteins for G4-BDP-term vs. free BDP and enriched proteins for G4-Ru-term vs. free Ru. **B**) Venn diagram showing the overlapping between enriched proteins for G4-Ru-term vs. free Ru and enriched proteins vs. ds-Ru.

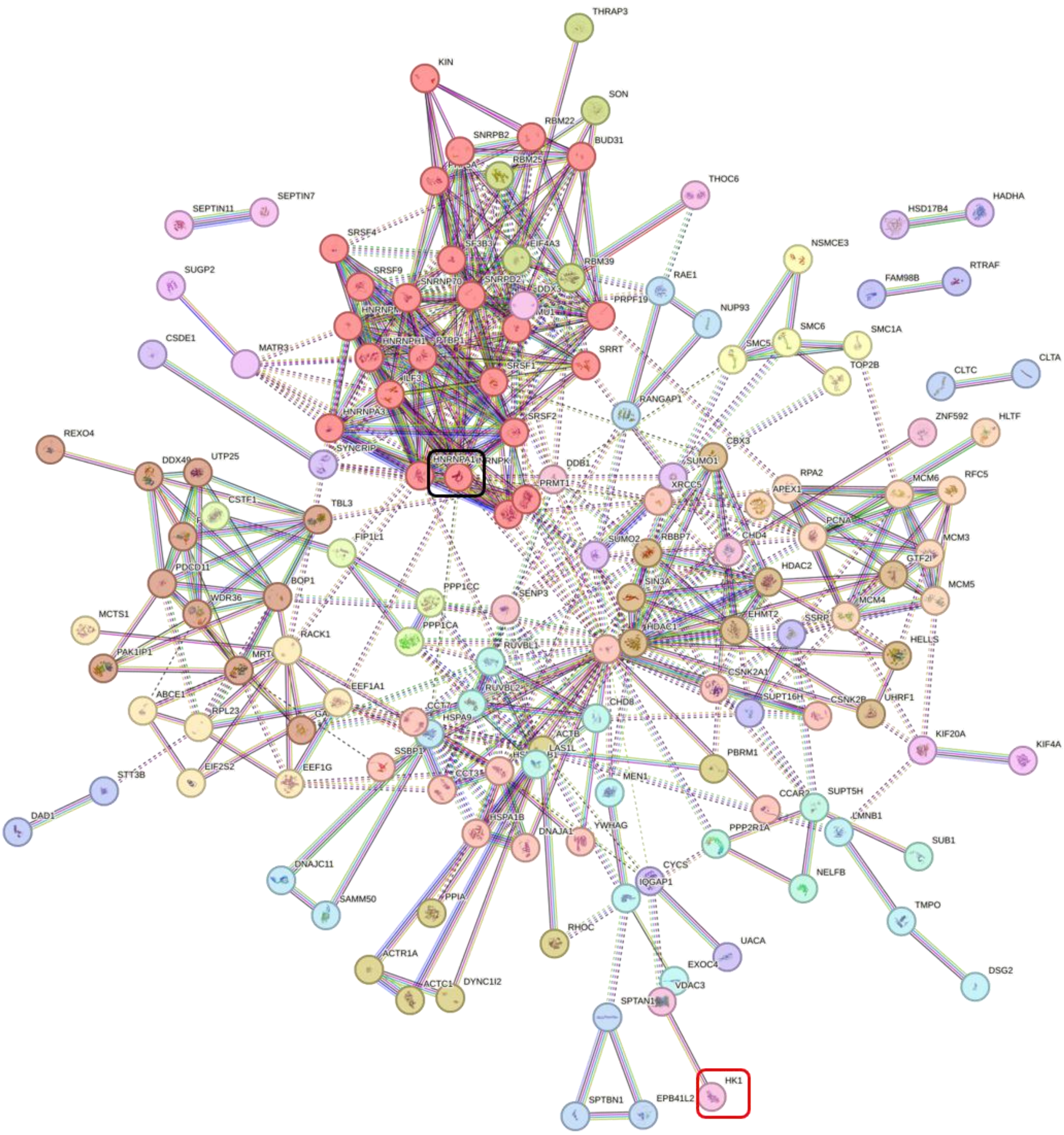

Figure S18. STRING network analysis of the identified G4-interacting proteins. The protein-protein interaction (PPI) network was analyzed using 168 proteins filtered by the criteria of -log10(*P*-value) > 4 and log2(FC) > 2.5. The nodes represent individual proteins, and the edges represent predicted functional or physical associations. The network was clustered into functional modules using the Markov Cluster Algorithm (MCL), where nodes sharing the same color belong to the same cluster. Solid lines indicate intra-cluster interactions, whereas dotted lines represent inter-cluster interactions. HK1 and HNRNPA1 are boxed by red and black color, respectively, to illustrate their topological localizations within the functional clusters.

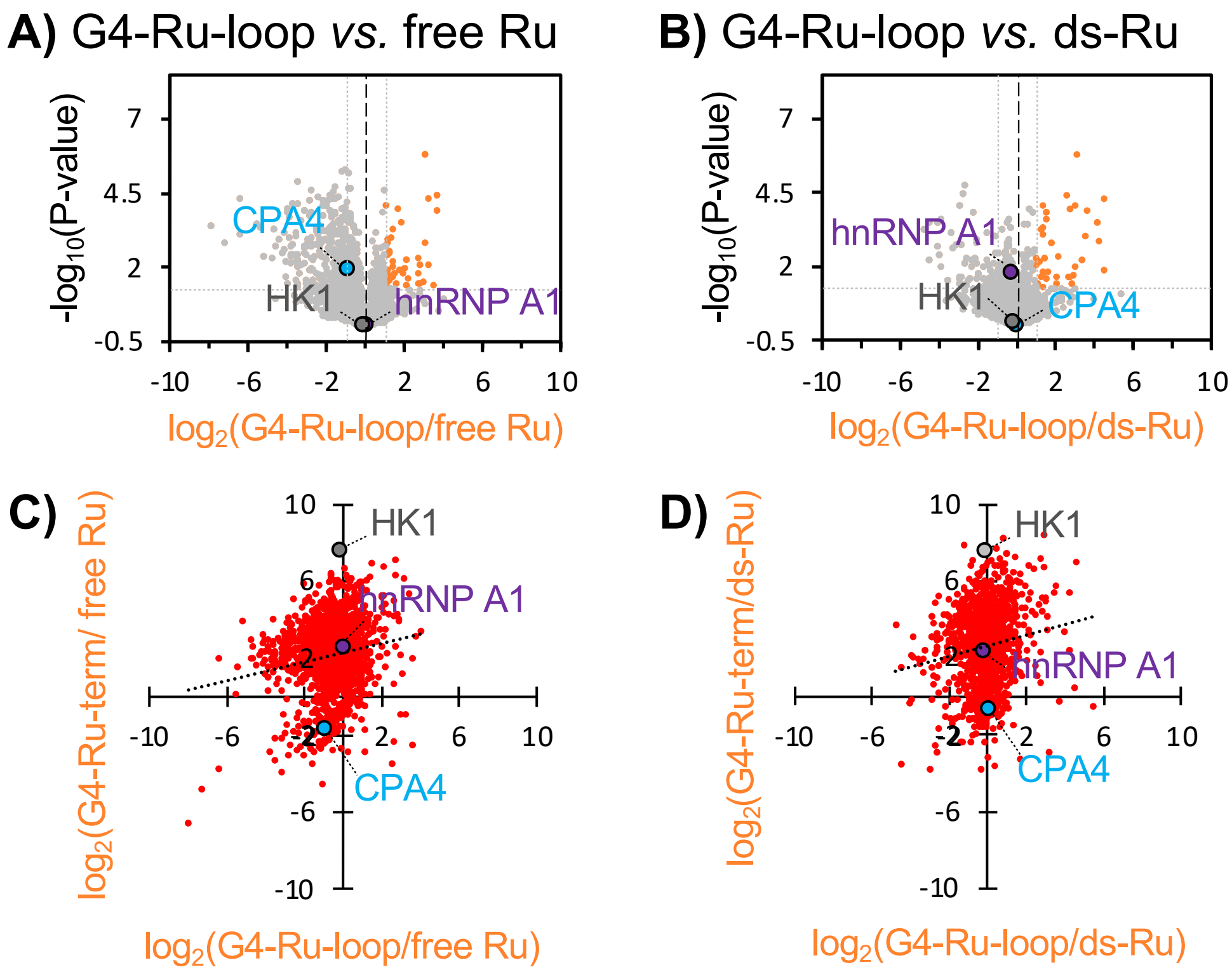

Figure S19. Investigation of G4-Ru-loop interacting proteins by LC-MS/MS-based proteomics analysis. **A**) Volcano plot displaying enriched proteins (highlighted in orange) for G4-Ru-loop versus free Ru (n=41 out of 1583). **B**) Volcano plot displaying enriched proteins (highlighted in orange) for G4-Ru-loop versus ds-Ru (n=37 out of 1573). Proteins were considered enriched with a >2-fold signal over control and a P-value <0.05. **C**) The correlation between G4-Ru-loop and G4-Ru-term proteins versus free Ru. **D**) The correlation between G4-Ru-loop and G4-Ru-term proteins versus ds-Ru. The heterogeneous nuclear ribonucleoprotein A1 (hnRNP A1) protein (as a known G4IP), Carboxypeptidase A4 (CPA4) protein (as negative control protein candidate), and Hexokinase-1 (HK1) protein (as unknown G4IP candidate) were noted in A–D in purple, blue and gray respectively.

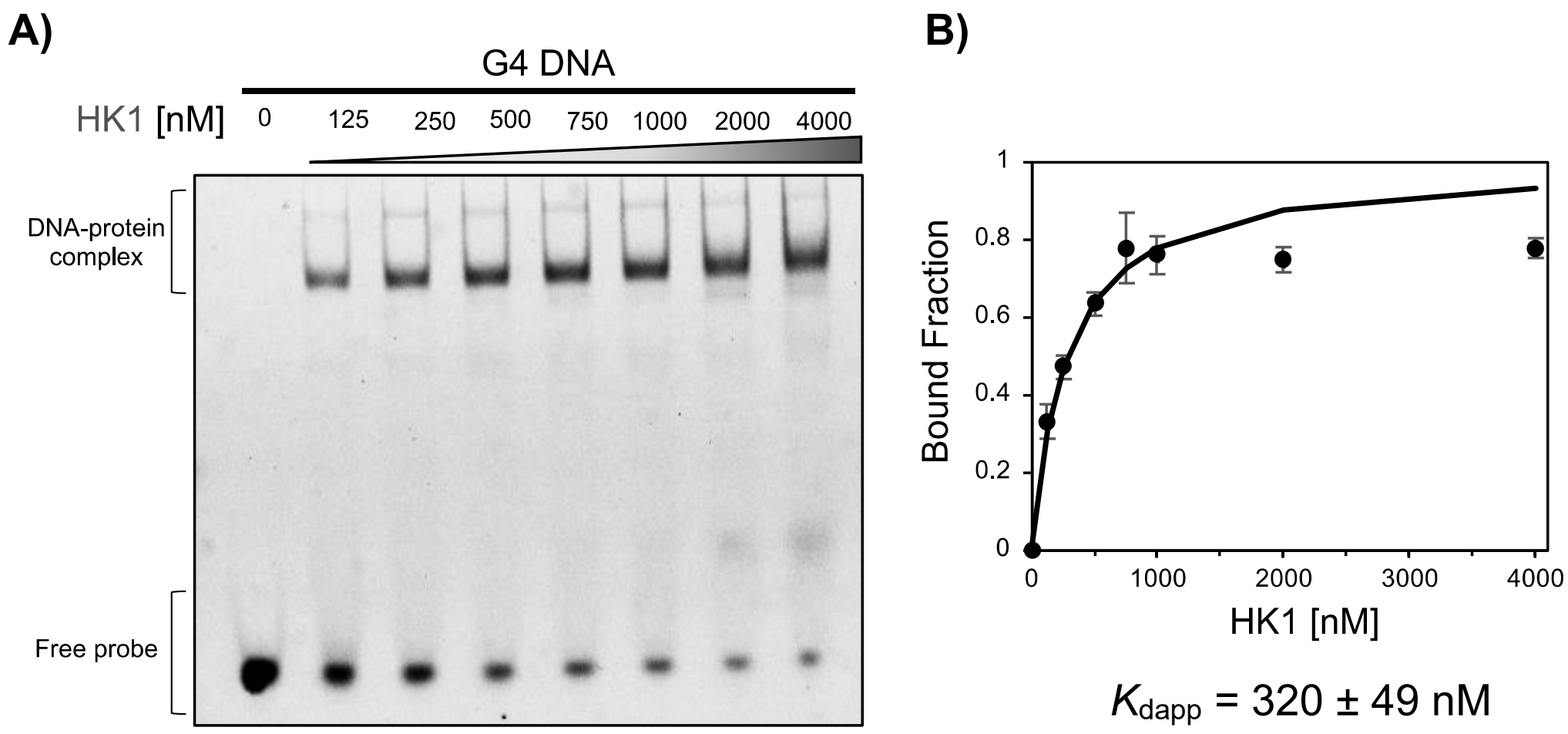

Figure S20. K_d value measurement for HK1 protein using EMSA with FAM-labeled G4 DNA (2 nM) sequence in the presence of calf thymus DNA (1 ng/µL). (A) Representative EMSA showing the concentration-dependent binding of HK1 to G4 DNA. (B) Non-linear curve fitting of the bound fraction used to calculate the apparent K_d.

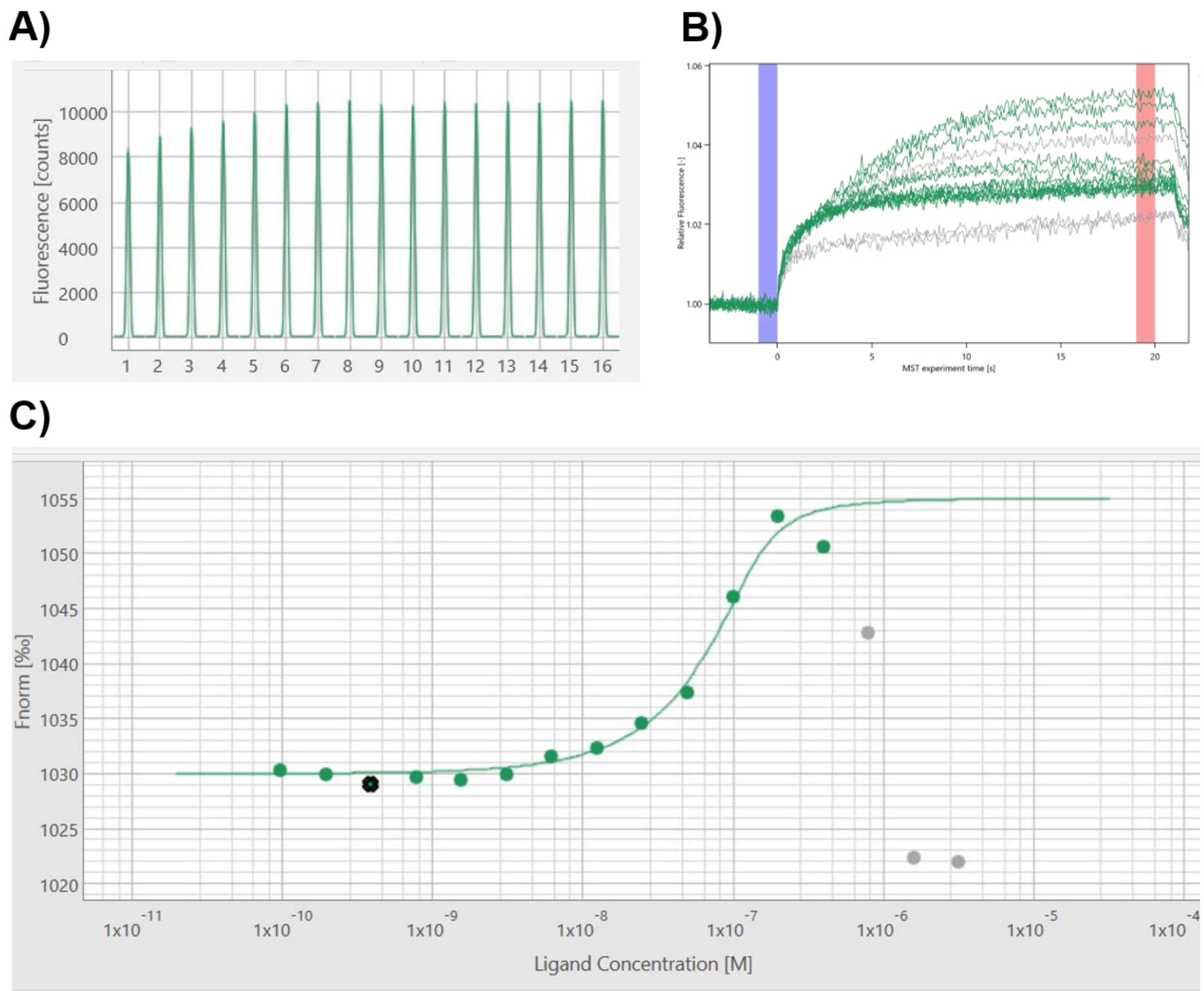

Figure S21. Microscale thermophoresis (MST) determination of the binding affinity of HK1 for G4. Binding of HK1 to the human telomeric G4 sequence (HT G4; 5′-d(TAGGGTTAGGGTTAGGGTTAGGG)-3′) labeled at the 3′ end with Texas Red (HT-Texas Red, 20 nM) was measured by MST. HK1 was serially diluted in MST assay buffer (10 mM Tris-HCl, pH 7.5, 20 mM KCl, 0.05% Tween-20) and titrated from 3.2 µM to 0.1 nM (16 two-fold dilutions). (A) Capillary fluorescence scan showing the initial fluorescence intensity across all 16 capillaries. (B) Raw MST traces. (C) The normalized fluorescence (Fnorm) is plotted as a function of HK1 concentration. The data were fitted to a standard 1:1 binding model. Data points at HK1 concentrations ≥400 nM were excluded from fitting owing to fluorescence quenching of Texas Red at high protein concentrations, as evidenced by a consistent decrease in both the capillary fluorescence intensity and Fnorm values. The reported K_d = 11.8 ± 2.0 nM represents the mean ± SD from three independent experiments.

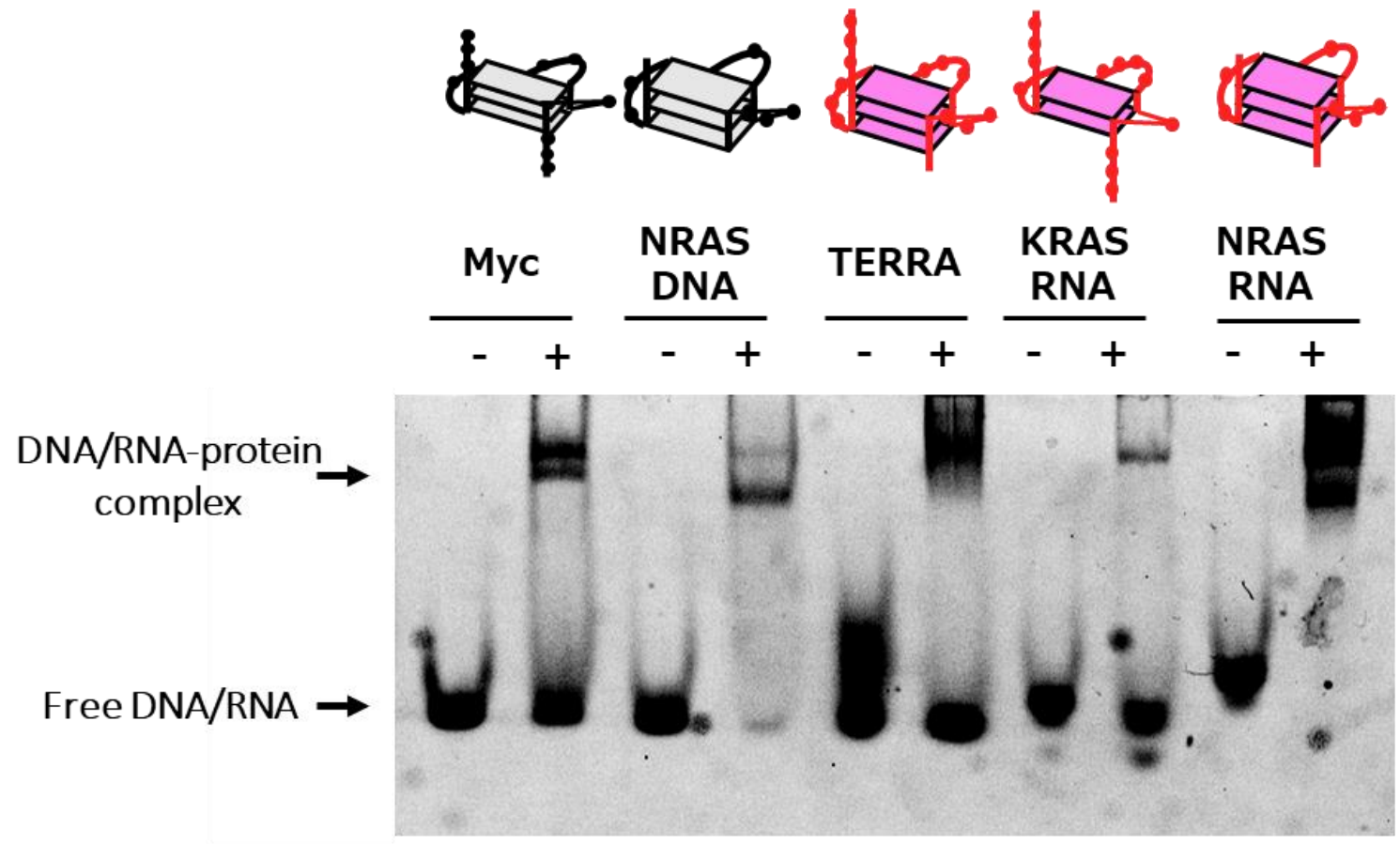

Myc G4 DNA
5′-FAM-TGA**GGG**T**GGG**TA**GGG**T**GGG**TAA-3′
NRAS G4 DNA
5′-FAM-**GGG**A**GGGG**C**GGG**TCT**GGG**-3′
TERRA G4 RNA
5′-FAM-UA**GGG**UUA**GGG**UUA**GGG**UUA**GGG**-3′
KRAS G4 RNA
5′-FAM-**GG**C**GG**C**GG**CAGU**GG**C**GG**C**GG**-3′
NRAS G4 RNA
5′-FAM-**GGG**A**GGGG**C**GGG**UCU**GGG**-3′

Figure S22. Binding properties of HK1 protein towards selected G4-forming DNA and RNA sequences by EMSA. The EMSA of HK1 protein (1 μM) with FAM-labeled G4 DNA/RNA (5 nM) sequences was investigated in the presence of calf thymus DNA (1 ng/μL).

3. NMR-spectra

Compound **2**

^{1}H NMR (600 MHz, $CDCl_3$)

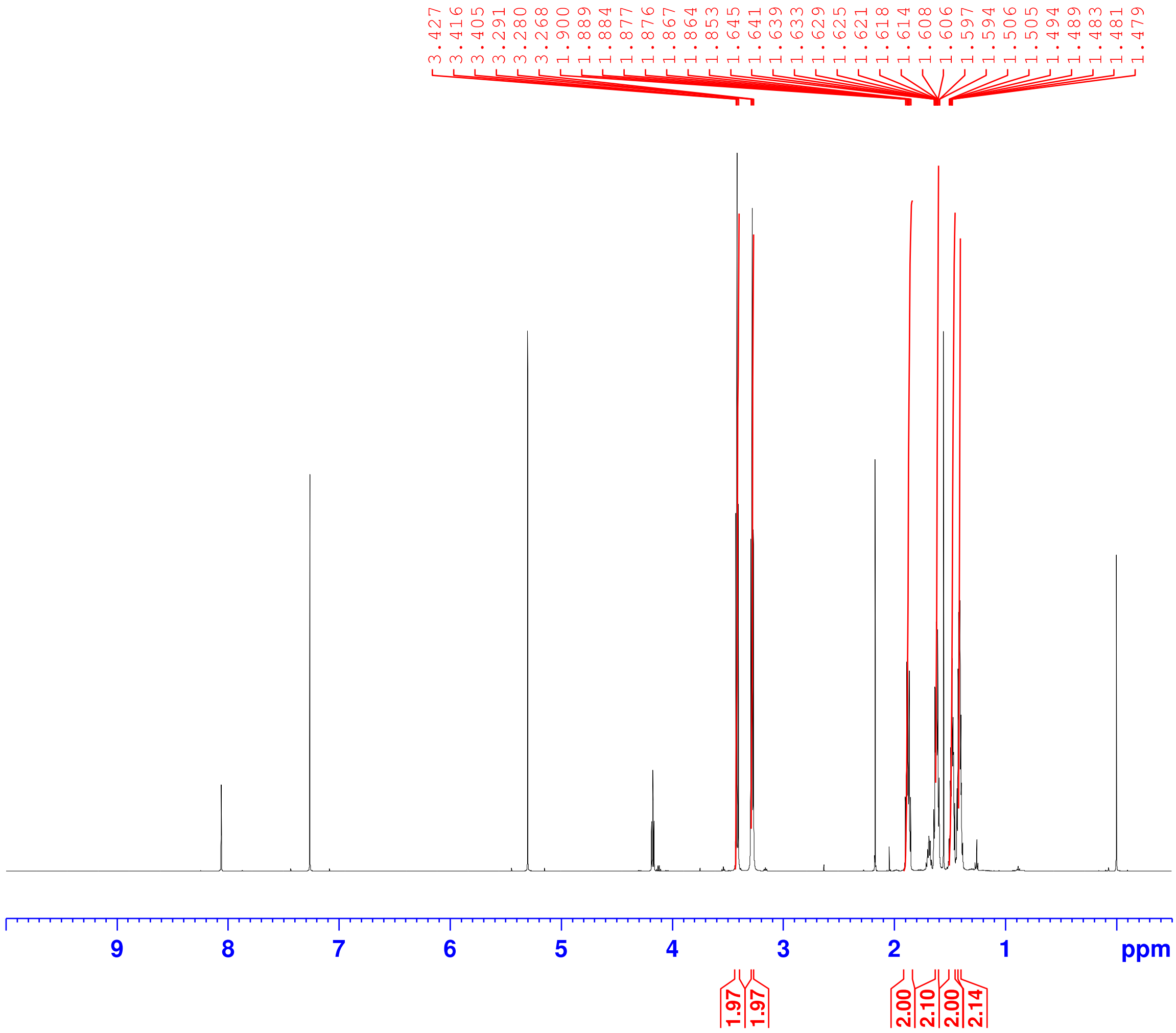

Compound **3**

^{1}H NMR (400 MHz, $CDCl_3$)

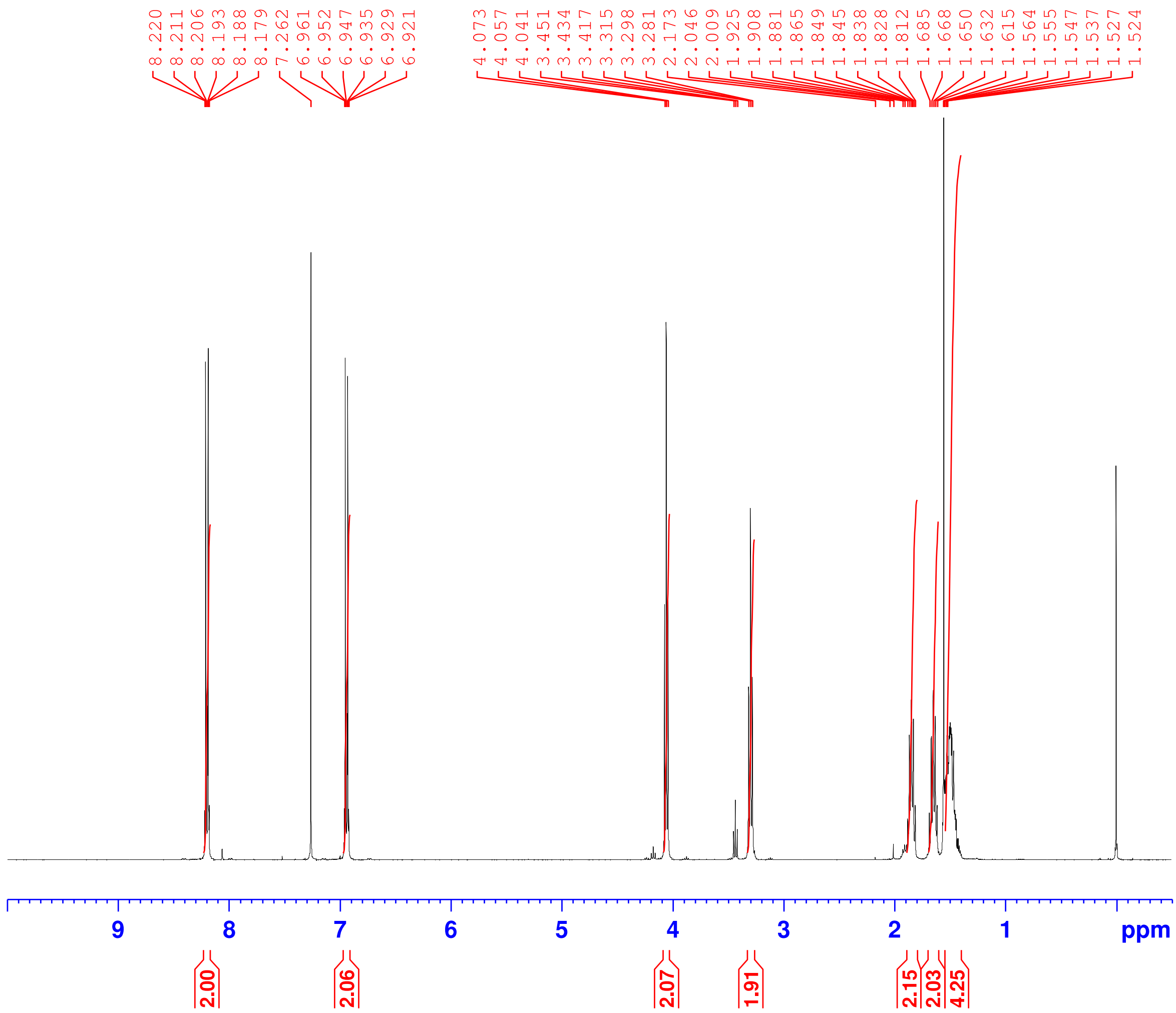

Compound **4**

^{1}H NMR (600 MHz, $CDCl_3$)

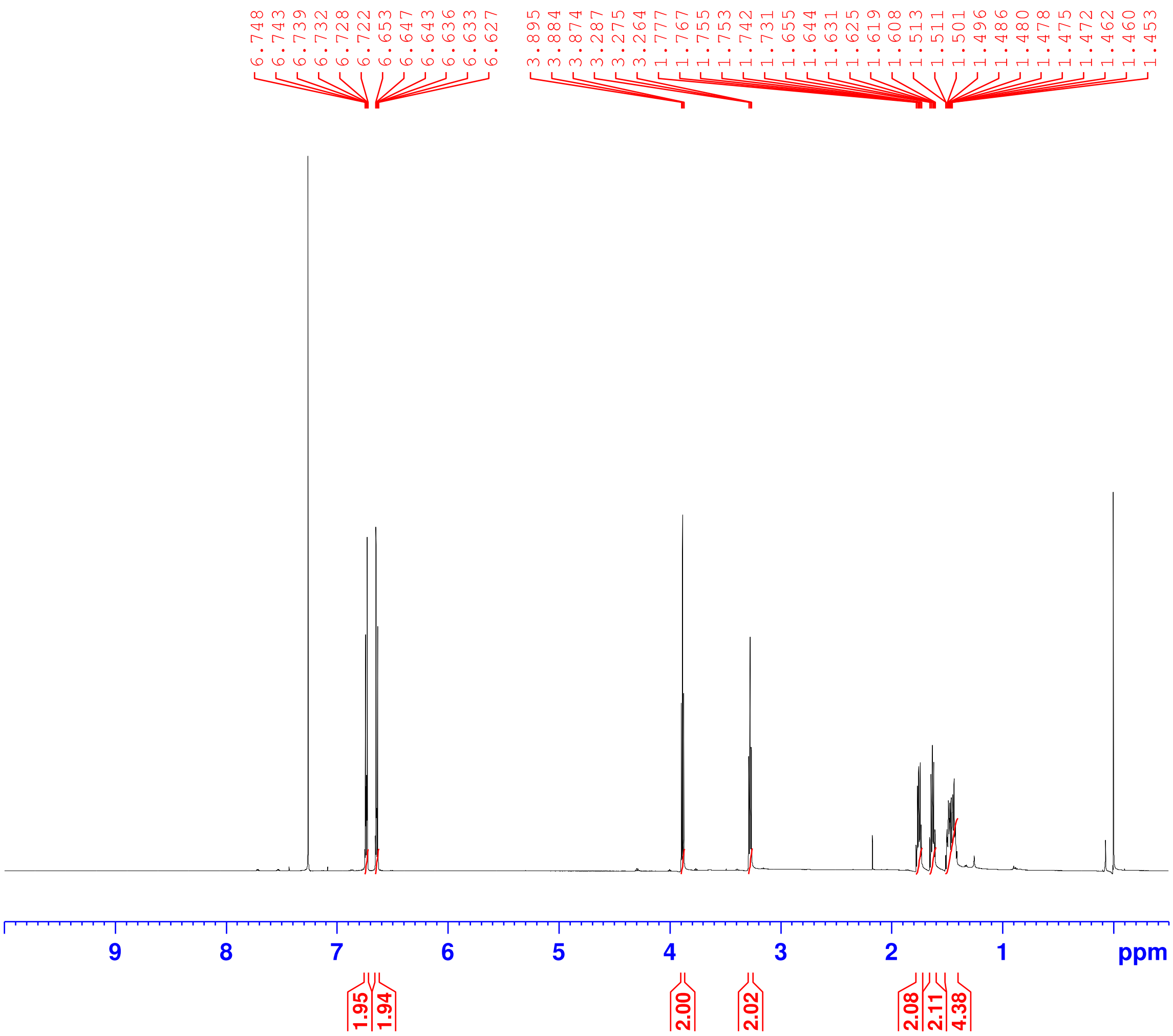

Compound **4**

$^{13}C\{^1H\}$ NMR (150 MHz, $CDCl_3$)

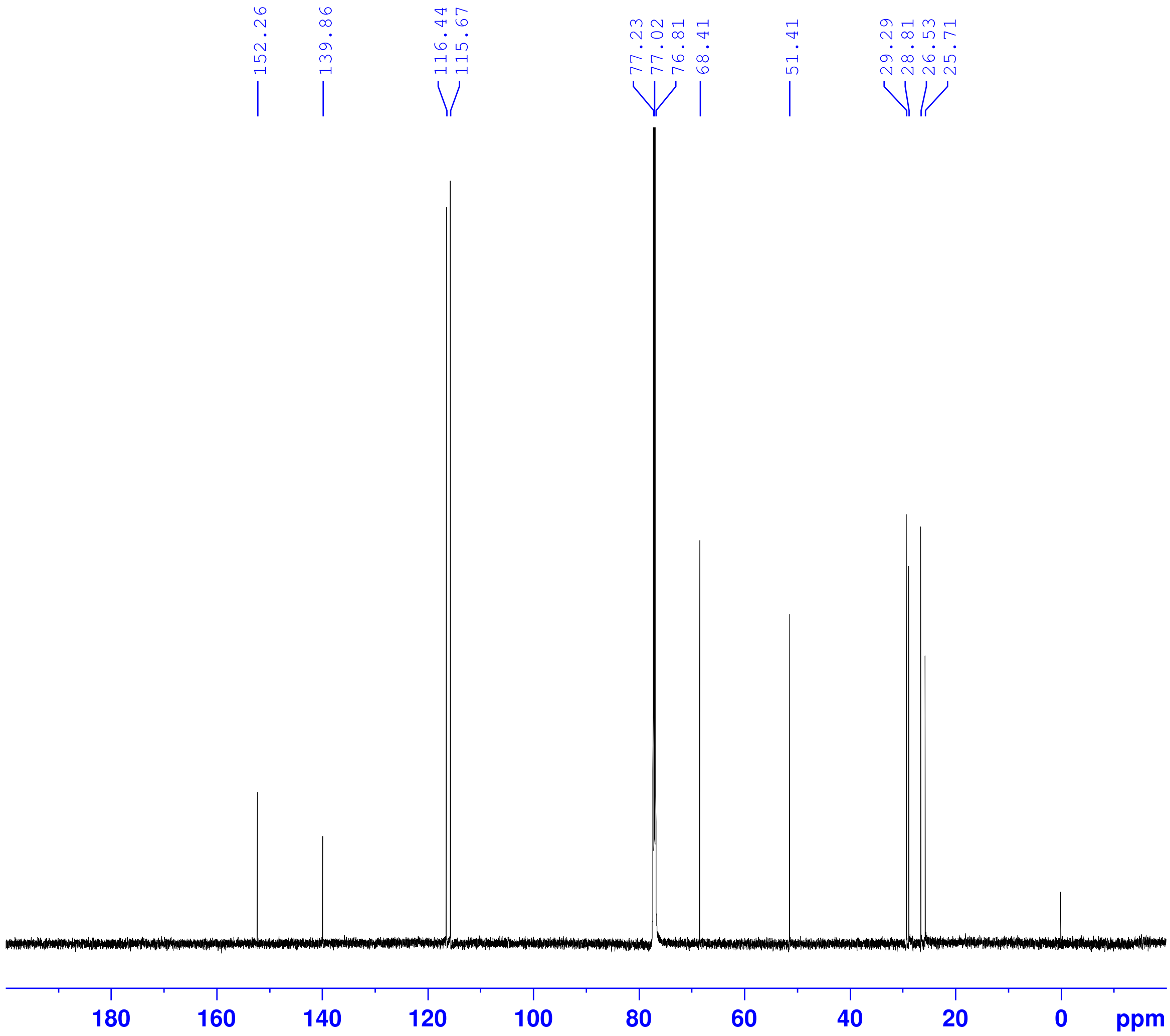

Compound **7**

^{1}H NMR (600 MHz, $CDCl_3$)

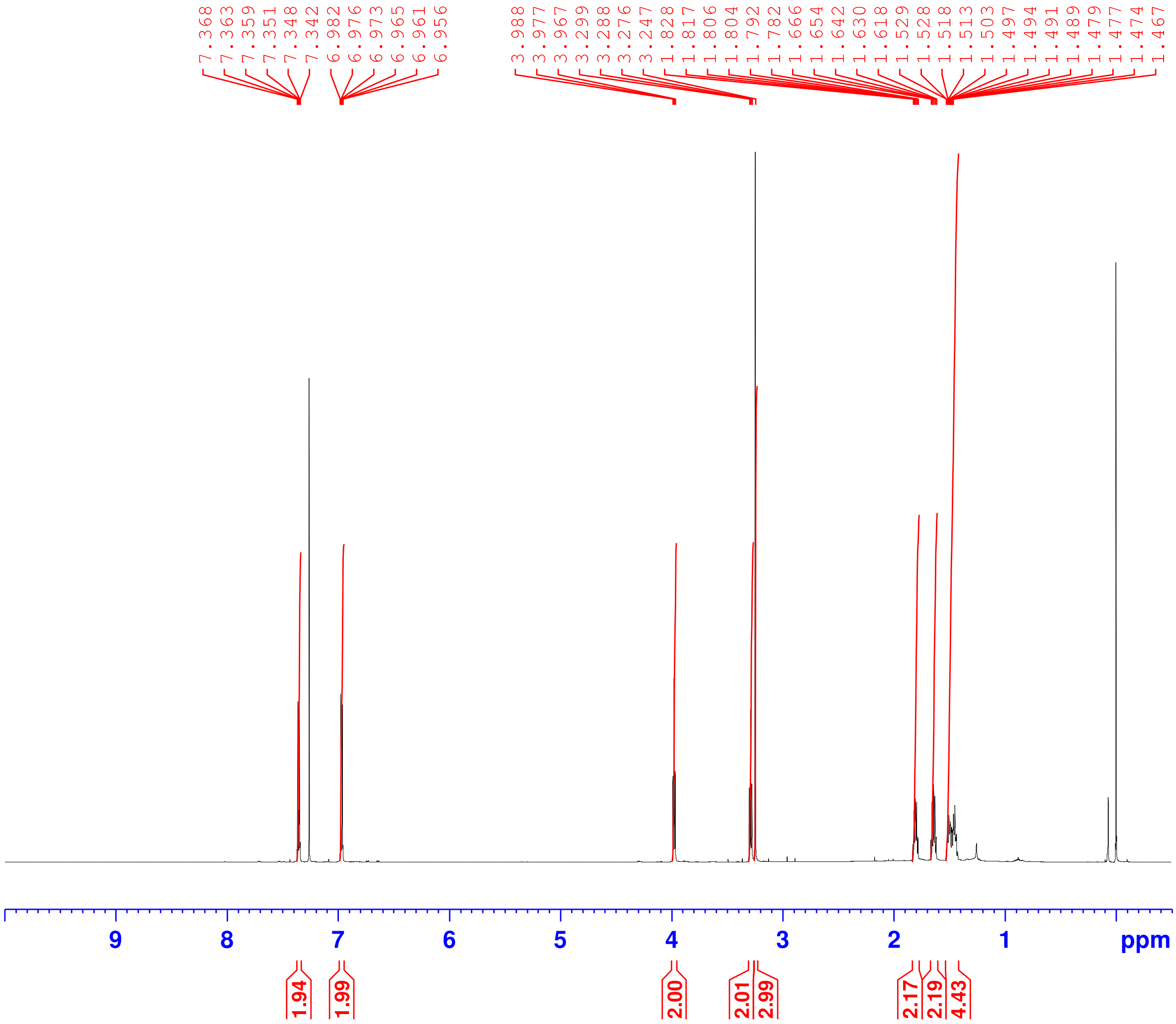

Compound **7**

$^{13}C\{^{1}H\}$ NMR (150 MHz, $CDCl_3$)

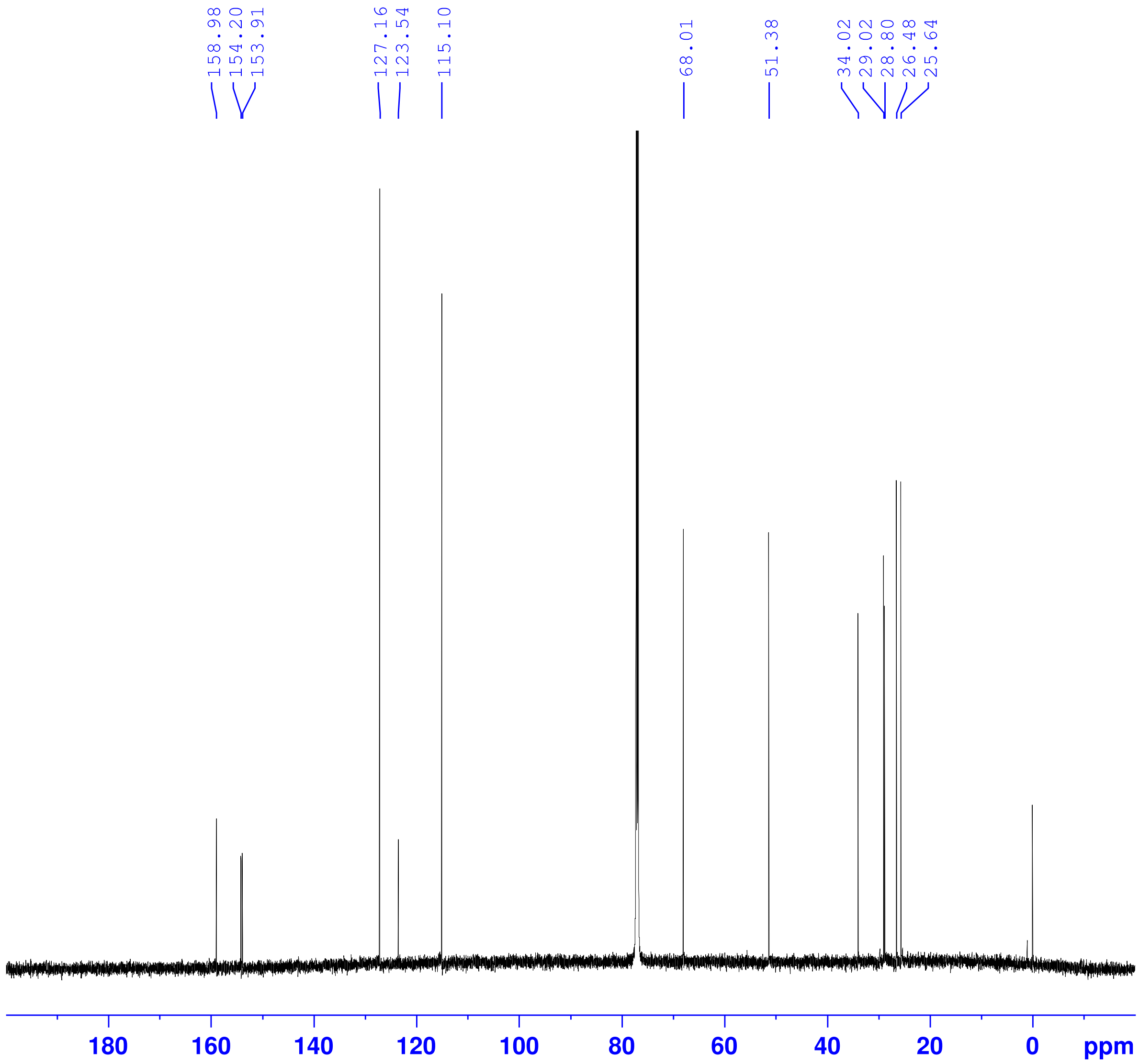

Compound **9**

^{1}H NMR (400 MHz, CD_3OD)

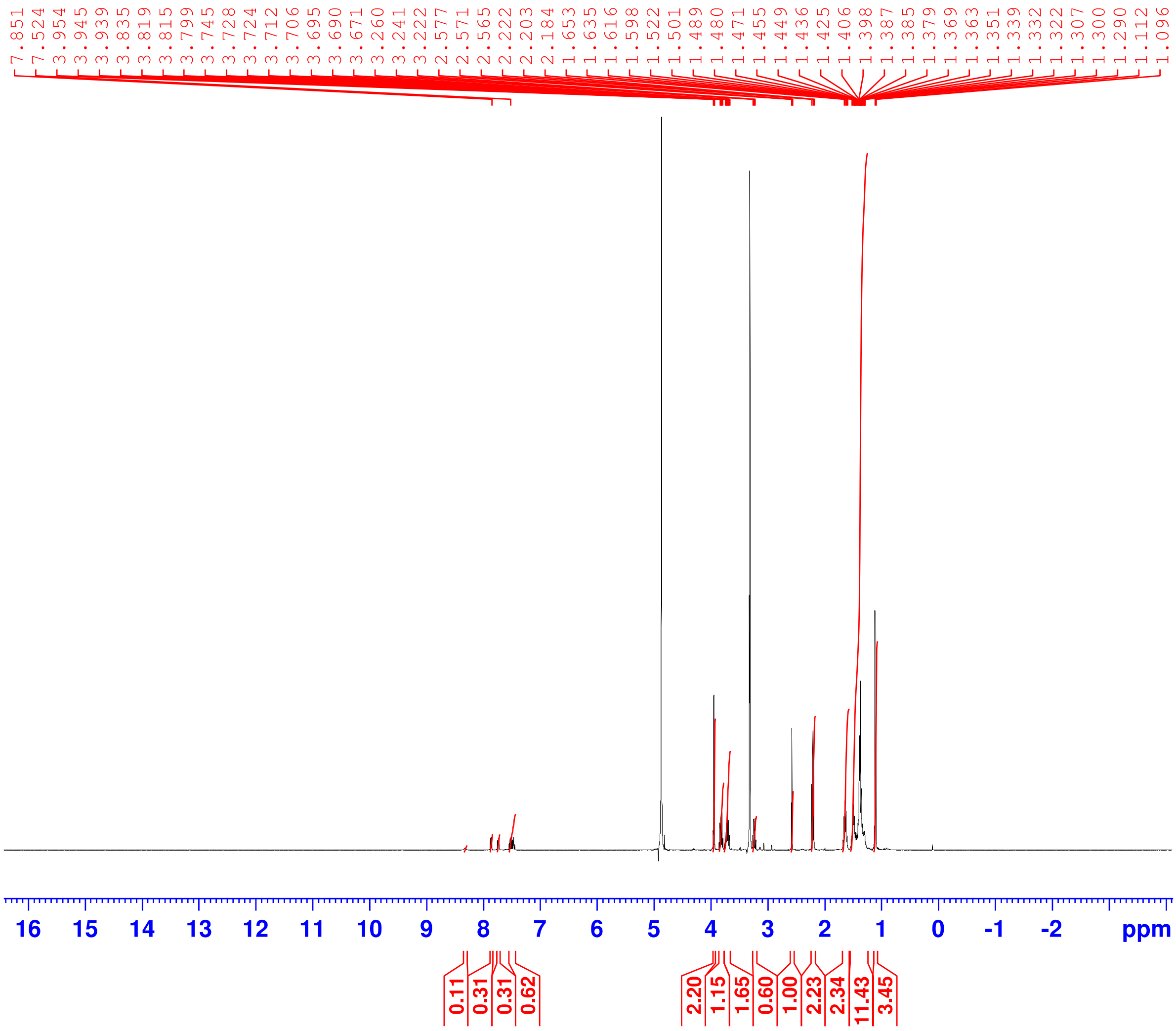

Compound **10**

^{1}H NMR (600 MHz, CD_3OD)

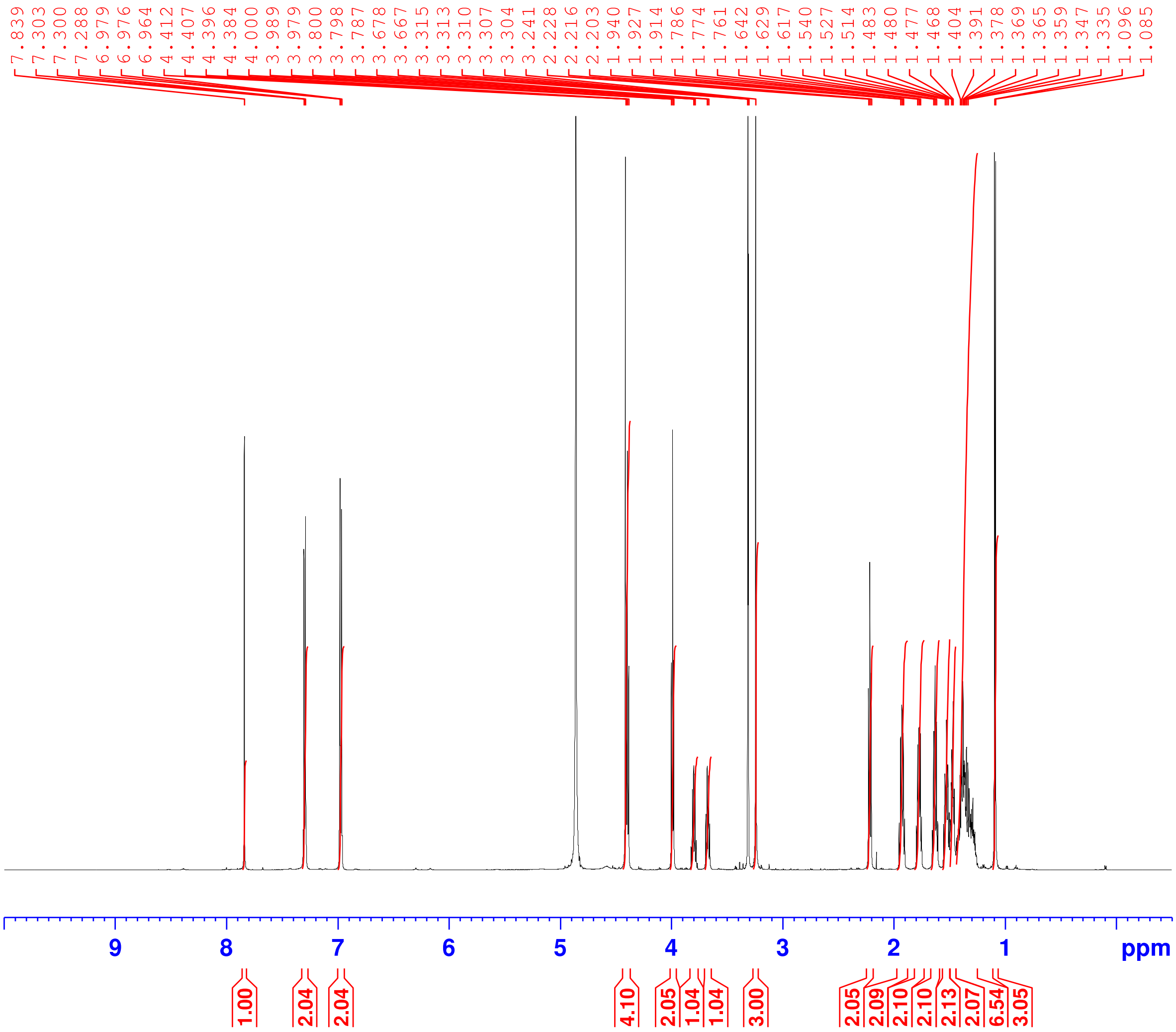

Compound **10**

$^{13}C\{^{1}H\}$ NMR (150 MHz, CD_3OD)

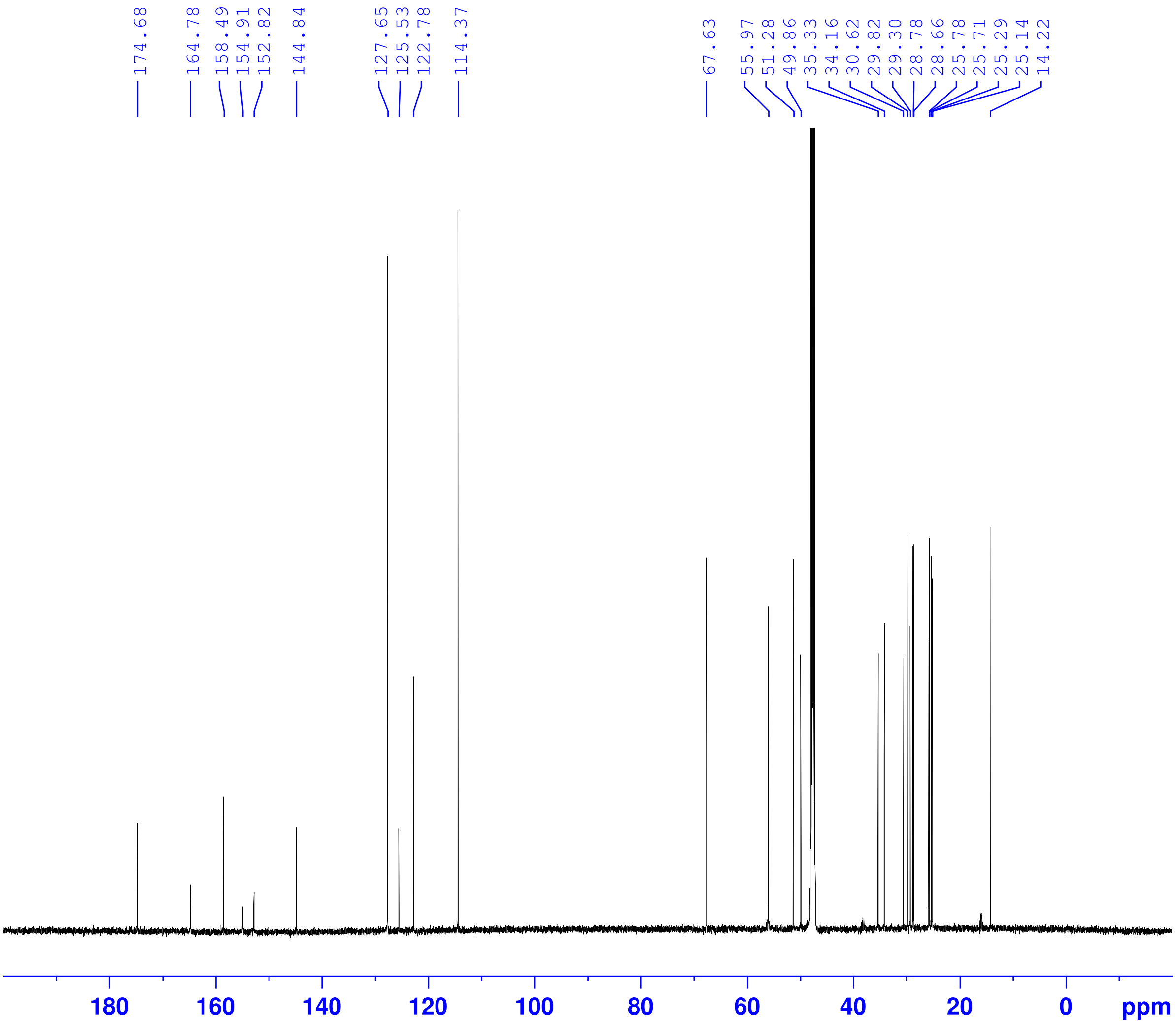

Compound **10**

qNMR (600 MHz, DMSO-d_6)

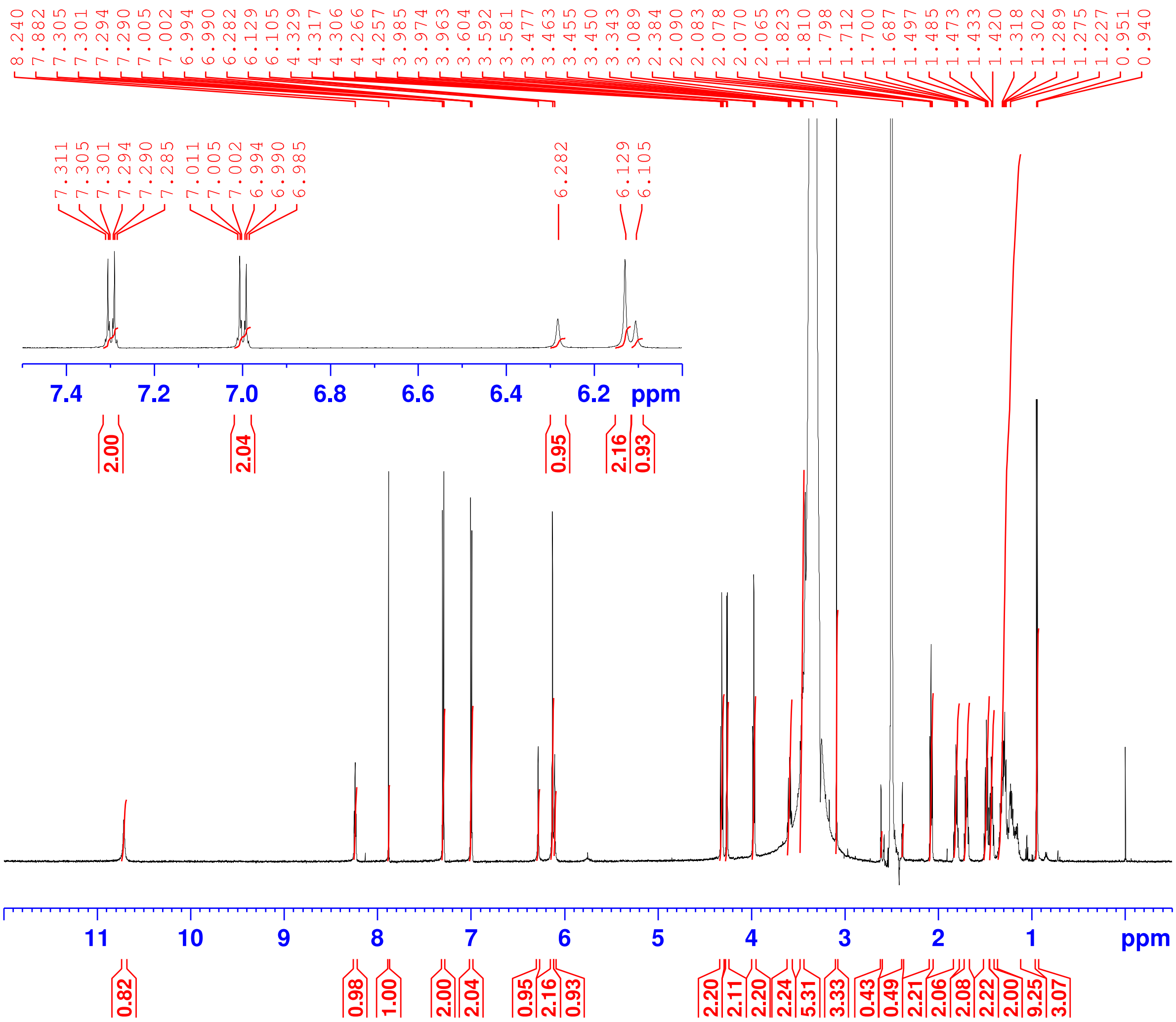

4. MALDI-TOF MS data

G4-Ru-loop

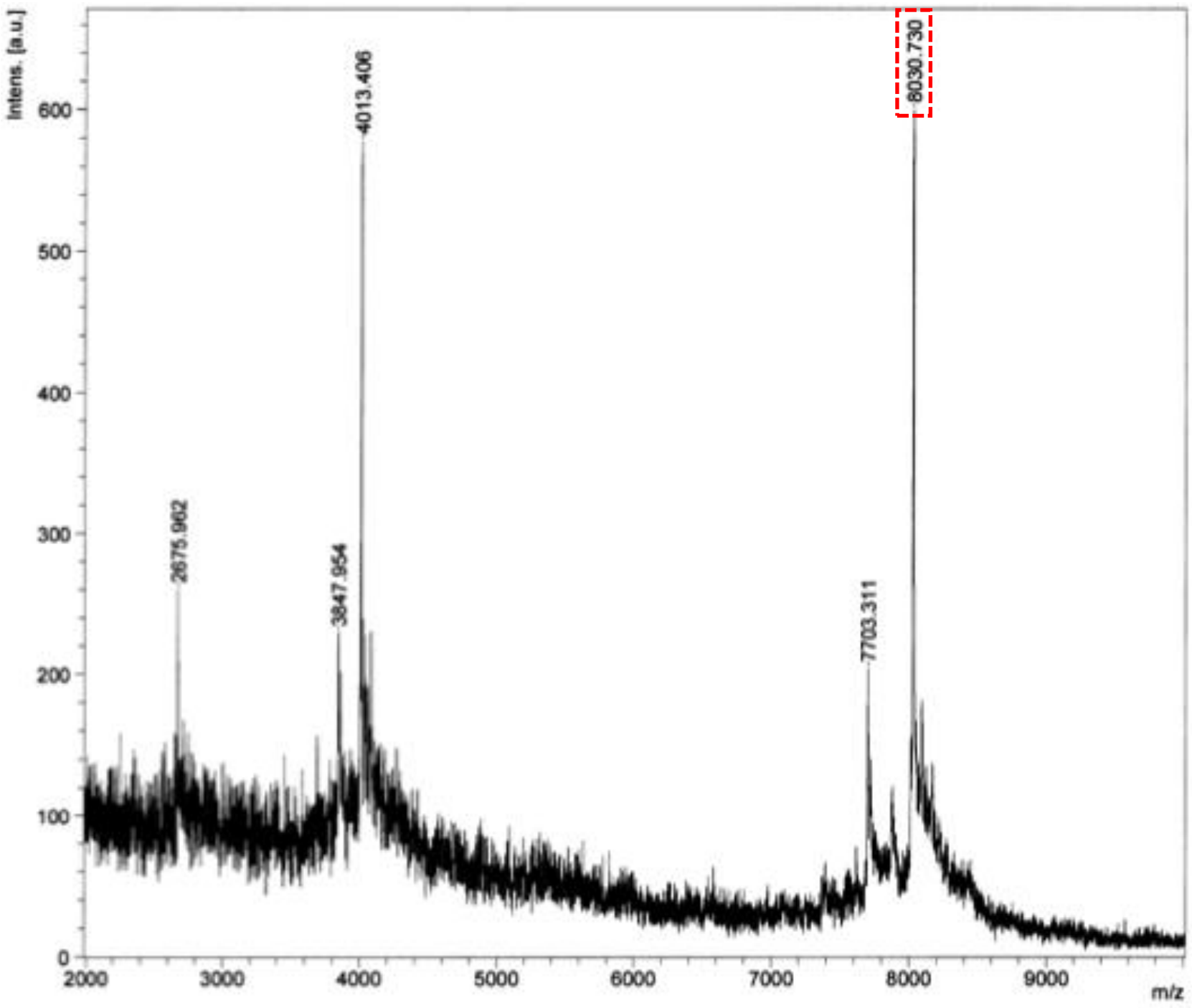

G4-Ru-term

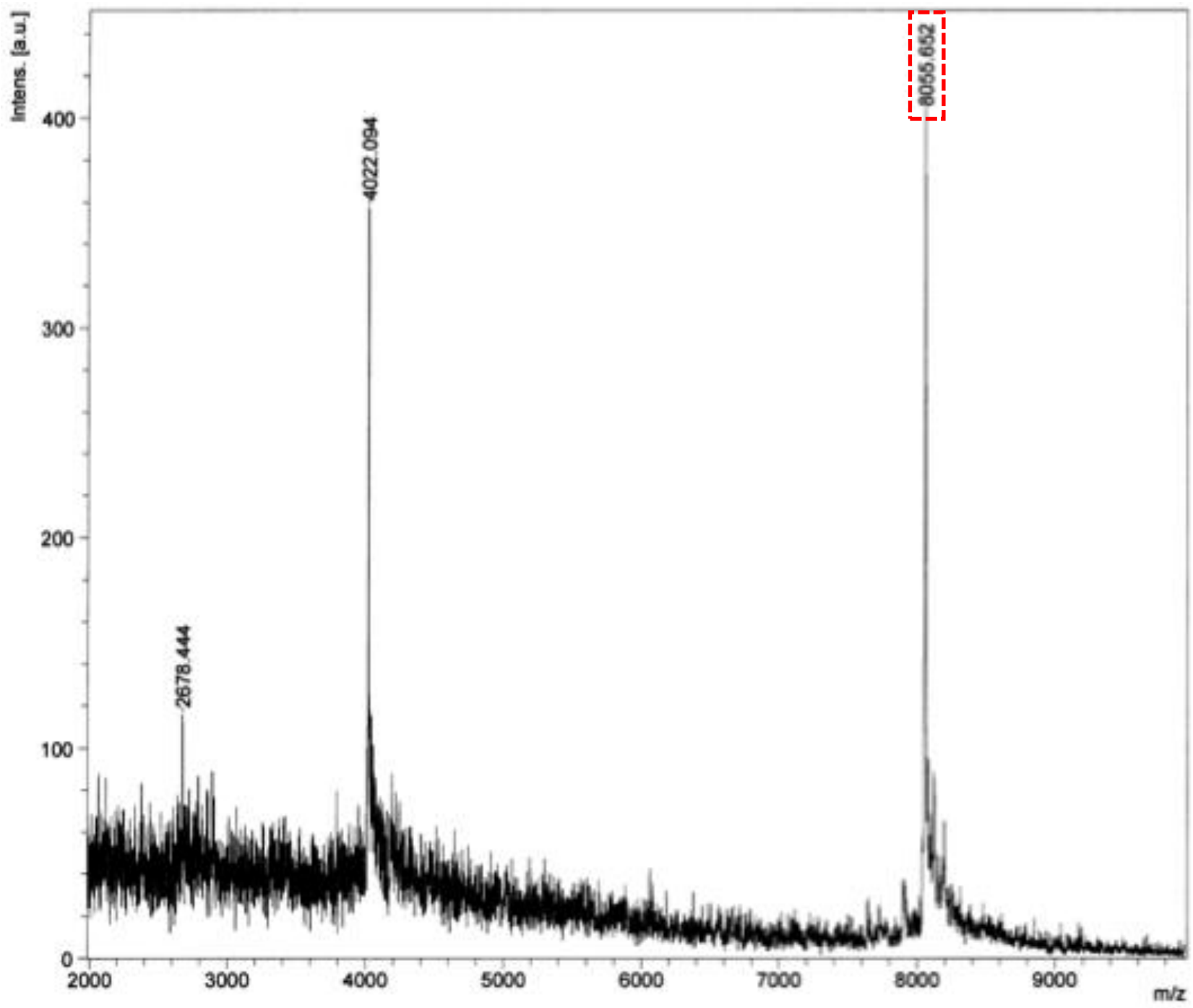

ss-Ru

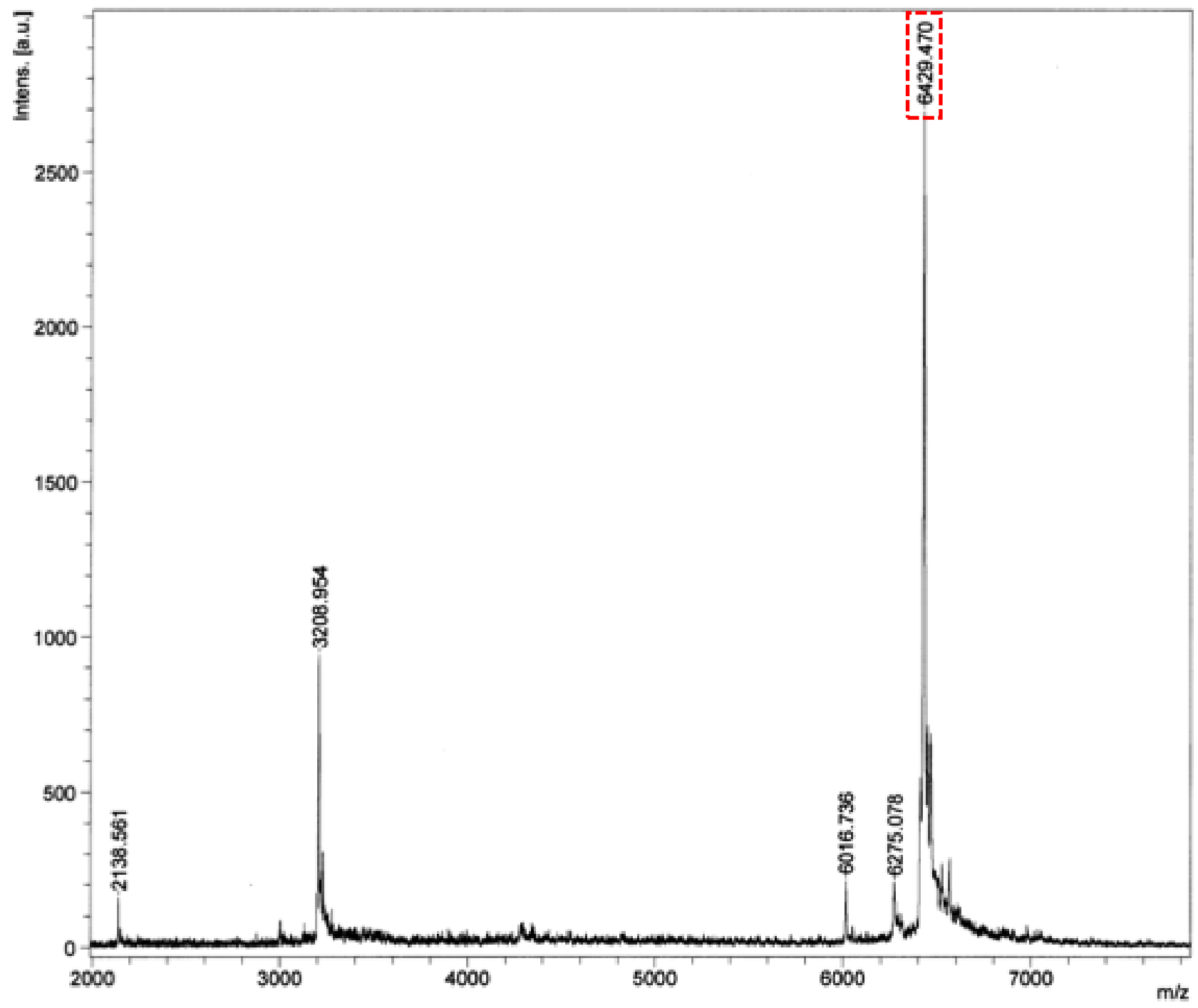

G4-BDP-loop

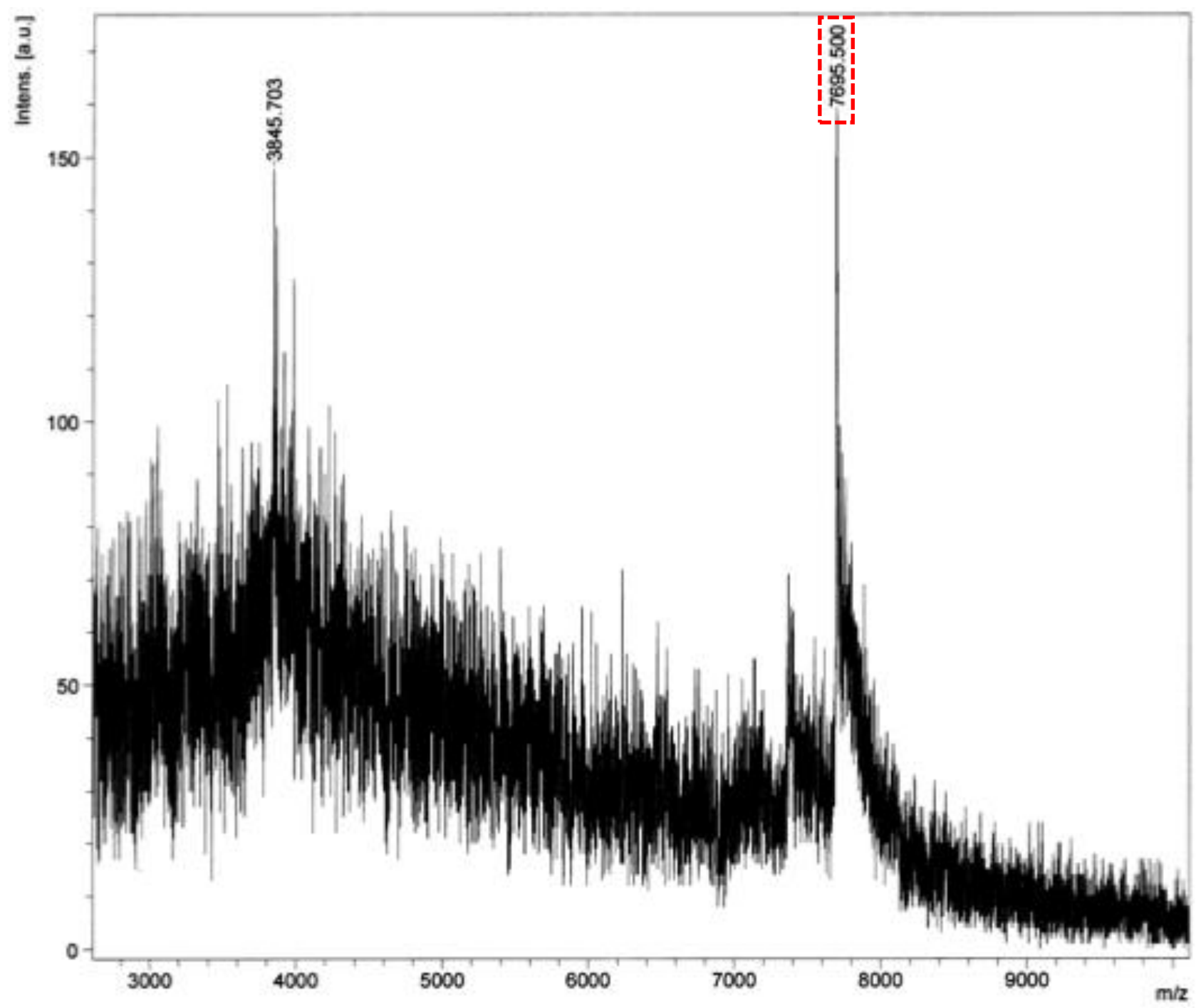

G4-BDP-term

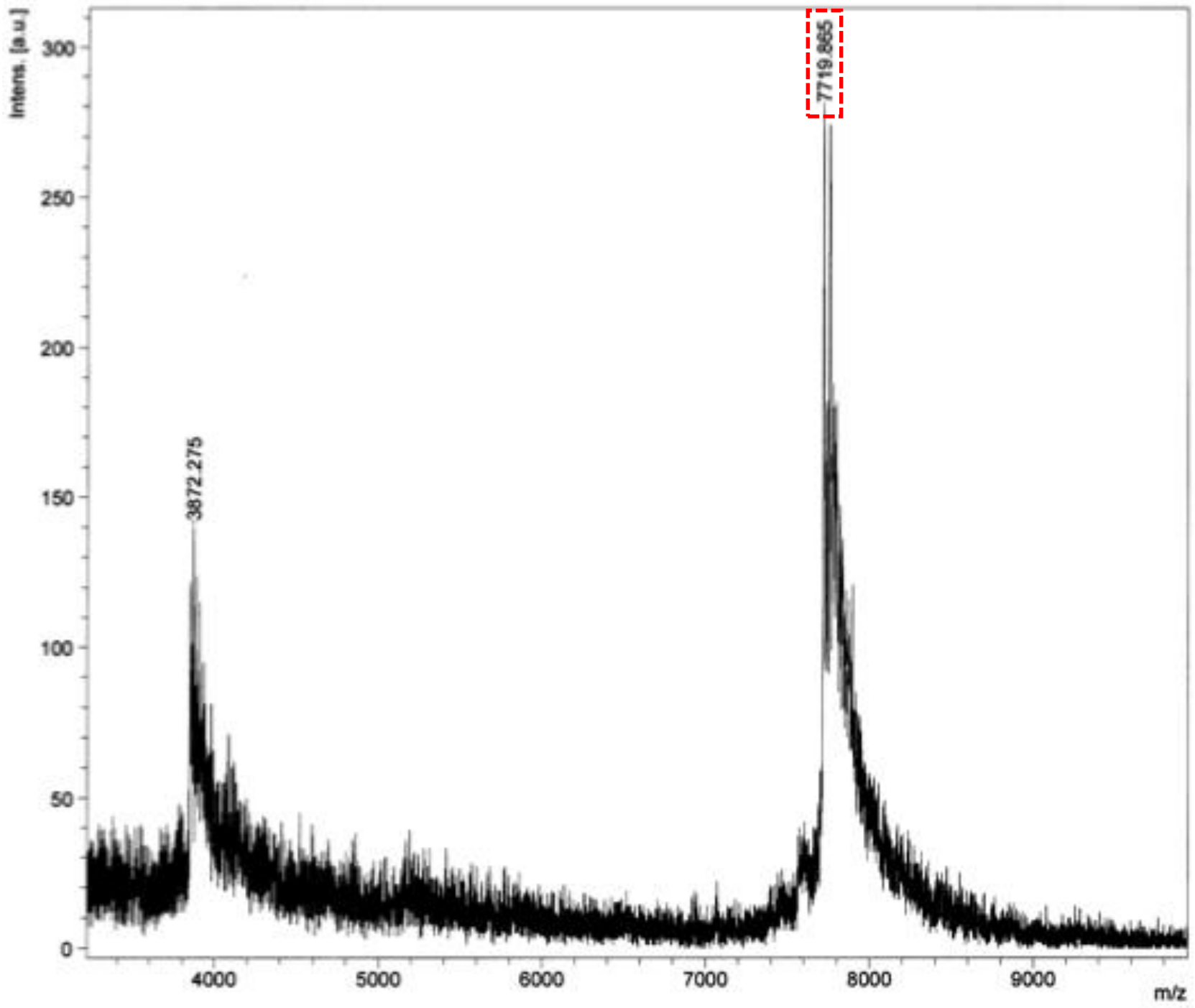

ss-BDP

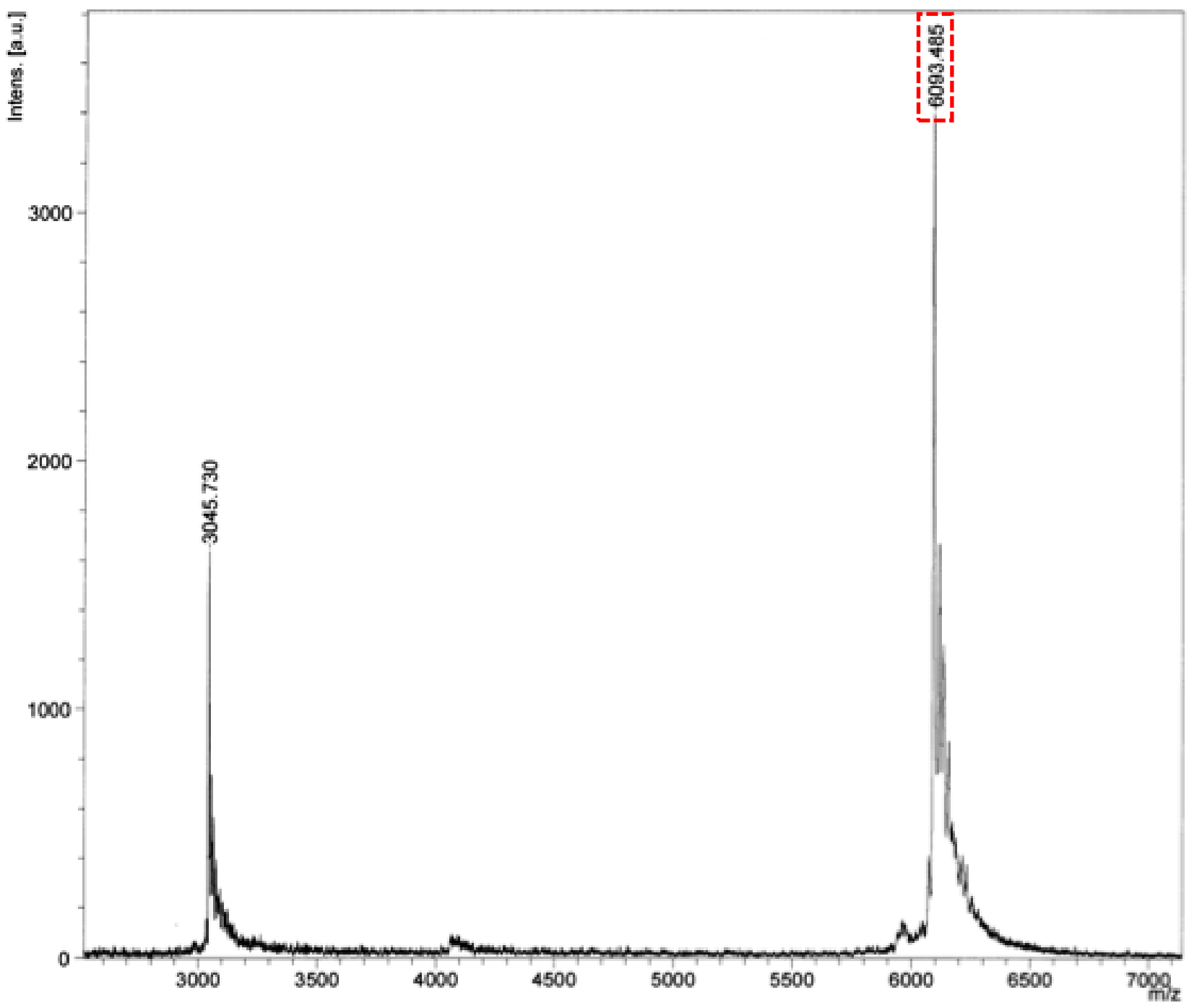

Supplement: Supplementary file 2 [file 42004_2026_2170_MOESM2_ESM.pdf]
