# Supplementary material for: Photocatalytic proximity labeling for the identification of G-quadruplex DNA-interacting proteins
Source: Commun Chem. 2026 Aug 27;9:286. doi: 10.1038/s42004-026-02170-9 (PMC13522520; doi:10.1038/s42004-026-02170-9)
Supplement: Supplementary file 3 [file 42004_2026_2170_MOESM3_ESM.pdf]

Description of Additional Supplementary Files

File name: Supplementary Data 1
Description: List of the identified G4-interacting protein candidates by quantitative proteomics analysis for G4-Ru versus controls.

File name: Supplementary Data 2
Description: List of the identified G4-interacting protein candidates by quantitative proteomics analysis for G4-BDP versus controls.

File name: Supplementary Data 3
Description: Results of the Gene Ontology (GO) functional enrichment analysis of the identified G4-interacting proteins.
